# Systematic review and meta−analysis of PET−based prognostic metrics in CAR−T treatment of DLBCL

**DOI:** 10.3389/fimmu.2026.1679181

**Published:** 2026-02-24

**Authors:** Chirong Long, Xu Liu, Hanqi Fang, Haomiao Qi, Chunyu Ma, Huaizhi Wang, Zhenghong Li

**Affiliations:** 1 School of Basic Medical Sciences, Bengbu Medical University, Bengbu, Anhui, China; 2 School of clinical medicine, Bengbu Medical University, Bengbu, Anhui, China

**Keywords:** ^18^F-fluorodeoxyglucose positron emission tomography/computed tomography, chimeric antigen receptor T-cell, diffuse large B-cell lymphoma, meta-analysis, systematic review.

## Abstract

**Objectives:**

This study aims to conduct a systematic review and meta-analysis to investigate how imaging parameters derived from ^18^F-FDG positron emission tomography/computed tomography (PET/CT) predict treatment outcomes in patients with diffuse large B-cell lymphoma (DLBCL) receiving chimeric antigen receptor T-cell (CAR-T) therapy.

**Methods:**

A comprehensive search was conducted in PubMed, Embase, Cochrane Library, and Web of Science databases to retrieve relevant literature from their inception to December 24, 2024. This study is registered in PROSPERO (CRD42025634694). The protocol was completed in accordance with the PRISMA guidelines recommended by the EJNMMI authors’ guide. Cohort studies were included that enrolled patients diagnosed with DLBCL via ^18^F-FDG PET/CT and who received CAR-T therapy. Fixed-effect and random-effects models were applied using Stata software to calculate pooled hazard ratios (HR) with 95% confidence intervals (CI). Heterogeneity was assessed using the I² statistic.

**Results:**

A total of 14 studies were included, involving 1,088 patients (aged 20 to 86 years) diagnosed with DLBCL based on ^18^F-FDG PET/CT imaging findings. Univariate analysis demonstrated significant associations between several PET-derived parameters and survival outcomes: SUVmax was predictive of both overall survival (OS) (HR: 1.61; 95% CI: 1.20–2.18) and progression-free survival (PFS) (HR: 1.47; 95% CI: 1.09–1.98); higher MTV levels were associated with decreased OS (HR: 2.81; 95% CI: 1.23–6.45) and PFS (HR: 2.39; 95% CI: 1.24–4.61); and TMTV and TLG were also prognostic for PFS and OS. Notably, elevated LDH was linked to inferior OS (HR: 2.76; 95% CI: 2.06–3.71) and PFS (HR: 1.95; 95% CI: 1.50–2.54). ECOG performance status (HR: 2.14; 95% CI: 1.38–3.31) and DS (HR: 6.02; 95% CI: 2.80–12.94) were significantly associated with OS, while IPI was also predictive of OS (HR: 2.04; 95% CI: 1.19–3.50). Elevated LDH and impaired ECOG performance status were independently linked to poorer OS in multivariate analysis (HRs: 3.52 and 2.58, respectively), while the IPI score remained a standalone determinant of PFS (HR: 3.07; 95% CI: 1.59–5.93).

**Conclusion:**

The outcomes of DLBCL cases managed using CAR-T cells can be effectively predicted using both metabolic metrics from ^18^F-FDG PET/CT and conventional clinical prognostic markers.

**Systematic Review Registration:**

https://www.crd.york.ac.uk/prospero, identifier CRD42025634694.

## 1 Introduction

Diffuse large B-cell lymphoma (DLBCL) is the most frequently diagnosed histological subtype of non-Hodgkin lymphoma (NHL), comprising roughly 30–40% of global cases (1). In recent years, both incidence and mortality associated with DLBCL have shown an upward trend (2). R-CHOP, a regimen based on anthracycline and including rituximab + cyclophosphamide + doxorubicin (also known as hydroxydaunorubicin) + vincristine (Oncovin) + prednisone, is widely accepted as the standard initial therapy (3). While R-CHOP induces complete remission in nearly two-thirds of patients (4), the overall five-year survival remains unsatisfactory, with rates as low as 20–30% (5). Due to DLBCL’s highly heterogeneous nature, aggressive behavior, and rapid progression, many patients experience relapse or disease progression during or after treatment (6–8). These challenges underscore the urgent need to explore alternative or adjunctive treatment approaches that could improve patient prognosis.

Chimeric antigen receptor T-cell (CAR-T) therapy has led to major shifts in cancer treatment strategies and was recognized among the top 10 scientific breakthroughs of 2013 by Science (9). The initial concept was introduced by Gross et al. in 1989 (10). Since 2017, three CAR-T therapies—Yescarta, Kymriah, and Breyanzi—have been authorized by the U.S. Food and Drug Administration (FDA) for managing DLBCL (11–13). Numerous clinical studies have reported encouraging results for CAR-T treatment in cases of relapsed or refractory DLBCL (14–16). However, as CAR-T is a personalized treatment, the therapeutic response and prognosis can vary among DLBCL patients. This variability highlights the necessity of refining prognostic assessment tools and optimizing therapeutic strategies. On the one hand, prediction can help clinicians assess patient survival and tailor more effective treatment plans, as DLBCL patients may respond differently to therapy. On the other hand, for patients who have already undergone CAR-T treatment, prognostic prediction may guide adjustments to the treatment regimen, minimizing the risk of ineffective therapy along with its related adverse reactions.

A recent systematic review and meta-analysis demonstrated (17) that ^18^F-fluorodeoxyglucose positron emission tomography/computed tomography (^18^F-FDG PET/CT) parameters serve as important tools for predicting the prognosis of lymphoma patients receiving CAR-T cell therapy. ^18^F-FDG PET/CT also holds significant value in predicting CAR-T cell therapy–related toxicity and treatment response (18, 19). Studies indicate that the role of ^18^F-FDG PET/CT in monitoring treatment efficacy and assessing prognosis in DLBCL is increasingly recognized (20). A range of metabolic indices derived from PET/CT—such as maximum standardized uptake value (SUVmax), change in SUVmax (ΔSUVmax), metabolic tumor volume (MTV), change in MTV (ΔMTV), total metabolic tumor volume (TMTV), total lesion glycolysis (TLG), and change in TLG (ΔTLG)—have been proposed as potential predictors of patient outcomes following CAR-T therapy (21, 22). In addition, several clinical indicators, including lactate dehydrogenase (LDH), Eastern Cooperative Oncology Group (ECOG) score, Deauville score (DS), International Prognostic Index (IPI), and International Metabolic Prognostic Index (IMPI), have also been linked to survival outcomes (23, 24). However, some studies have reported contradictory findings regarding the predictive value of these parameters (25, 26). Given the discrepancies across studies, a structured method for synthesizing evidence is warranted. Systematic reviews and meta-analyses, recognized as the highest tier of evidence in evidence-based medicine, are crucial in integrating multiple study outcomes through rigorous literature screening and statistical analysis, allowing for the formulation of scientifically sound and reliable conclusions, which are well worth referencing in clinical decision-making.

Accordingly, the present study seeks to provide a comprehensive synthesis through systematic review and meta-analysis regarding the assessment of PET-guided prognostic factors in CAR-T-treated DLBCL patients. By integrating data from diverse clinical studies, high-quality evidence is provided to support individualized treatment planning and to inform future clinical practice with greater scientific rigor.

## 2 Methods

### 2.1 Registration

In conducting this systematic review, the PRISMA 2020 (Preferred Reporting Items for Systematic Reviews and Meta-Analyses) checklist was strictly followed (27), with additional details provided in the **Supplementary Materials** (**Supplementary Table S1**, **Supplementary Figure S1**). This protocol was registered in advance on the PROSPERO platform (https://www.crd.york.ac.uk/prospero) under the identifier CRD42025634694.

### 2.2 Literature search

An extensive, protocol-driven search was implemented via PubMed, Embase, Cochrane Library, and Web of Science databases. from their respective inceptions through December 24, 2024. The search strategy incorporated controlled vocabulary terms such as MeSH and Emtree, alongside relevant free-text keywords including “chimeric antigen receptor T-cell therapy”, “diffuse large B-cell lymphoma”, and “positron emission tomography/computed tomography”. Terms were systematically linked using the logical operators AND and OR. Initial database queries and screening procedures were independently executed by two reviewers (Chirong Long and Xu Liu), who performed eligibility assessment by reviewing titles, abstracts, and entire articles. A third reviewer (Hanqi Fang) was consulted to resolve any screening disagreements. The full details of the search methodology can be found in **Supplementary Table S2**.

### 2.3 Eligibility criteria

Inclusion was restricted to studies that satisfied (1): Patients with DLBCL whose outcomes after CAR-T cell therapy were predicted using ^18^F-FDG PET/CT (2); Investigated prognostic markers such as SUVmax, ΔSUVmax, MTV, ΔMTV, TMTV, TLG, ΔTLG, LDH, ECOG, DS, IPI, and IMPI (3); Overall survival (OS) and progression-free survival (PFS) were among the survival outcomes reported (4); Study design: cohort study (5); Included adult participants (≥18 years) (6); Published in English. The following conditions resulted in study exclusion: (a) Studies enrolling patients with malignancies other than DLBCL; (b) Duplicates or conference abstracts; (c) *In vitro* or animal model studies; (d) Case reports, reviews, poster presentations, and letters; (e) Reviews and meta-analyses; (f) Studies lacking sufficient data to extract hazard ratios (HRs); (g) Studies with no access to full text or insufficient data in the full text.

### 2.4 Data collection

Two reviewers, Chirong Long and Xu Liu, independently extracted data, including first author, year of publication, country, sample size, patient age, disease severity, CAR-T intervention details, baseline requirements, ^18^F-FDG PET/CT metrics, clinical prognostic indicators, hazard ratios (HRs) related to overall and progression-free survival, and study design type. The primary outcome measures were SUVmax, ΔSUVmax, MTV, ΔMTV, TMTV, TLG, and ΔTLG. The secondary outcome measures included LDH, ECOG, DS, IPI, and IMPI.

### 2.5 Quality assessment

The methodological quality of the included studies was independently evaluated by two authors, Chirong Long and Xu Liu. Differences in opinion were settled through discussion. We used the cohort study component of the NIH (National Institutes of Health) quality assessment scale (https://www.nhlbi.nih.gov/health-topics/study-quality-assessment-tools) as the tool for evaluating study quality, which includes ten scoring domains (Q1–Q10). The quality ratings assigned to studies ranged from low (1–3), moderate (4–6), to high (7–10) based on their scores.

### 2.6 Statistical analysis

Stata (version 18) was employed to carry out the analysis of data from each study selected. I² was employed to evaluate heterogeneity among the studies. A fixed-effects model was used when I² was 50% or less; otherwise, a random-effects model was employed. HRs and 95% confidence intervals (CIs) were pooled to analyze the correlation between PET/CT indicators and survival outcomes (OS and PFS). We conducted a sensitivity analysis on the article using the “leave-one-out” method, in order to determine whether the outcome of the article was stable. Forest plots were generated to visualize effect sizes. As fewer than 10 original studies were available for each indicator, no analysis of publication bias was performed. In instances of potential bias, trim-and-fill is recommended. Significance was assigned to *p*-values <0.05.

## 3 Results

### 3.1 Mechanistic basis of PET/CT imaging and CAR-T cell–mediated tumor elimination

**Figure 1:**
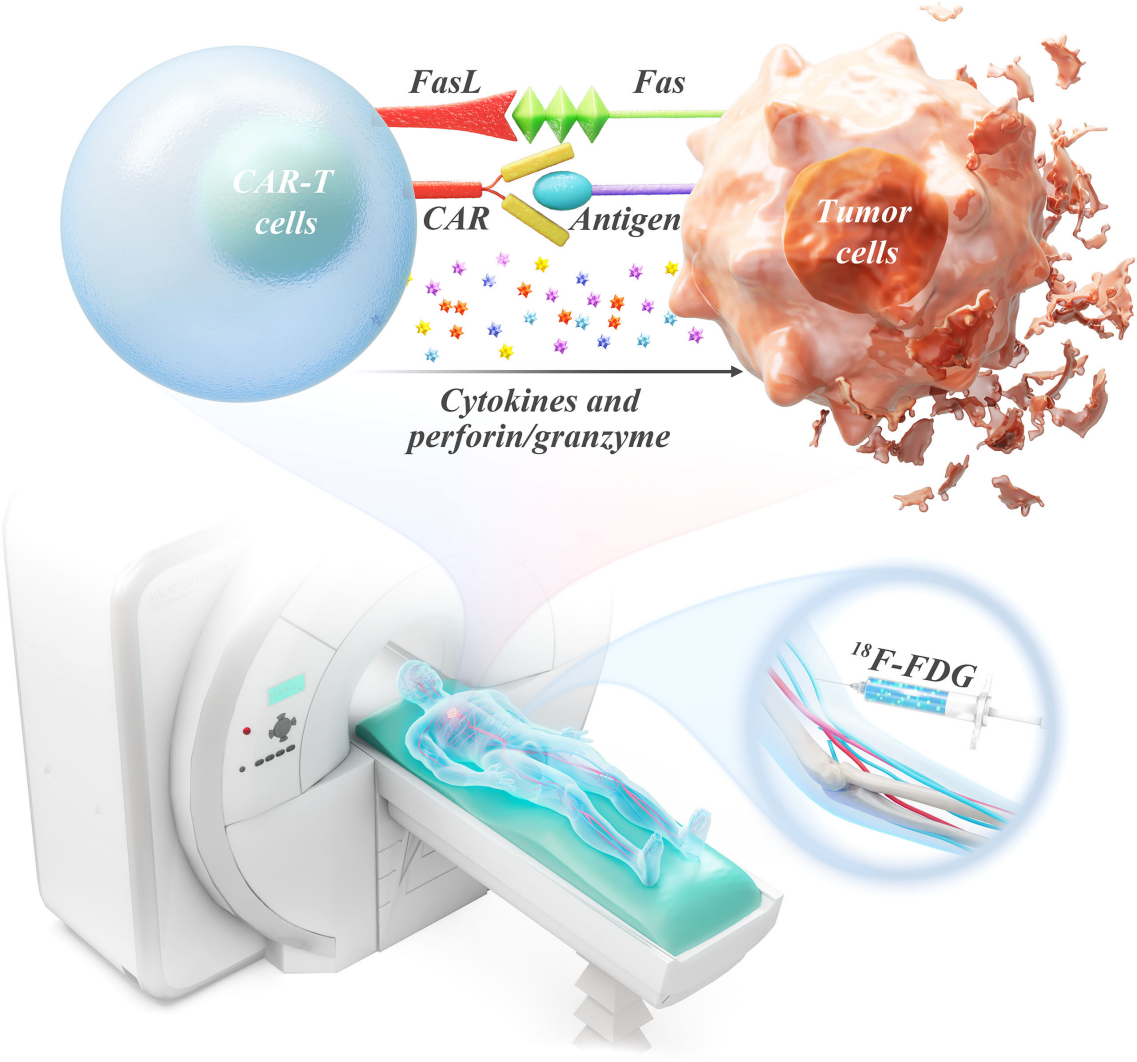
Mechanisms of PET/CT imaging and tumor cell killing by CAR-T cells.

**Figure 1** illustrates the mechanisms underlying PET/CT imaging and the antitumor effects mediated by CAR-T cells (28). These mechanisms involve recognition, activation, and killing. The chimeric antigen receptor (CAR) on CAR-T cells enables them to specifically bind to tumor-associated antigens expressed on malignant cells. Activation of CAR-T cells upon antigen recognition promotes swift clonal expansion and the onset of cytolytic activities such as Fas/FasL-mediated signaling and the discharge of cytotoxic granules (perforin and granzyme), as well as pro-inflammatory cytokines that facilitate tumor cell apoptosis.

### 3.2 Overview of included studies

**Figure 2:**
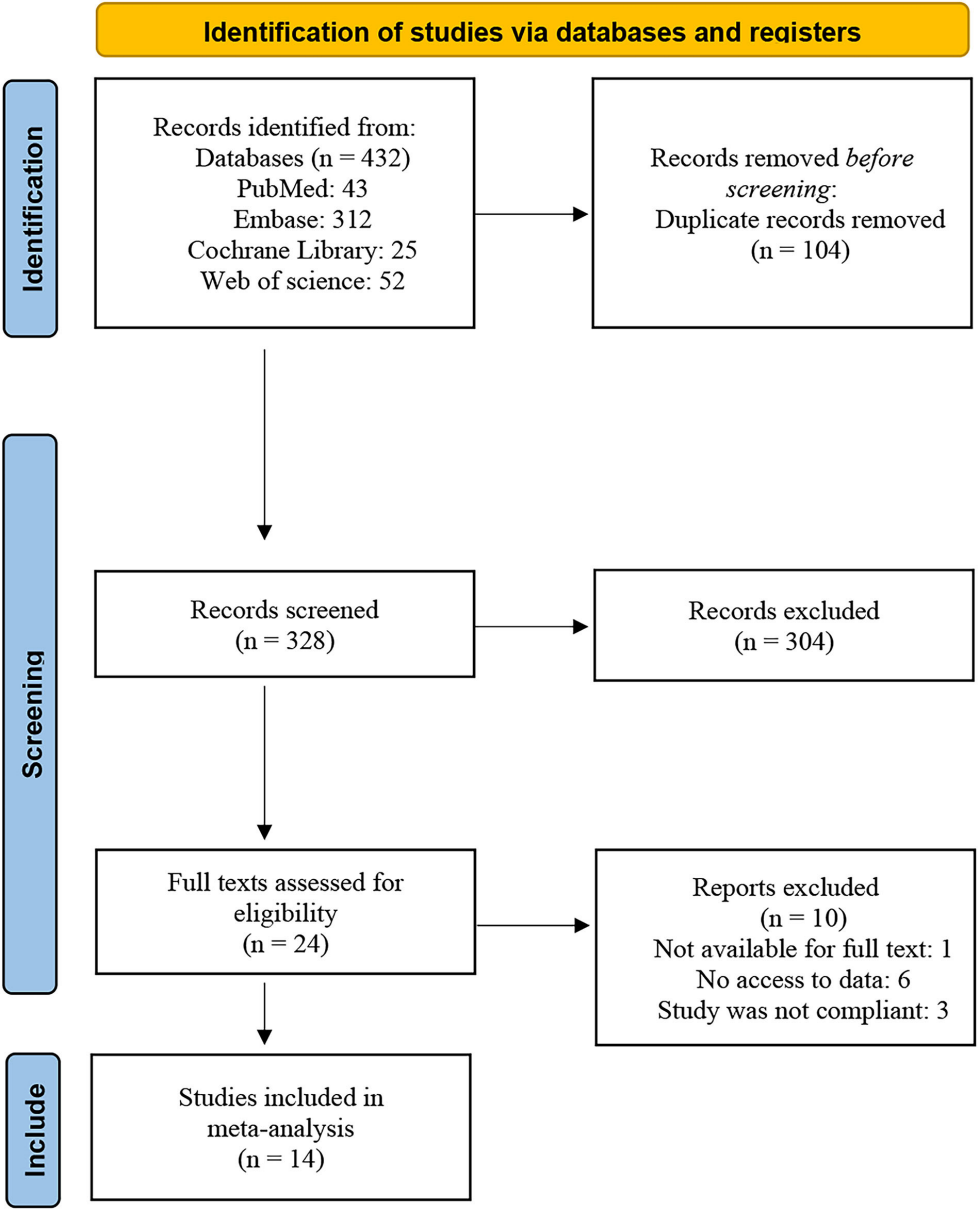
Flow diagram of selection methodology and exclusion rationales.

An initial search identified 432 records. After 104 duplicates were removed, 328 records were retained. Screening of titles and abstracts led to the elimination of 304 studies. After further examination, 10 out of the 24 full-text articles were excluded. Ultimately, 14 studies involving 1,088 DLBCL patients met the inclusion criteria and were analyzed. Following PRISMA 2020 standards (https://www.prisma-statement.org/), **Figure 2** illustrates the process of selecting studies and the justification for their exclusion.

Among them, 14 studies (22, 24, 29–40) reported OS, and 13 (22, 24, 30–40) reported PFS. Disease severity reported in the studies included neurotoxicity, cytokine storm, relapse, progression, and death. Patient ages ranged from 20 to 86 years. Protocols for ^18^F-FDG varied, but most studies required fasting for over 6 hours before tracer administration and pre-scan blood glucose testing. The majority of the studies (93%) were retrospective in design, with only one being prospective (34). Further information on patient characteristics and study design is summarized in **Table 1**.

**Table 1:** The basic characteristics of the included studies.

Author and year	Country	Gender (M/F)	Sample size	Age	Median follow-up time	Survival time	Description of disease severity	Name of the intervention	Doses of interventions	The requirements of the patient	Parameter	Ending	Design type	NIH quality assessment
Wang J 2019	China	M	12	43 [22-67]	5 months	9.8 months	CS, SVCS neurotoxicity, local complications	^18^F-FDG PET/CT	(4.44-5.55) MBq/kg	Fasting was≥6 h before injection	MTV	OS	retrospective analysis	High
		F	7											
Figura NB 2021	USA	M	46	64 [28-76]	12.6 months	7.4 months	PD, Death	^18^F-FDG PET/CT	N/A	N/A	SUVmax, MTV	OS, PFS	retrospective analysis	High
		F	17											
Sesques P 2021	France	M	44	60 [27-78]	NR/NI	OS:3.7-11.7 months PFS:2.7-6.8 months	CRS, ICANS, PD, Death	^18^F-FDG PET/CT	3 MBq/kg	Fasting≥6 h prior to injection, blood glucose level<150 mg/dL	SUVmax, MTV, ΔMTV, ΔTLG, LDH, ECOG	OS, PFS	retrospective analysis	High
		F	28											
Cohen D 2022	Israel	M	25	68 [61-76]	12.8 months	NR/NI	CRS, ICANS, Death	^18^F-FDG PET/CT	3.7 MBq/kg	N/A	SUVmax, TMTV, LDH, ECOG	OS, PFS	retrospective analysis	High
		F	23											
Winkelmann M 2023	Germany	M	24	67	NR/NI	PFS:2.9-6.2 months	PD	^18^F-FDG PET/CT	(2.5-4.5) MBq/kg (159-275 MBq)	N/A	ECOG, IPI	OS, PFS	retrospective analysis	High
		F	15											
Galtier J 2023	France	M	99	63 [21-77]	12.6 months	OS:22.1 months PFS:4.6 months	Recurrence, Progression, Death	^18^F-FDG PET/CT	N/A	N/A	ΔSUVmax, MTV, TMTV, LDH, DS	OS, PFS	retrospective analysis	High
		F	61											
Guidetti A 2023	Italy	M	32	55 [22-70]	12 months	NR/NI	PD, Death	^18^F-FDG PET/CT	3-4 MBq/kg	Fasting≥6 h prior to injection, blood glucose level<200 mg/dL	ΔSUVmax, ΔMTV, ΔTMTV, ΔTLG, DS	OS, PFS	prospective study	High
		F	15											
Ligero M 2023	Spain	M	63	59 [50–68]	17.6 months	OS:15.4 months PFS:3.9 months	CRS, ICANS, Death	^18^F-FDG PET/CT	3.7 MBq/kg (222-370 MBq)	Fasting≥6 h prior to injection, blood glucose values<142 mg/dL	SUVmax, ΔMTV, TMTV, ECOG	OS, PFS	retrospective analysis	High
		F	30											
Zhou Y 2023	China	M	37	53 [26-75]	NR/NI	NR/NI	Tumor recurrence, Death, CRS	^18^F-FDG PET/CT	(4.07-5.56) MBq/kg	Fasting≥6 h prior to injection, blood glucose level<11 mmol/L	MTV, TLG, LDH, IPI, ECOG, DS	OS, PFS	retrospective analysis	High
		F	24											
Ababneh HS 2025	USA	M	22	63 [37-81]	11.7 months	OS:14 months PFS:2.6 months	Death	^18^F-FDG PET/CT	N/A	N/A	SUVmax, MTV, TLG, LDH, ECOG, IPI	OS, PFS	retrospective analysis	High
		F	11											
Alderuccio JP 2024	USA, Thailand, Italy, Switzerland	M	82	60	NR/NI	OS:5.4-19.2 months PFS:2.8-15 months	Disease recurrence or progression, unacceptable toxicity, death	^18^F-FDG PET/CT	N/A	N/A	SUVmax, MTV, TLG, LDH, IMPI	OS, PFS	retrospective analysis	High
		F	63											
Gui J 2024	China	M	23	55 [29-74]	18.2 months	OS:NR/NI PFS:11.5 months	Toxicity, PD, CRS, Death	^18^F-FDG PET/CT	(3.70–5.55) MBq/kg	Fasting≥6 h before injection, blood glucose level≤11.1 mmol/L	SUVmax, ΔSUVmax, MTV, ΔMTV, TLG, ΔTLG, ΔTTLG, LDH, ECOG, DS, IPI	OS, PFS	retrospective analysis	High
		F	15											
Leithner D 2024	USA, Austria, Spain	M	121	66 [20-86 ]	NR/NI	NR/NI	CRS, ICANS, Death	^18^F-FDG PET/CT	444 MBq±10%	The blood glucose level was less than 182 mg per deciliter	SUVmax, MTV, TLG	OS, PFS	retrospective analysis	High
		F	59											
Sheng LS 2024	China	M	53	56.5	NR/NI	NR/NI	CRS	^18^F-FDG PET/CT	N/A	N/A	MTV, TMTV, TLG, LDH, ECOG	OS, PFS	retrospective analysis	High

F, female; M, male; CS, cytokine storm; SVCS, superior vena cana syndrome; PD, progression of disease; CRS, cytokine release syndrome; ICANS, neurotoxic syndrome associated with immune effector cells; N/A, not applicable; 18F-FDG, 18F-fluorodeoxyglucose; PET/CT, positron emission tomography/computed tomography; SUVmax, maximum standardized uptake value; ΔSUVmax, change in maximum standardized uptake value; MTV, metabolic tumor volume; ΔMTV, The amount of change in tumor metabolic volume; TMTV, total metabolic tumor volume; TLG, total lesion glycolysis; ΔTLG, the amount of change in total lesion glycolysis; LDH, lactate dehydrogenase; ECOG, eastern cooperative oncology group; DS, deauville score; IPI, international prognostic index; IMPI, international metabolic prognostic index; OS, overall survival; PFS, progression-free survival.

### 3.3 Quality appraisal

The National Institutes of Health (NIH) instrument for evaluating study quality was applied in this review, with scores classified into low (1–3), moderate (4–6), and high (7–10) categories. Among the 14 included studies, three studies achieved a score of 8 (30, 39, 40), four received a score of 9 (24, 29, 35, 38), and seven obtained the highest score of 10 (22, 31–34, 36, 37). In the quality assessment, a score of 0 was most commonly assigned to questions Q7 or Q8. Overall, the included studies had a quality score of ≥8, classifying them as high quality. **Table 2** presents the detailed evaluation scores.

**Table 2:** NIH quality assessment form.

Study	Q1	Q2	Q3	Q4	Q5	Q6	Q7	Q8	Q9	Q10	Total
Wang J 2019	1	1	1	1	1	1	0	1	1	1	9
Figura NB 2021	1	1	1	1	1	1	0	0	1	1	8
Sesques P 2021	1	1	1	1	1	1	1	1	1	1	10
Cohen D 2022	1	1	1	1	1	1	1	1	1	1	10
Winkelmann M 2023	1	1	1	1	1	1	1	0	1	1	9
Galtier J 2023	1	1	1	1	1	1	1	1	1	1	10
Guidetti A 2023	1	1	1	1	1	1	1	1	1	1	10
Ligero M 2023	1	1	1	1	1	1	0	1	1	1	9
Zhou Y 2023	1	1	1	1	1	1	1	1	1	1	10
Alderuccio JP 2024	1	1	1	1	1	1	1	0	1	1	9
Gui J 2024	1	1	1	1	1	1	1	1	1	1	10
Leithner D 2024	1	1	1	1	1	1	0	0	1	1	8
Sheng LS 2024	1	1	1	1	1	1	0	0	1	1	8
Ababneh HS 2025	1	1	1	1	1	1	1	1	1	1	10

NIH, National Institutes of Health; Q1, Was the research question or objective in this paper clearly stated?; Q2, Was the study population clearly specified and defined?; Q3, Was the participation rate of eligible persons at least 50%?; Q4, Were all the subjects selected or recruited from the same or similar populations (including the same time period)?; Were inclusion and exclusion criteria for being in the study prespecified and applied uniformly to all participants? Q5, Was a sample size justification, power description, or variance and effect estimates provided? Q6, For exposures that can vary in amount or level, did the study examine different levels of the exposure as related to the outcome (e.g., categories of exposure, or exposure measured as continuous variable)? Q7, Were the exposure measures (independent variables) clearly defined, valid, reliable, and implemented consistently across all study participants? Q8, Were the outcome measures (dependent variables) clearly defined, valid, reliable, and implemented consistently across all study participants? Q9, Were the outcome assessors blinded to the exposure status of participants? Q10, Were key potential confounding variables measured and adjusted statistically for their impact on the relationship between exposure(s) and outcome(s)?

### 3.4 Outcome overview

**Table 3** displays the correlations between ^18^F-FDG PET/CT-derived metabolic metrics, clinical variables, and both OS and PFS in CAR-T-treated DLBCL subjects.

**Table 3:** Pooled HR of ^18^F-FDG PET/CT metabolic parameters and clinical parameters for OS and PFS.

Overall	Univariate analysis					Multivariate analysis				
	*N*	HR(95% CI)	*P*	*I^2^*	Model	*N*	HR(95% CI)	*P*	*I^2^*	Model
OS										
SUVmax	8	1.61 (1.20-2.18)	0.002	79.0%	Random	3	4.69 (0.61-36.03)	0.137	83.1%	Random
MTV	6	2.81 (1.23-6.45)	0.014	91.0%	Random	3	1.61 (0.54-4.82)	0.398	70.4%	Random
TMTV	3	2.12 (0.82-5.46)	0.119	94.5%	Random	2	2.53 (0.80-8.00)	0.113	82.7%	Random
TLG	6	2.14 (1.02-4.49)	0.045	88.3%	Random					
LDH	7	2.76 (2.06-3.71)	0.000	24.3%	Fixed	2	3.52 (1.07-11.54)	0.038	59.0%	Random
ECOG	5	2.14 (1.38-3.31)	0.001	45.7%	Fixed	3	2.58 (1.43-4.63)	0.002	0.0%	Random
DS	2	6.02 (2.80-12.94)	0.000	0.0%	Fixed	3	2.16 (0.10-45.29)	0.620	93.8%	Random
IPI	4	2.04 (1.19-3.50)	0.010	36.7%	Fixed					
IMPI	2	1.73 (0.31-9.75)	0.537	76.3%	Random					
PFS										
SUVmax	7	1.47 (1.09-1.98)	0.011	84.0%	Random	2	2.00 (0.47-8.62)	0.351	75.1%	Random
ΔSUVmax	3	2.21 (0.95-5.13)	0.064	81.6%	Random					
MTV	6	2.39 (1.24-4.61)	0.010	87.4%	Random	2	0.96 (0.28-3.20)	0.941	0.0%	Fixed
ΔMTV	3	2.31 (0.94-5.71)	0.070	75.4%	Random					
TMTV	3	2.60 (1.49-4.52)	0.001	60.0%	Random					
TLG	6	2.65 (1.28-5.51)	0.009	88.7%	Random	2	3.68 (0.95-14.28)	0.060	34.8%	Fixed
ΔTLG	3	1.91 (0.55-6.56)	0.306	88.0%	Random					
LDH	8	1.95 (1.50-2.54)	0.000	0.0%	Fixed	2	0.94 (0.19-4.70)	0.942	74.5%	Random
ECOG	6	1.73 (1.26-2.38)	0.001	41.1%	Fixed					
DS	3	3.04 (0.40-23.35)	0.284	96.4%	Random	2	2.66 (0.18-39.41)	0.476	89.3%	Random
IPI	4	1.53 (0.85-2.75)	0.161	55.4%	Random	2	3.07 (1.59-5.93)	0.001	0.0%	Fixed
IMPI	2	1.75 (0.38-8.08)	0.476	86.6%	Random					

CI, confidence interval; HR, hazard ratio.

### 3.5 Findings from univariate analysis

#### 3.5.1 OS

**Figure 3:**
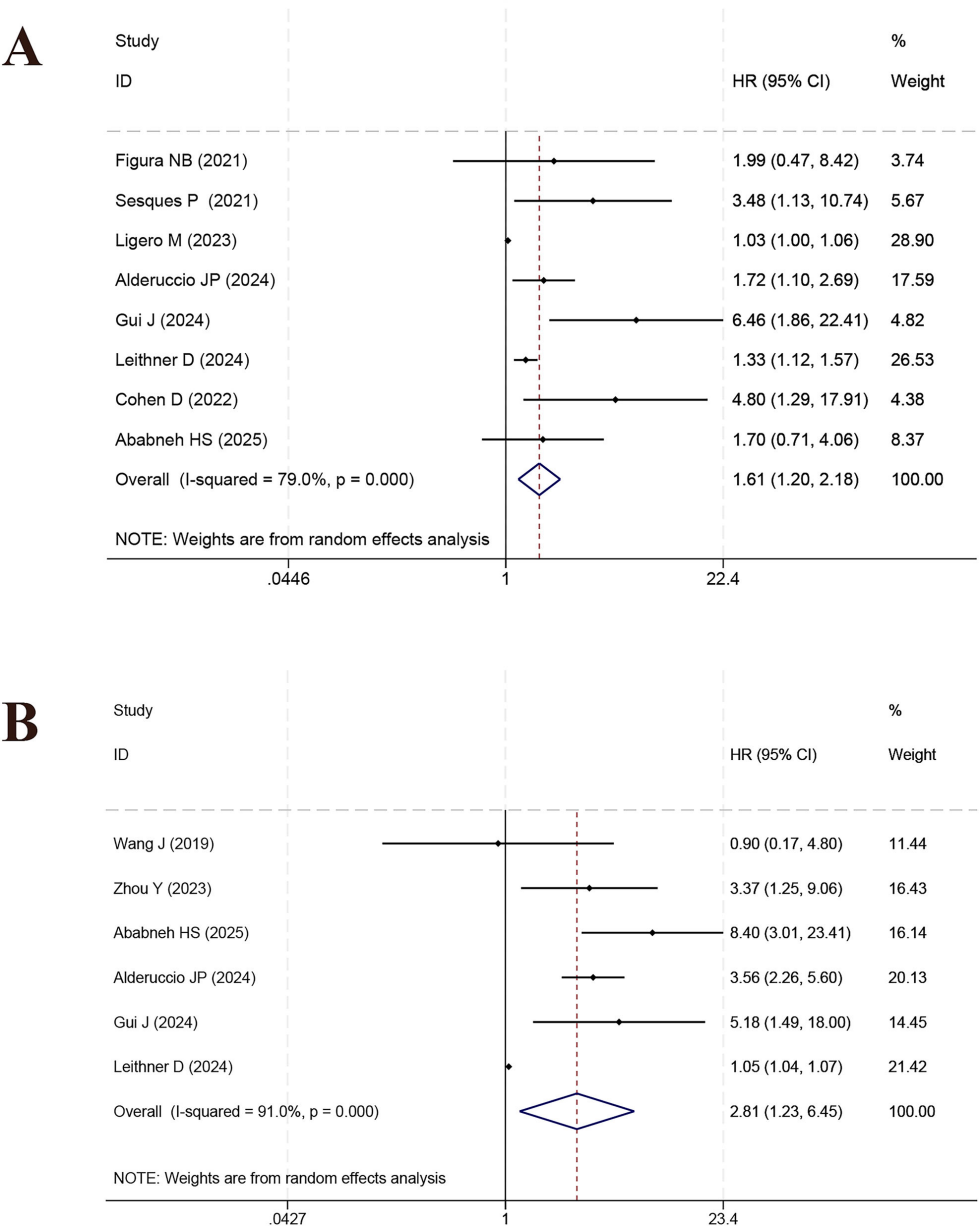
Forest plot of the prognostic effect of SUVmax **(A)** and MTV **(B)** on overall survival.

**Figure 4:**
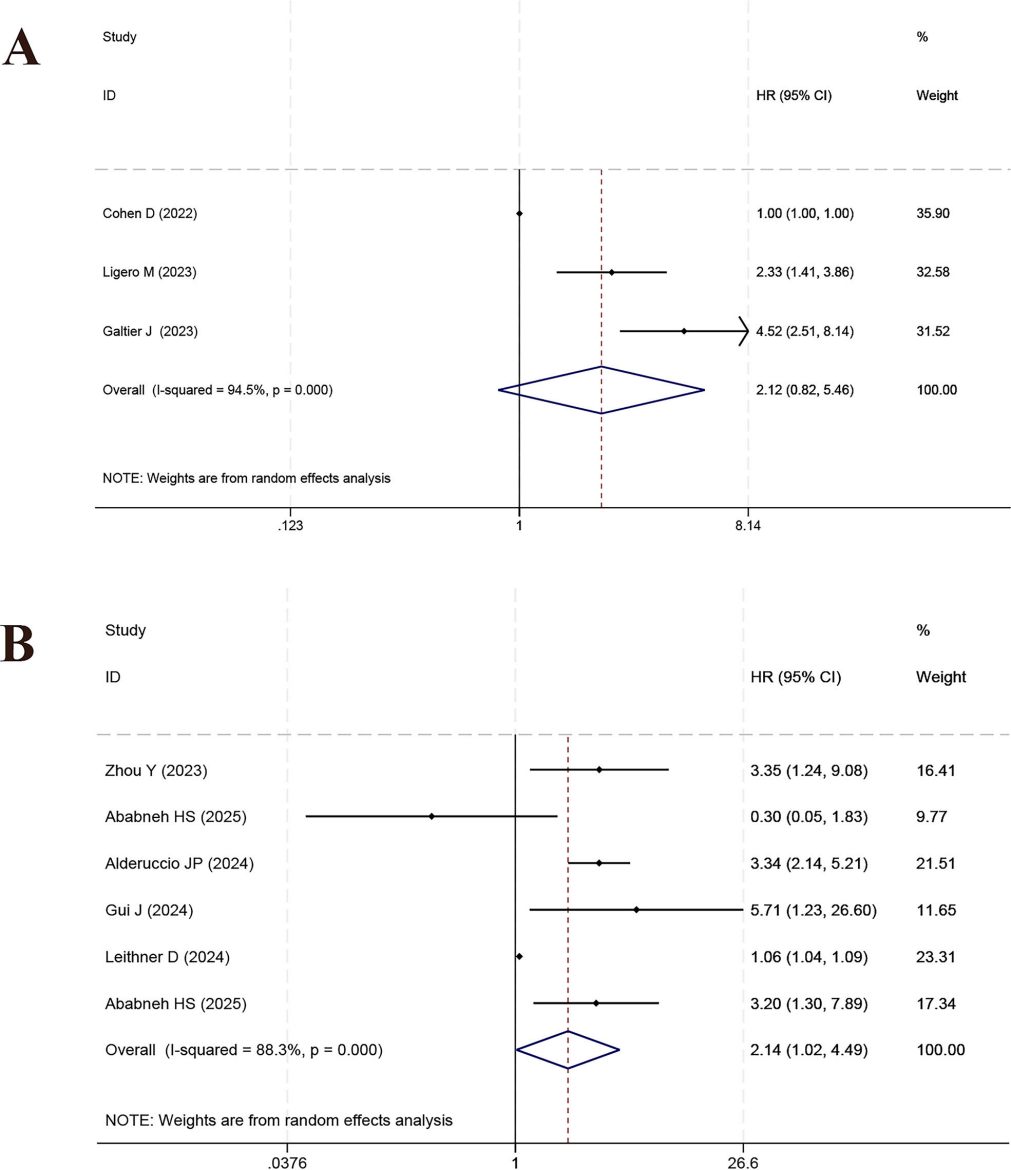
Forest plot of the meta-analysis on the prognostic effect of TMTV **(A)** and TLG **(B)** on overall survival period.

Multiple studies explored the predictive implications of diverse PET/CT-based metabolic and clinical factors in relation to OS among CAR-T-treated DLBCL patients. SUVmax was examined in 8 studies, with pooled analysis indicating a significant link to OS (HR = 1.61, 95% CI: 1.20–2.18, *p* = 0.002; *I²* = 79.0%, **Figure 3A**). Robustness was confirmed via sensitivity analysis (**Supplementary Figure S2A**). MTV, reported in 6 studies, also demonstrated a significant correlation with OS (HR = 2.81, 95% CI: 1.23–6.45, p = 0.014; *I²* = 91.0%, **Figure 3B**; sensitivity analysis in **Supplementary Figure S2B**). Conversely, TMTV, examined in three studies, did not yield statistically significant outcomes (HR = 2.12, 95% CI: 0.82–5.46, *p* = 0.119; *I²* = 94.5%, **Figure 4A**; sensitivity analysis **Supplementary Figure S3A**). Across six studies, TLG was found to be significantly link to OS (HR = 2.14, 95% CI: 1.02–4.49, *p* = 0.045; *I²* = 88.3%, **Figure 4B**; sensitivity analysis in **Supplementary Figure S3B**).

**Figure 5:**
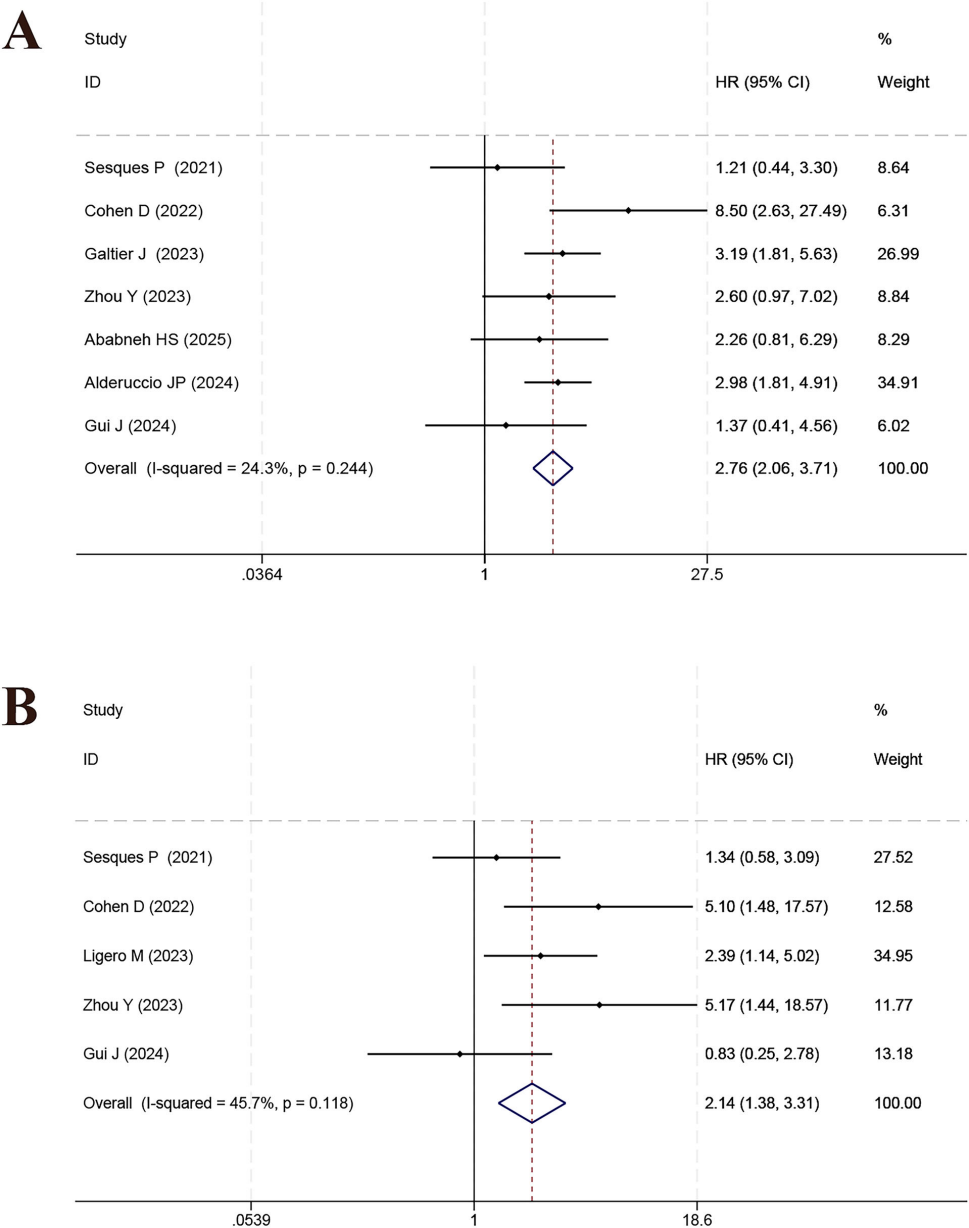
Forest plot of the meta-analysis on the prognostic effect of LDH **(A)** and ECOG **(B)** on overall survival.

**Figure 6:**
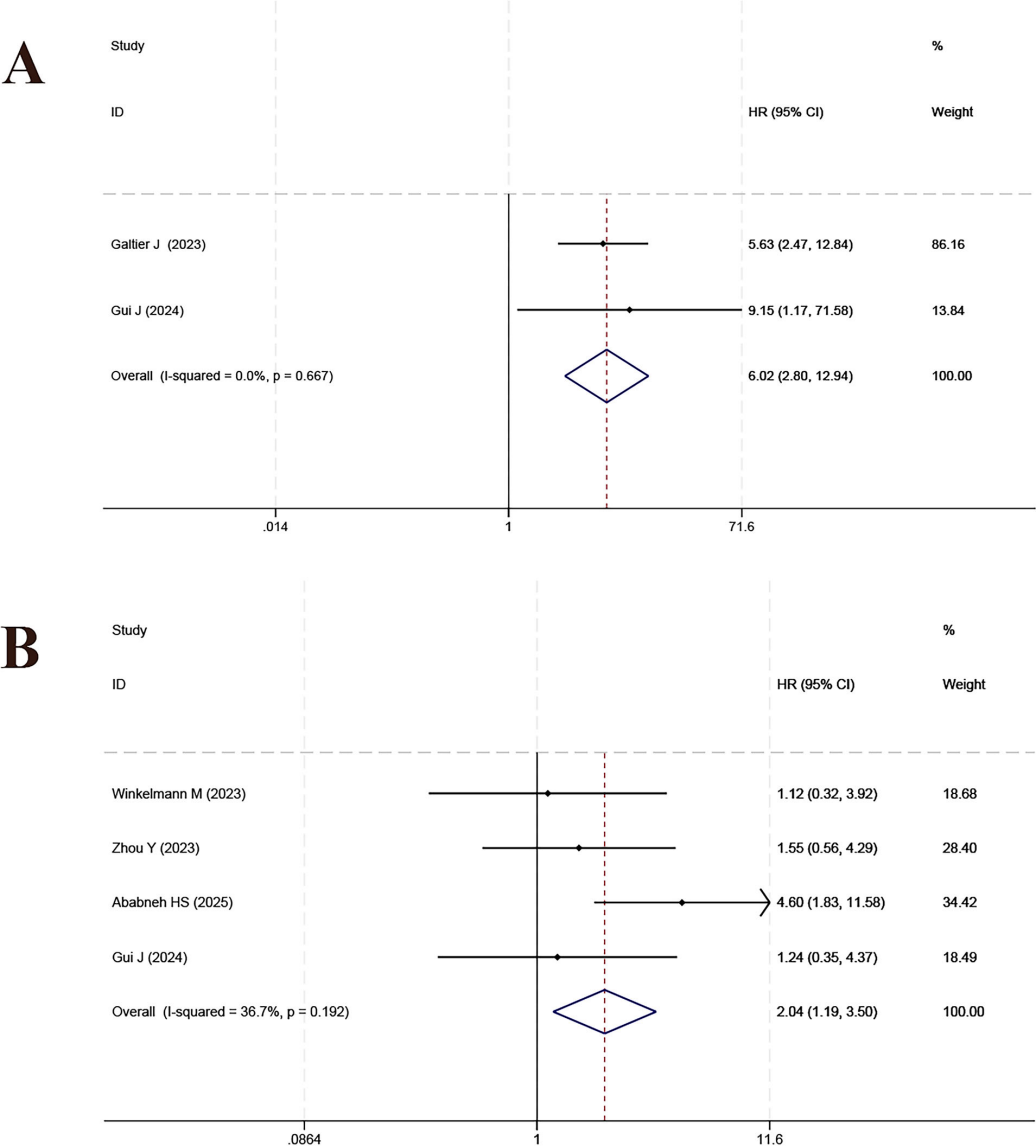
Forest plot of the meta-analysis on the prognostic effect of DS **(A)** and IPI **(B)** on overall survival.

**Figure 7:**
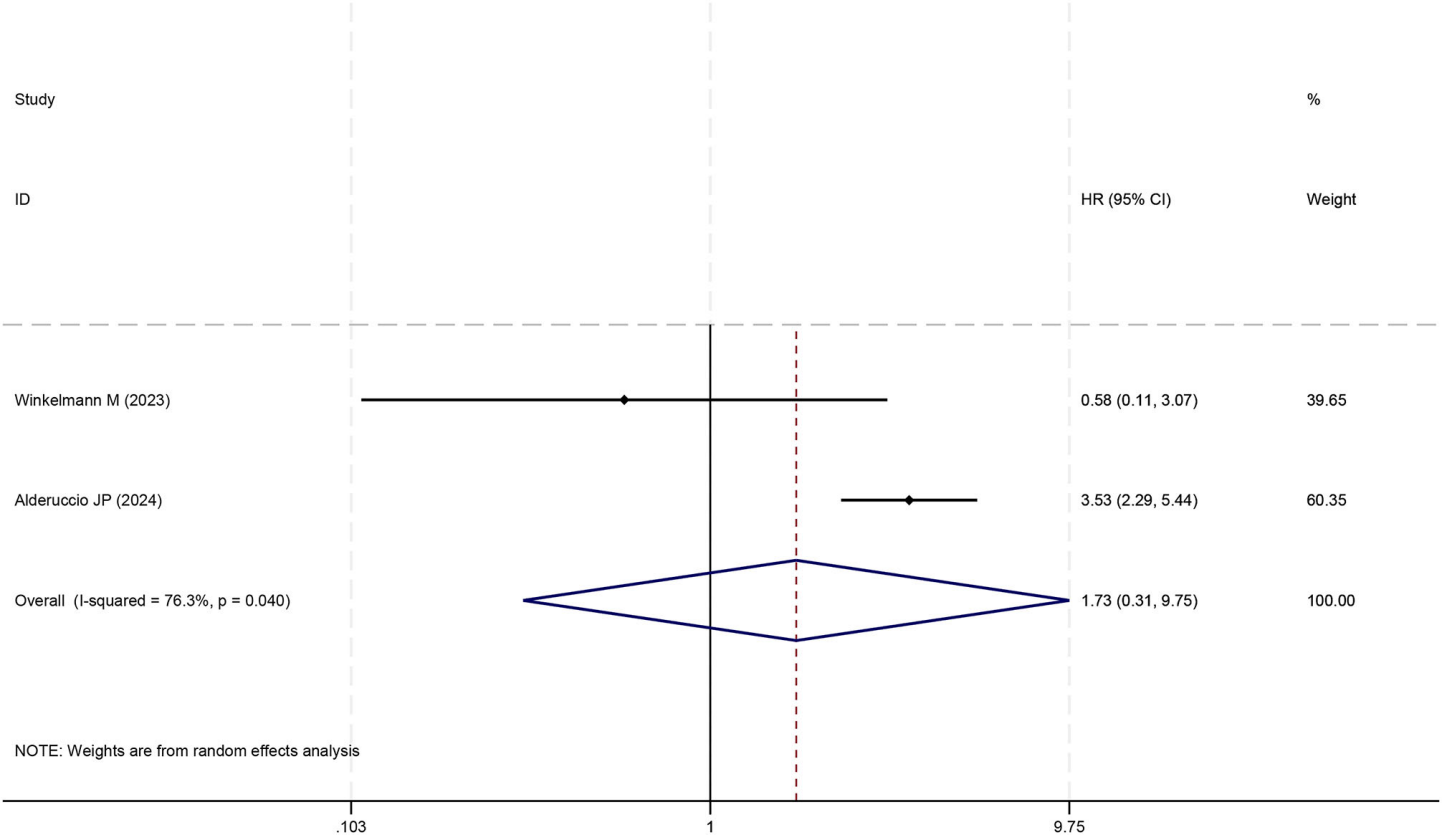
Forest plot of the meta-analysis on the prognostic effect of IMPI on overall survival.

Seven studies identified a significant association between elevated LDH levels and reduced OS (HR = 2.76, 95% CI: 2.06–3.71, *p* < 0.001; I*²* = 24.3%, **Figure 5A**; sensitivity in **Supplementary Figure S4A**). ECOG performance status, evaluated in 5 studies, was also predictive of OS (HR = 2.14, 95% CI: 1.38–3.31, *p* = 0.001; *I²* = 45.7%, **Figure 5B**; see **Supplementary Figure S4B**). DS was examined in 2 studies, both indicating a strong correlation with OS (HR = 6.02, 95% CI: 2.80–12.94, *p* < 0.001; *I²* = 0.0%, **Figure 6A**; sensitivity in **Supplementary Figure S5A**). IPI, based on 4 studies, also showed significant prognostic value (HR = 2.04, 95% CI: 1.19–3.50, *p* = 0.010; *I²* = 36.7%, **Figure 6B**; see **Supplementary Figure S5B**). Analysis from two studies indicated that IMPI was not significantly link to OS (HR = 1.73, 95% CI: 0.31–9.75, *p* = 0.537; *I²* = 76.3%, **Figure 7**; **Supplementary Figure S6**).

Sensitivity analyses for all outcomes indicated the robustness and reliability of the findings. Publication bias was not evaluated, given that fewer than ten studies were available for each individual parameter.

#### 3.5.2 PFS

**Figure 8:**
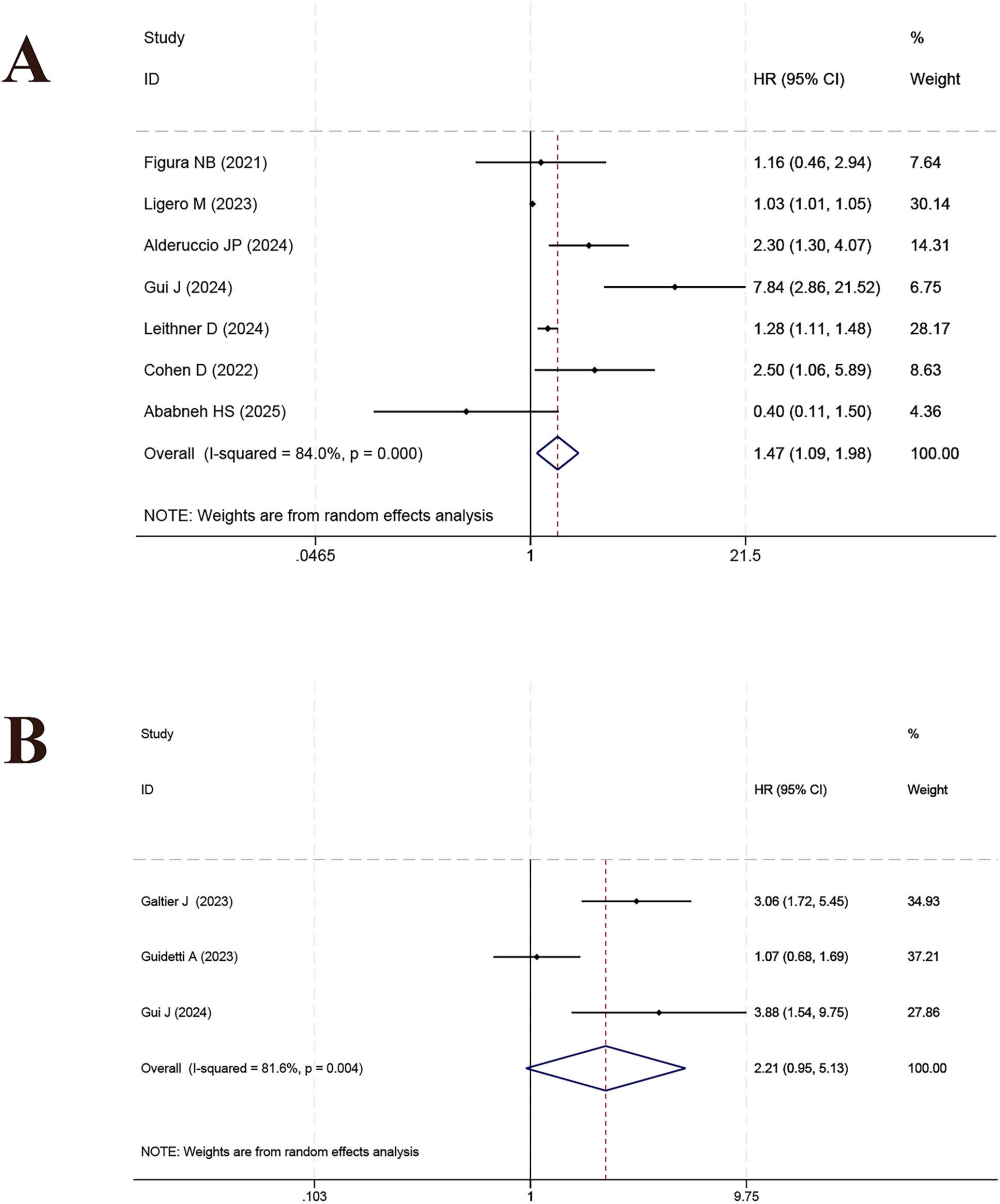
Forest plot of the prognostic effect of SUVmax **(A)** and ΔSUVmax **(B)** on progression-free survival.

SUVmax was examined in 7 studies and was significantly correlated with PFS (HR = 1.47, 95% CI: 1.09–1.98, *p* = 0.011; *I²* = 84.0%, **Figure 8A**; sensitivity analysis in **Supplementary Figure S7A**). No statistically significant findings were observed for ΔSUVmax, as reported in three studies (HR = 2.21, 95% CI: 0.95–5.13, *p* = 0.064; *I²* = 81.6%, **Figure 8B**; sensitivity analysis in **Supplementary Figure S7B**). A significant relationship between MTV and PFS was observed across six studies (HR = 2.39, 95% CI: 1.24–4.61, *p* = 0.010; *I²* = 87.4%, **Supplementary Figure S8A**; sensitivity analysis in **Supplementary Figure S9A**). ΔMTV, reported in 3 studies, showed no significant association (HR = 2.31, 95% CI: 0.94–5.71, *p* = 0.070; *I²* = 75.4%, **Supplementary Figure S8B**; sensitivity analysis in **Supplementary Figure S9B**). A significant relationship between TMTV and PFS was reported in three studies (HR = 2.60, 95% CI: 1.49–4.52, *p* = 0.001; *I²* = 60.0%, **Supplementary Figure S10A**; sensitivity analysis in **Supplementary Figure S11A**). TLG, based on 6 studies, also showed a significant relationship (HR = 2.65, 95% CI: 1.28–5.51, *p* = 0.009; *I²* = 88.7%, **Supplementary Figure S10B**; sensitivity analysis in **Supplementary Figure S11B**). In three studies, ΔTLG did not demonstrate a significant link to PFS (HR = 1.91, 95% CI: 0.55–6.57, *p* = 0.306; *I²* = 88.0%, **Supplementary Figure S12A**; sensitivity analysis in **Supplementary Figure S13A**).

In eight studies, LDH was established as a notable factor in predicting PFS (HR = 1.95, 95% CI: 1.50–2.54, *p* < 0.001; *I²* = 0.0%, **Supplementary Figure S12B**; sensitivity analysis in **Supplementary Figure S13B**). ECOG, from 6 studies, was also a significant predictor (HR = 1.73, 95% CI: 1.26–2.38, *p* = 0.001; *I²* = 41.1%, **Supplementary Figure S14A**; sensitivity analysis in **Supplementary Figure S15A**). Across three studies, DS did not exhibit a notable connection to PFS (HR = 3.04, 95% CI: 0.40–23.35, *p* = 0.284; *I²* = 96.4%, **Supplementary Figure S14B**; sensitivity analysis in **Supplementary Figure S15B**). IPI, assessed in 4 studies, was not found to have a notable connection (HR = 1.53, 95% CI: 0.85–2.75, *p* = 0.161; *I²* = 55.4%, **Supplementary Figure S16A**; sensitivity analysis in **Supplementary Figure S17A**). In the two studies reporting on IMPI, no statistically meaningful link to PFS was noted (HR = 1.75, 95% CI: 0.38–8.08, *p* = 0.476; *I²* = 86.6%, **Supplementary Figure S16B**; sensitivity analysis in **Supplementary Figure S17B**).

The findings were supported by sensitivity analyses across all outcomes, demonstrating their stability and dependability.

### 3.6 Findings from multivariate analysis

#### 3.6.1 OS

Meta-analysis of data from three studies found no statistically significant relationship between SUVmax and OS (HR = 4.69, 95% CI: 0.61–36.03, *p* = 0.137; *I²* = 83.1%, **Supplementary Figure S18A**; sensitivity analysis in **Supplementary Figure S19A**). No significant link between MTV and OS was identified in the three studies analyzed (HR = 1.61, 95% CI: 0.54–4.82, *p* = 0.398; *I²* = 70.4%, **Supplementary Figure S18B**; sensitivity analysis in **Supplementary Figure S19B**). For TMTV, analysis of two studies found no significant relationship with OS (HR = 2.53, 95% CI: 0.80–8.00, *p* = 0.113; *I²* = 82.7%, **Supplementary Figure S20A**; sensitivity analysis in **Supplementary Figure S21A**).

A strong association between LDH levels and reduced OS was reported in two studies (HR = 3.52, 95% CI: 1.07–11.54, *p* = 0.038; *I²* = 59.0%, **Supplementary Figure S20B**; sensitivity analysis in **Supplementary Figure S21B**). Three studies included ECOG performance status and identified it as significantly linked to OS (HR = 2.58, 95% CI: 1.43–4.63, *p* = 0.002; *I²* = 0.0%, **Supplementary Figure S22A**; sensitivity analysis in **Supplementary Figure S23A**). DS was examined in three studies, but pooled analysis failed to demonstrate a robust statistical connection (HR = 2.16, 95% CI: 0.10–45.29, *p* = 0.620; *I²* = 93.8%, **Supplementary Figure S22B**; sensitivity analysis in **Supplementary Figure S23B**).

Sensitivity analyses for all outcomes indicated the robustness and reliability of the findings.

#### 3.6.2 PFS

Two studies assessed SUVmax, with no statistically meaningful relationship with PFS (HR = 2.00, 95% CI: 0.47–8.62, *p* = 0.351; *I²* = 75.1%, **Supplementary Figure S24A**; sensitivity analysis in **Supplementary Figure S25A**). Preliminary analysis based on three studies indicated a statistically significant link involving MTV (HR = 1.04, 95% CI: 1.02–1.07, *p* = 0.001; *I²* = 0.0%, **Supplementary Figure S24B**). However, sensitivity analysis (**Supplementary Figure S25B**) raised concerns regarding result stability. After excluding the study by Leithner D (2024), the previously observed association was no longer significant (HR = 0.96, 95% CI: 0.28–3.20, *p* = 0.941; *I²* = 0.0%, **Supplementary Figure S26A**; sensitivity analysis in **Supplementary Figure S27A**). TLG, included in two studies, showed a trend toward significance but fell short of the required threshold (HR = 3.68, 95% CI: 0.95–14.28, *p* = 0.060; *I²* = 34.8%, **Supplementary Figure S26B**; sensitivity analysis in **Supplementary Figure S27B**).

In two studies, LDH was not significantly linked to PFS (HR = 0.94, 95% CI: 0.19–4.70, *p* = 0.942; *I²* = 74.5%, **Supplementary Figure S28A**; sensitivity analysis in **Supplementary Figure S29A**). DS, reported in two studies, also lacked a significant correlation (HR = 2.66, 95% CI: 0.18–39.41, *p* = 0.476; *I²* = 89.3%, **Supplementary Figure S28B**; sensitivity analysis in **Supplementary Figure S29B**). IPI, assessed in two studies, showed a statistically significant association with PFS (HR = 3.07, 95% CI: 1.59–5.93, *p* = 0.001; *I²* = 0.0%, **Supplementary Figure S30**; sensitivity analysis in **Supplementary Figure S31**).

Sensitivity analyses for all outcomes indicated the robustness and reliability of the findings.

## 4 Discussion

### 4.1 Summary of meta-analysis results

This meta-analysis represents a comprehensive synthesis of existing literature evaluating how ^18^F-FDG PET/CT-derived metabolic metrics and clinical indicators predict outcomes in CAR-T-treated DLBCL patients. Fourteen studies were selected for the combined analysis. Univariate results demonstrated that elevated SUVmax, MTV, TLG, LDH, ECOG performance status, DS, and IPI were significantly linked to poorer OS. Conversely, parameters such as TMTV and IMPI showed no statistically significant correlation with survival outcomes. Additionally, high SUVmax, MTV, TMTV, TLG, LDH, and ECOG were associated with poorer PFS, while parameters such as ΔSUVmax, ΔMTV, ΔTLG, DS, IPI, and IMPI did not significantly predict survival outcomes. In multivariate models, elevated LDH and ECOG remained significant predictors for OS, while IPI emerged as a key predictor of PFS.

In this meta-analysis, several factors may explain the discrepancies between univariate and multivariate analysis outcomes. First, multicollinearity between variables: Univariate analysis assesses the relationship of each variable with prognosis independently, potentially overlooking inter-variable correlations (e.g., between SUVmax and MTV). In contrast, multivariate analysis considers these relationships, where parameters like IPI encompass LDH and ECOG, leading to collinearity and potential replacement of LDH and ECOG by IPI, causing their loss of significance in prediction. Second, sample size impacts: Insufficient sample sizes may prevent the retention of statistical significance for certain parameters, as observed with IMPI, thereby limiting its predictive value. Third, variability in imaging segmentation methods: Differences in the segmentation approaches for SUVmax, MTV, and TLG can reduce the stability of results in multivariate analysis, impeding reliable prognostic predictions. Fourth, biological heterogeneity: Metabolic parameters can be influenced by tumor molecular subtypes or genetic mutations, whereas clinical indicators such as LDH and ECOG may more consistently reflect patient status across populations. Finally, the limitations of dynamic metabolic indicators (such as ΔSUVmax, ΔMTV, and ΔTLG) should be noted. These values often span the entire treatment duration and may be affected by multiple confounding variables, limiting their relevance compared to interim PET/CT assessments, which are more commonly used in DLBCL prognosis (41).

In univariate analyses, SUVmax was significantly linked to OS and PFS among the PET parameters, supporting the results of Gui et al. (22). However, conflicting reports remain in the literature regarding its independent prognostic value in DLBCL (7, 42). Cui et al. (43) reported a lack of significant relationship between SUVmax and survival outcomes. A key limitation of SUVmax is its focus on the most metabolically active voxel, which may not capture the full extent of disease burden (44, 45). Moreover, SUVmax values may be affected by variability in imaging procedures, scanner calibration, and the interval between radiotracer administration and image capture. With respect to CAR-T therapy, which may trigger immune-related inflammation or tumor necrosis, post-treatment SUVmax readings can be further confounded, diminishing their predictive accuracy (46). Research by Vedvyas Y et al. (47) demonstrated that PET enables dynamic observation of the biphasic “expansion-contraction” kinetics of CAR-T cells at tumor sites in mouse models. Additionally, Fröse J et al. (48) developed an antigen-based imaging approach for dynamic systemic monitoring of CAR-T cells, identifying CAR-PET signals in the spleen as a predictive marker for early mortality risk. Dynamic PET imaging refers to the real-time, quantitative monitoring of CAR-T cell biodistribution, tumor retention, and treatment response via continuous or multiple time-point PET scans, serving as an indispensable tool for optimizing CAR-T therapy in tumors. Thus (49–51), dynamic whole-body PET imaging represents an emerging and rapidly advancing frontier technology; however, its systematic application in CAR-T therapy, particularly in routine clinical practice, remains underdeveloped. With ongoing advancements in science and technology, dynamic PET is expected to achieve clinical application in CAR-T cancer therapy, enabling improved real-time monitoring of CAR-T cells and providing valuable insights for patients receiving CAR-T therapy.

By integrating both tumor volume and metabolic activity, MTV and TLG deliver a more comprehensive appraisal of the tumor load. This theoretical advantage was supported by the univariate analyses, where significant correlations between these two markers and unfavorable OS and PFS were observed, corroborating findings from earlier research (52–54). Although the precise biological mechanisms underpinning high MTV remain unclear, increased concentrations of inflammatory cytokines, including IL-6, IL-15, and TNF-α, have been detected in DLBCL individuals and may influence tumor metabolism through modulation of the tumor microenvironment (55). Furthermore, TLG levels have been found to correlate strongly with IL-6, IFN-γ, and ferritin levels (56), all of which are critical mediators in cancer development, progression, and elimination (57). These observations suggest that PET markers including MTV and TLG may have prognostic importance due to the dynamic interplay between metabolism and immune responses. However, the mechanistic basis for these associations remains to be fully elucidated and warrants further investigation.

Clinical prognostic indicators—particularly IPI, LDH, and ECOG—also demonstrated significant value in predicting outcomes among DLBCL patients receiving CAR-T therapy. The IPI remains a standard prognostic tool in clinical practice since its introduction in 1993 (58), incorporating age, Ann Arbor stage, LDH level, ECOG score, and extranodal involvement. Among them, elevated serum LDH levels have the most substantial prognostic impact (59). LDH, an enzyme present in most tissues, is released when tumor cells proliferate, invade, and metastasize, causing damage to surrounding normal tissues and leading to elevated serum LDH levels. Previous studies (59) have shown that elevated LDH levels offer the highest prognostic value in DLBCL. Moreover, Keane et al. (60) highlighted the association between LDH levels and high tumor burden as well as the tumor immune microenvironment. This suggests that both high tumor burden and the immune microenvironment may influence LDH as a prognostic marker in DLBCL. This insight directs attention toward focusing on tumor burden and immune microenvironment factors to potentially enhance prognostic prediction for DLBCL patients. ECOG performance status, a measure of functional impairment, was also significantly associated with both OS and PFS (61). Higher ECOG scores have been linked to increased risks of immune-related adverse events during treatment (62), potentially compromising therapy outcomes. Moreover, there is emerging evidence that checkpoint inhibitors like PD-1 and PD-L1 molecules in immune modulation are linked to both ECOG status and disease severity (63–65). Understanding the immune landscape and its interaction with physical performance status may thus offer novel insights for prognostic modeling and treatment optimization.

In 2009, the Deauville score was introduced as a standardized method for evaluating treatment response in lymphoma (66, 67). In DLBCL, a high Deauville score after treatment typically correlates with a poorer prognosis, characterized by shorter OS and PFS (68). Previous studies have identified several factors influencing DLBCL prognosis, including the tumor proliferation index, as measured by the proliferating cell nuclear antigen Ki67 (69, 70), P53 overexpression (71, 72), and protein expression levels of the MYC oncogene and BCL-2 (73). However, no research has established a direct relationship between the Deauville score and these markers, such as Ki67, P53, MYC, and BCL-2 expression. Further investigation into these associations could provide valuable insights into improving prognostic predictions for DLBCL.

Traditional PET parameters primarily reflect tumor volume and metabolic intensity but provide insufficient characterization of intratumoral spatial heterogeneity, spatiotemporal patterns of metabolism, and interactions with the immune microenvironment. Therefore, next-generation PET-based prognostic biomarkers are required to address these limitations. Next-generation PET-based prognostic biomarkers, including radiomics and texture analysis (74, 75), artificial intelligence and deep learning (76), and novel radiotracers and multiparametric imaging (77), are shifting prognostic assessment from the tumor burden era toward the tumor biology era. Radiomics and texture analysis can reveal intratumoral heterogeneity, predict toxicity risks, and facilitate the construction of comprehensive predictive models (78). Artificial intelligence and deep learning can minimize subjectivity in manual segmentation and feature extraction, uncover patterns beyond human recognition, and integrate information across multiple time points (79). Novel radiotracers and multiparametric imaging can visualize the tumor immune microenvironment and elucidate mechanisms of immune evasion. Next-generation PET-based prognostic biomarkers will be a focal point of future research. Standardization, prospective validation of these novel markers, and development of new radiotracers will enhance the therapeutic value of CAR-T therapy for DLBCL.

Taken together, this meta-analysis demonstrates that prognostic prediction in CAR-T-treated DLBCL patients can be informed by ^18^F-FDG PET/CT and recognized clinical markers.

### 4.2 Clinical implications

The ^18^F-FDG PET/CT technique, which combines metabolic imaging via positron emission tomography with anatomical visualization through computed tomography, offers a powerful tool for detecting active tumor regions. In clinical practice, this imaging modality has become a cornerstone in managing DLBCL. Its utility spans the full spectrum of patient care—from initial diagnosis and disease staging to evaluating treatment response, estimating prognosis, identifying recurrence, and guiding personalized therapeutic interventions.

### 4.3 Study limitations

Although this analysis offers valuable insights, it is important to recognize certain limitations. First, a predominance of retrospective studies was included, with just a prospective study and an absence of randomized controlled trials, potentially introducing selection bias and limiting causal inference. Second, there was variability in the cutoff thresholds used to define imaging and clinical parameters across studies, which may have introduced heterogeneity and influenced effect estimates. Third, considerable heterogeneity was observed across the studies. The possible sources of heterogeneity may include differences in PET acquisition protocols, segmentation methods, CAR-T infusion timing, and biological phenomena such as immune-related inflammation. Therefore, a random-effects model was employed for analysis. Relatively few studies were eligible for the specific endpoints. Due to the limited number of publications, a stable assessment of publication bias was not feasible, and thus no publication bias test was conducted. Furthermore, due to insufficient stratified data, subgroup analyses could not be performed, thereby limiting exploration of potential effect modifiers. This study covered multiple time points and investigated FDG PET parameters across different disease conditions, which substantially limited its generalizability. Thus, these results should be analyzed with a careful perspective.

### 4.4 Outlook and implications

Although ^18^F-FDG PET/CT is instrumental in improving therapeutic strategies for DLBCL, its widespread application remains constrained by cost considerations and limited availability in certain healthcare settings. Future cost-effectiveness analyses are warranted to support its broader integration into routine care. In addition, integrating PET/CT with other imaging modalities, imaging biomarkers, and emerging frameworks in systems biology and immuno-oncology can enable more precise treatment personalization. Importantly, large prospective multicenter studies are essential to confirm the predictive significance of PET/CT here. Such efforts may pave the way for standardized imaging-based risk stratification models that guide clinical decisions and promote better patient outcomes and well-being.

## 5 Conclusion

In conclusion, this meta-analysis underscored the predictive significance of both ^18^F-FDG PET/CT-derived metabolic imaging metrics and conventional clinical markers in CAR-T-treated DLBCL patients. Integrating these modalities may greatly enhance risk assessment and support tailored therapeutic decisions in this cohort.

## Data Availability

The original contributions presented in the study are included in the article/**Supplementary Material**. Further inquiries can be directed to the corresponding author.

## Supplementary material

Supplementary Table 1 PRISMA protocol.

Supplementary Table 2 Literature search strategy.

Supplementary Figure 1 PRISMA flow diagram

Supplementary Figure 2 Sensitivity analysis of the prognostic effect of SUVmax **(A)** and MTV **(B)** on overall survival.

Supplementary Figure 3 Sensitivity analysis of the prognostic effect of TMTV **(A)** and TLG **(B)** on overall survival.

Supplementary Figure 4 Sensitivity analysis of the prognostic effect of LDH **(A)** and ECOG **(B)** on overall survival.

Supplementary Figure 5 Sensitivity analysis of the prognostic effect of DS **(A)** and IPI **(B)** on overall survival.

Supplementary Figure 6 Sensitivity analysis of the prognostic effect of IMPI **(A)** on overall survival.

Supplementary Figure 7 Sensitivity analysis of the prognostic effect of SUVmax **(A)** and ΔSUVmax **(B)** on progression-free survival.

Supplementary Figure 8 Forest plot of the prognostic effect of MTV **(A)** and ΔMTV **(B)** on progression-free survival.

Supplementary Figure 9 Sensitivity analysis of the meta-analysis on the prognostic effect of MTV **(A)** and ΔMTV **(B)** on progression-free survival.

Supplementary Figure 10 Forest plot of the meta-analysis on the prognostic effect of TMTV **(A)** and TLG **(B)** on progression-free survival.

Supplementary Figure 11 Sensitivity analysis of the meta-analysis on the prognostic effect of TMTV **(A)** and TLG **(B)** on progression-free survival.

Supplementary Figure 12 Forest plot of the prognostic effect of ΔTLG **(A)** and LDH **(B)** on progression-free survival.

Supplementary Figure 13 Sensitivity analysis of the meta-analysis on the prognostic effect of ΔTLG **(A)** and LDH **(B)** on progression-free survival.

Supplementary Figure 14 Forest plot of the meta-analysis on the prognostic effect of ECOG **(A)** and DS **(B)** on progression-free survival.

Supplementary Figure 15 Sensitivity analysis of the meta-analysis on the prognostic effect of ECOG **(A)** and DS **(B)** on progression-free survival.

Supplementary Figure 16 Forest plot of the prognostic effect of IPI **(A)** and IMPI **(B)** on progression-free survival.

Supplementary Figure 17 Sensitivity analysis of the meta-analysis on the prognostic effect of IPI **(A)** and IMPI **(B)** on progression-free survival.

Supplementary Figure 18 Forest plot of the prognostic effect of SUVmax **(A)** and MTV **(B)** on overall survival.

Supplementary Figure 19 Sensitivity analysis of the prognostic effect of SUVmax **(A)** and MTV **(B)** on overall survival.

Supplementary Figure 20 Forest plot of the meta-analysis on the prognostic effect of TMTV **(A)** and LDH **(B)** on overall survival.

Supplementary Figure 21 Sensitivity analysis of the prognostic effect of TMTV **(A)** and LDH **(B)** on overall survival.

Supplementary Figure 22 Forest plot of the meta-analysis on the prognostic effect of ECOG **(A)** and DS **(B)** on overall survival.

Supplementary Figure 23 Sensitivity analysis of the prognostic effect of ECOG **(A)** and DS **(B)** on overall survival.

Supplementary Figure 24 Forest plot of the meta-analysis on the prognostic effect of SUVmax **(A)** and MTV **(B)** on progression-free survival.

Supplementary Figure 25 Sensitivity analysis of the prognostic effect of SUVmax **(A)** and MTV **(B)** on progression-free survival.

Supplementary Figure 26 Forest plot of the meta-analysis on the prognostic effect of MTV **(A)** and TLG **(B)** on progression-free survival.

Supplementary Figure 27 Sensitivity analysis of the prognostic effect of MTV **(A)** and TLG **(B)** on progression-free survival.

Supplementary Figure 28 Forest plot of the meta-analysis on the prognostic effect of LDH **(A)** and DS **(B)** on progression-free survival.

Supplementary Figure 29 Sensitivity analysis of the prognostic effect of LDH **(A)** and DS **(B)** on progression-free survival.

Supplementary Figure 30 Forest plot of the meta-analysis on the prognostic effect of IPI on progression-free survival.

Supplementary Figure 31 Sensitivity analysis of the prognostic effect of IPI **(A)** on progression-free survival.
