# Supplementary material for: Systematic review and meta−analysis of PET−based prognostic metrics in CAR−T treatment of DLBCL
Source: Front Immunol. 2026 Feb 24;17:1679181. doi: 10.3389/fimmu.2026.1679181 (PMC12971458; doi:10.3389/fimmu.2026.1679181)
Supplement: Supplementary Table 1 — PRISMA protocol. [file Table1.docx]

Supplementary Material

# **1 Supplementary Tables and Figures**

## **Supplementary Tables**

## **Table S1** PRISMA protocol

| **Section and Topic** | **Item #** | **Checklist item** | **Location where item is reported** |
| --- | --- | --- | --- |
| **TITLE** | | |  |
| Title | 1 | Identify the report as a systematic review. | P1 |
| **ABSTRACT** | | |  |
| Abstract | 2 | See the PRISMA 2020 for Abstracts checklist. | P1-2 |
| **INTRODUCTION** | | |  |
| Rationale | 3 | Describe the rationale for the review in the context of existing knowledge. | P2-3 |
| Objectives | 4 | Provide an explicit statement of the objective(s) or question(s) the review addresses. | P2-3 |
| **METHODS** | | |  |
| Eligibility criteria | 5 | Specify the inclusion and exclusion criteria for the review and how studies were grouped for the syntheses. | P3-4 |
| Information sources | 6 | Specify all databases, registers, websites, organisations, reference lists and other sources searched or consulted to identify studies. Specify the date when each source was last searched or consulted. | P3-4 |
| Search strategy | 7 | Present the full search strategies for all databases, registers and websites, including any filters and limits used. | P3-4 |
| Selection process | 8 | Specify the methods used to decide whether a study met the inclusion criteria of the review, including how many reviewers screened each record and each report retrieved, whether they worked independently, and if applicable, details of automation tools used in the process. | P3-4 |
| Data collection process | 9 | Specify the methods used to collect data from reports, including how many reviewers collected data from each report, whether they worked independently, any processes for obtaining or confirming data from study investigators, and if applicable, details of automation tools used in the process. | P3-4 |
| Data items | 10a | List and define all outcomes for which data were sought. Specify whether all results that were compatible with each outcome domain in each study were sought (e.g. for all measures, time points, analyses), and if not, the methods used to decide which results to collect. | P3-4 |
|  | 10b | List and define all other variables for which data were sought (e.g. participant and intervention characteristics, funding sources). Describe any assumptions made about any missing or unclear information. | P3-4 |
| Study risk of bias assessment | 11 | Specify the methods used to assess risk of bias in the included studies, including details of the tool(s) used, how many reviewers assessed each study and whether they worked independently, and if applicable, details of automation tools used in the process. | P3-4 |
| Effect measures | 12 | Specify for each outcome the effect measure(s) (e.g. risk ratio, mean difference) used in the synthesis or presentation of results. | P3-4 |
| Synthesis methods | 13a | Describe the processes used to decide which studies were eligible for each synthesis (e.g. tabulating the study intervention characteristics and comparing against the planned groups for each synthesis (item #5)). | P3-4 |
|  | 13b | Describe any methods required to prepare the data for presentation or synthesis, such as handling of missing summary statistics, or data conversions. | P3-4 |
|  | 13c | Describe any methods used to tabulate or visually display results of individual studies and syntheses. | P3-4 |
|  | 13d | Describe any methods used to synthesize results and provide a rationale for the choice(s). If meta-analysis was performed, describe the model(s), method(s) to identify the presence and extent of statistical heterogeneity, and software package(s) used. | P3-4 |
|  | 13e | Describe any methods used to explore possible causes of heterogeneity among study results (e.g. subgroup analysis, meta-regression). | P3-4 |
|  | 13f | Describe any sensitivity analyses conducted to assess robustness of the synthesized results. | P3-4 |
| Reporting bias assessment | 14 | Describe any methods used to assess risk of bias due to missing results in a synthesis (arising from reporting biases). | P3-4 |
| Certainty assessment | 15 | Describe any methods used to assess certainty (or confidence) in the body of evidence for an outcome. | P3-4 |
| **RESULTS** | | |  |
| Study selection | 16a | Describe the results of the search and selection process, from the number of records identified in the search to the number of studies included in the review, ideally using a flow diagram. | P4-7 |
|  | 16b | Cite studies that might appear to meet the inclusion criteria, but which were excluded, and explain why they were excluded. | P4-7 |
| Study characteristics | 17 | Cite each included study and present its characteristics. | P4-7 |
| Risk of bias in studies | 18 | Present assessments of risk of bias for each included study. | P4-7 |
| Results of individual studies | 19 | For all outcomes, present, for each study: (a) summary statistics for each group (where appropriate) and (b) an effect estimate and its precision (e.g. confidence/credible interval), ideally using structured tables or plots. | P4-7 |
| Results of syntheses | 20a | For each synthesis, briefly summarise the characteristics and risk of bias among contributing studies. | P4-7 |
|  | 20b | Present results of all statistical syntheses conducted. If meta-analysis was done, present for each the summary estimate and its precision (e.g. confidence/credible interval) and measures of statistical heterogeneity. If comparing groups, describe the direction of the effect. | P4-7 |
|  | 20c | Present results of all investigations of possible causes of heterogeneity among study results. | P4-7 |
|  | 20d | Present results of all sensitivity analyses conducted to assess the robustness of the synthesized results. | P4-7 |
| Reporting biases | 21 | Present assessments of risk of bias due to missing results (arising from reporting biases) for each synthesis assessed. | P4-7 |
| Certainty of evidence | 22 | Present assessments of certainty (or confidence) in the body of evidence for each outcome assessed. | P4-7 |
| **DISCUSSION** | | |  |
| Discussion | 23a | Provide a general interpretation of the results in the context of other evidence. | P7-11 |
|  | 23b | Discuss any limitations of the evidence included in the review. | P7-11 |
|  | 23c | Discuss any limitations of the review processes used. | P7-11 |
|  | 23d | Discuss implications of the results for practice, policy, and future research. | P7-11 |
| **OTHER INFORMATION** | | |  |
| Registration and protocol | 24a | Provide registration information for the review, including register name and registration number, or state that the review was not registered. | P3 |
|  | 24b | Indicate where the review protocol can be accessed, or state that a protocol was not prepared. | P3 |
|  | 24c | Describe and explain any amendments to information provided at registration or in the protocol. | P3 |
| Support | 25 | Describe sources of financial or non-financial support for the review, and the role of the funders or sponsors in the review. | P12 |
| Competing interests | 26 | Declare any competing interests of review authors. | P11 |
| Availability of data, code and other materials | 27 | Report which of the following are publicly available and where they can be found: template data collection forms; data extracted from included studies; data used for all analyses; analytic code; any other materials used in the review. | P21 |

*From:*  Page MJ, McKenzie JE, Bossuyt PM, Boutron I, Hoffmann TC, Mulrow CD, et al. The PRISMA 2020 statement: an updated guideline for reporting systematic reviews. BMJ 2021;372:n71. doi: 10.1136/bmj.n71

**Table S2** Literature search strategy

| **Pubmed** | | |
| --- | --- | --- |
| **#** | **Query** | **Results** |
| **1** | Lymphoma, Large B-Cell, Diffuse[MeSH Terms] | 25099 |
| **2** | ‘Diffuse Histiocytic Lymphoma’[Title/Abstract] OR ‘Diffuse Histiocytic Lymphomas’[Title/Abstract] OR ‘Diffuse Large B Cell Lymphoma’[Title/Abstract] OR ‘diffuse large cell B cell lymphoma’[Title/Abstract] OR ‘Diffuse Large Cell Lymphoma’[Title/Abstract] OR ‘diffuse large cell non Hodgkin lymphoma’[Title/Abstract] OR ‘diffuse large cell non Hodgkin`s lymphoma’[Title/Abstract] OR ‘Diffuse Large Cell Lymphomas’[Title/Abstract] OR ‘Diffuse, Large B Cell, Lymphoma’[Title/Abstract] OR ‘DLBCL’[Title/Abstract] OR ‘Histiocytic Lymphoma’[Title/Abstract] OR ‘Histiocytic Lymphoma, Diffuse’[Title/Abstract] OR ‘Histiocytic Lymphomas’[Title/Abstract] OR ‘large B cell diffuse lymphoma’[Title/Abstract] OR ‘large B cell lymphoma’[Title/Abstract] OR ‘large B cell lymphomas’[Title/Abstract] OR ‘large B cell malignant lymphoma’[Title/Abstract] OR ‘large B cell non Hodgkin lymphoma’[Title/Abstract] OR ‘large B cell non Hodgkin`s lymphoma’[Title/Abstract] OR ‘Large Cell Lymphoma, Diffuse’[Title/Abstract] OR ‘Large Lymphoid Lymphoma, Diffuse’[Title/Abstract] OR ‘Large-Cell Lymphoma, Diffuse’[Title/Abstract] OR ‘Lymphoma, Diffuse Histiocytic’[Title/Abstract] OR ‘Lymphoma, Diffuse Large Cell’[Title/Abstract] OR ‘Lymphoma, Histiocytic’[Title/Abstract] OR ‘Lymphoma, Histiocytic, Diffuse’[Title/Abstract] OR ‘lymphoma, large B-cell, diffuse’[Title/Abstract] OR ‘Lymphoma, Large Cell, Diffuse’[Title/Abstract] OR ‘Lymphoma, Large Lymphoid, Diffuse’[Title/Abstract] | 25880 |
| **3** | ‘CAR engineered T cell immunotherapy’[Title/Abstract] OR ‘CAR engineered T cell therapy’[Title/Abstract] OR ‘CAR modified T cell immunotherapy’[Title/Abstract] OR (‘CAR modified T cell therapy’[Title/Abstract] OR ‘CAR T Cell Therapy’[Title/Abstract] OR ‘CAR T Cell Therapies’[Title/Abstract] OR ‘chimeric antigen receptor engineered T cell immunotherapy’[Title/Abstract] OR ‘chimeric antigen receptor engineered T cell therapy’[Title/Abstract] OR ‘chimeric antigen receptor modified T cell immunotherapy’[Title/Abstract] OR ‘chimeric antigen receptor modified T cell therapy’[Title/Abstract] OR ‘chimeric antigen receptor T cell cancer immunotherapy’[Title/Abstract] OR ‘chimeric antigen receptor T cell cancer therapy’[Title/Abstract] OR ‘chimeric antigen receptor T cell immunotherapy’[Title/Abstract] OR ‘chimeric antigen receptor T cell therapy’[Title/Abstract] OR ‘Chimeric Antigen Receptor Therapy’[Title/Abstract] OR ‘T-Cell Therapies, CAR’[Title/Abstract] OR ‘T-Cell Therapy, CAR’[Title/Abstract] OR ‘Therapies, CAR T Cell’[Title/Abstract] OR ‘Therapy, CAR T Cell’[Title/Abstract] | 7201 |
| **4** | positron emission tomography-computed tomography[MeSH Terms] | 22671 |
| **5** | ‘computer assisted positron emission tomography’[Title/Abstract] OR ‘CT Scan,PET’[Title/Abstract] OR ‘CT Scans,PET’[Title/Abstract] OR ‘CT PET’[Title/Abstract] OR ‘CT PET Scan’[Title/Abstract] OR ‘CT PET Scans’[Title/Abstract] OR ‘PET CT Scan’[Title/Abstract] OR ‘PET Scan, CT’[Title/Abstract] OR ‘PET Scans, CT’[Title/Abstract] OR ‘PET CT’[Title/Abstract] OR ‘PET CT Scans’[Title/Abstract] OR ‘positron emission computed tomography’[Title/Abstract] OR ‘positron emission tomography computed tomography’[Title/Abstract] OR ‘positron emission tomography, computer assisted’[Title/Abstract] OR ‘positron-emission tomography[Title/Abstract] AND computed tomography’[Title/Abstract] OR ‘Scan, CT PET’[Title/Abstract] OR ‘Scan, PET CT’[Title/Abstract] OR ‘Scans, CT PET’[Title/Abstract] OR ‘Scans, PET CT‘[Title/Abstract] | 30847 |
| **6** | (#1 OR #2) AND #3 AND (#4 OR #5) | **43** |

| **Embase** | | |
| --- | --- | --- |
| **#** | **Query** | **Results** |
| **1** | 'diffuse large b cell lymphoma'/exp | 33656 |
| **2** | 'diffuse histiocytic lymphoma':ti,ab,kw OR 'diffuse histiocytic lymphomas':ti,ab,kw OR 'diffuse large b cell lymphoma':ti,ab,kw OR 'diffuse large cell b cell lymphoma':ti,ab,kw OR 'diffuse large cell lymphoma':ti,ab,kw OR 'diffuse large cell non hodgkin lymphoma':ti,ab,kw OR 'diffuse large cell non hodgkin`s lymphoma':ti,ab,kw OR 'diffuse large cell lymphomas':ti,ab,kw OR 'diffuse, large b cell, lymphoma':ti,ab,kw OR 'dlbcl':ti,ab,kw OR 'histiocytic lymphoma':ti,ab,kw OR 'histiocytic lymphoma, diffuse':ti,ab,kw OR 'histiocytic lymphomas':ti,ab,kw OR 'large b cell diffuse lymphoma':ti,ab,kw OR 'large b cell lymphoma':ti,ab,kw OR 'large b cell lymphomas':ti,ab,kw OR 'large b cell malignant lymphoma':ti,ab,kw OR 'large b cell non hodgkin lymphoma':ti,ab,kw OR 'large b cell non hodgkin`s lymphoma':ti,ab,kw OR 'large cell lymphoma, diffuse':ti,ab,kw OR 'large lymphoid lymphoma, diffuse':ti,ab,kw OR 'large-cell lymphoma, diffuse':ti,ab,kw OR 'lymphoma, diffuse histiocytic':ti,ab,kw OR 'lymphoma, diffuse large cell':ti,ab,kw OR 'lymphoma, histiocytic':ti,ab,kw OR 'lymphoma, histiocytic, diffuse':ti,ab,kw OR 'lymphoma, large b-cell, diffuse':ti,ab,kw OR 'lymphoma, large cell, diffuse':ti,ab,kw OR 'lymphoma, large lymphoid, diffuse':ti,ab,kw | 49714 |
| **3** | 'car engineered t cell immunotherapy':ti,ab,kw OR 'car engineered t cell therapy':ti,ab,kw OR 'car modified t cell immunotherapy':ti,ab,kw OR 'car modified t cell therapy':ti,ab,kw OR 'car t cell therapy':ti,ab,kw OR 'car t cell therapies':ti,ab,kw OR 'chimeric antigen receptor engineered t cell immunotherapy':ti,ab,kw OR 'chimeric antigen receptor engineered t cell therapy':ti,ab,kw OR 'chimeric antigen receptor modified t cell immunotherapy':ti,ab,kw OR 'chimeric antigen receptor modified t cell therapy':ti,ab,kw OR 'chimeric antigen receptor t cell cancer immunotherapy':ti,ab,kw OR 'chimeric antigen receptor t cell cancer therapy':ti,ab,kw OR 'chimeric antigen receptor t cell immunotherapy':ti,ab,kw OR 'chimeric antigen receptor t cell therapy':ti,ab,kw OR 'chimeric antigen receptor therapy':ti,ab,kw OR 't-cell therapies, car':ti,ab,kw OR 't-cell therapy, car':ti,ab,kw OR 'therapies, car t cell':ti,ab,kw OR 'therapy, car t cell':ti,ab,kw | 13762 |
| **4** | 'positron emission tomography-computed tomography'/exp | 79434 |
| **5** | 'computer assisted positron emission tomography':ti,ab,kw OR 'ct scan,pet':ti,ab,kw OR 'ct scans,pet':ti,ab,kw OR 'ct pet':ti,ab,kw OR 'ct pet scan':ti,ab,kw OR 'ct pet scans':ti,ab,kw OR 'pet ct scan':ti,ab,kw OR 'pet scan, ct':ti,ab,kw OR 'pet scans, ct':ti,ab,kw OR 'pet ct':ti,ab,kw OR 'pet ct scans':ti,ab,kw OR 'positron emission computed tomography':ti,ab,kw OR 'positron emission tomography computed tomography':ti,ab,kw OR 'positron emission tomography, computer assisted':ti,ab,kw OR 'positron-emission tomography and computed tomography':ti,ab,kw OR 'scan, ct pet':ti,ab,kw OR 'scan, pet ct':ti,ab,kw OR 'scans, ct pet':ti,ab,kw OR 'scans, pet ct':ti,ab,kw | 96555 |
| **6** | #1 OR #2 | 57791 |
| **7** | #4 OR #5 | 124005 |
| **8** | #3 AND #6 AND #7 | **312** |

| **Cochrane Library** | | |
| --- | --- | --- |
| **#** | **Query** | **Results** |
| **1** | MeSH descriptor: [Lymphoma, Large B-Cell, Diffuse] explode all trees | 698 |
| **2** | (‘Diffuse Histiocytic Lymphoma’ OR ‘Diffuse Histiocytic Lymphomas’ OR ‘Diffuse Large B Cell Lymphoma’ OR ‘diffuse large cell B cell lymphoma’ OR ‘Diffuse Large Cell Lymphoma’ OR ‘diffuse large cell non Hodgkin lymphoma’ OR ‘diffuse large cell non Hodgkin`s lymphoma’ OR ‘Diffuse Large Cell Lymphomas’ OR ‘Diffuse, Large B Cell, Lymphoma’ OR ‘DLBCL’ OR ‘Histiocytic Lymphoma’ OR ‘Histiocytic Lymphoma, Diffuse’ OR ‘Histiocytic Lymphomas’ OR ‘large B cell diffuse lymphoma’ OR ‘large B cell lymphoma’ OR ‘large B cell lymphomas’ OR ‘large B cell malignant lymphoma’ OR ‘large B cell non Hodgkin lymphoma’ OR ‘large B cell non Hodgkin`s lymphoma’ OR ‘Large Cell Lymphoma, Diffuse’ OR ‘Large Lymphoid Lymphoma, Diffuse’ OR ‘Large-Cell Lymphoma, Diffuse’ OR ‘Lymphoma, Diffuse Histiocytic’ OR ‘Lymphoma, Diffuse Large Cell’ OR ‘Lymphoma, Histiocytic’ OR ‘Lymphoma, Histiocytic, Diffuse’ OR ‘lymphoma, large B-cell, diffuse’ OR ‘Lymphoma, Large Cell, Diffuse’ OR ‘Lymphoma, Large Lymphoid, Diffuse’):ti,ab,kw | 2549 |
| **3** | (‘CAR engineered T cell immunotherapy’ OR ‘CAR engineered T cell therapy’ OR ‘CAR modified T cell immunotherapy’ OR ‘CAR modified T cell therapy’ OR ‘CAR T Cell Therapy’ OR ‘CAR T Cell Therapies’ OR ‘chimeric antigen receptor engineered T cell immunotherapy’ OR ‘chimeric antigen receptor engineered T cell therapy’ OR ‘chimeric antigen receptor modified T cell immunotherapy’ OR ‘chimeric antigen receptor modified T cell therapy’ OR ‘chimeric antigen receptor T cell cancer immunotherapy’ OR ‘chimeric antigen receptor T cell cancer therapy’ OR ‘chimeric antigen receptor T cell immunotherapy’ OR ‘chimeric antigen receptor T cell therapy’ OR ‘Chimeric Antigen Receptor Therapy’ OR ‘T-Cell Therapies, CAR’ OR ‘T-Cell Therapy, CAR’ OR ‘Therapies, CAR T Cell’ OR ‘Therapy, CAR T Cell’):ti,ab,kw | 518 |
| **4** | MeSH descriptor: [Positron Emission Tomography Computed Tomography] explode all trees | 471 |
| **5** | (‘computer assisted positron emission tomography’ OR ‘CT Scan,PET’ OR ‘CT Scans,PET’ OR ‘CT PET’ OR ‘CT PET Scan’ OR ‘CT PET Scans’ OR ‘PET CT Scan’ OR ‘PET Scan, CT’ OR ‘PET Scans, CT’ OR ‘PET CT’ OR ‘PET CT Scans’ OR ‘positron emission computed tomography’ OR ‘positron emission tomography computed tomography’ OR ‘positron emission tomography, computer assisted’ OR ‘positron-emission tomography and computed tomography’ OR ‘Scan, CT PET’ OR ‘Scan, PET CT’ OR ‘Scans, CT PET’ OR ‘Scans, PET CT‘):ti,ab,kw | 5189 |
| **6** | (#1 OR #2) AND #3 AND (#4 OR #5) | **25** |

| **Web of Science** | | |
| --- | --- | --- |
| **#** | **Query** | **Results** |
| **1** | TS=((“Diffuse Histiocytic Lymphoma”) OR (“Diffuse Histiocytic Lymphomas”) OR (“Diffuse Large B Cell Lymphoma”) OR (“diffuse large cell B cell lymphoma”) OR (“Diffuse Large Cell Lymphoma”) OR (“diffuse large cell non Hodgkin lymphoma”) OR (“diffuse large cell non Hodgkin`s lymphoma”) OR (“Diffuse Large Cell Lymphomas”) OR (“Diffuse, Large B Cell, Lymphoma”) OR (“DLBCL”) OR (“Histiocytic Lymphoma”) OR (“Histiocytic Lymphoma, Diffuse”) OR (“Histiocytic Lymphomas”) OR (“large B cell diffuse lymphoma”) OR (“large B cell lymphoma”) OR (“large B cell lymphomas”) OR (“large B cell malignant lymphoma”) OR (“large B cell non Hodgkin lymphoma”) OR (“large B cell non Hodgkin`s lymphoma”) OR (“Large Cell Lymphoma, Diffuse”) OR (“Large Lymphoid Lymphoma, Diffuse”) OR (“Large-Cell Lymphoma, Diffuse”) OR (“Lymphoma, Diffuse Histiocytic”) OR (“Lymphoma, Diffuse Large Cell”) OR (“Lymphoma, Histiocytic”) OR (“Lymphoma, Histiocytic, Diffuse”) OR (“lymphoma, large B-cell, diffuse”) OR (“Lymphoma, Large Cell, Diffuse”) OR (“Lymphoma, Large Lymphoid, Diffuse”)) | 32161 |
| **2** | TS=((“CAR engineered T cell immunotherapy”) OR (“CAR engineered T cell therapy”) OR (“CAR modified T cell immunotherapy”) OR (“CAR modified T cell therapy”) OR (“CAR T Cell Therapy”) OR (“CAR T Cell Therapies”) OR (“chimeric antigen receptor engineered T cell immunotherapy”) OR (“chimeric antigen receptor engineered T cell therapy”) OR (“chimeric antigen receptor modified T cell immunotherapy”) OR (“chimeric antigen receptor modified T cell therapy”) OR (“chimeric antigen receptor T cell cancer immunotherapy”) OR (“chimeric antigen receptor T cell cancer therapy”) OR (“chimeric antigen receptor T cell immunotherapy”) OR (“chimeric antigen receptor T cell therapy”) OR (“Chimeric Antigen Receptor Therapy”) OR (“T-Cell Therapies, CAR”) OR (“T-Cell Therapy, CAR”) OR (“Therapies, CAR T Cell”) OR (“Therapy, CAR T Cell”)) | 8584 |
| **3** | TS=((“computer assisted positron emission tomography”) OR (“CT Scan,PET”) OR (“CT Scans,PET”) OR (“CT PET”) OR (“CT PET Scan”) OR (“CT PET Scans”) OR (“PET CT Scan”) OR (“PET Scan, CT”) OR (“PET Scans, CT”) OR (“PET CT”) OR (“PET CT Scans”) OR (“positron emission computed tomography”) OR (“positron emission tomography computed tomography”) OR (“positron emission tomography, computer assisted”) OR (“positron-emission tomography and computed tomography”) OR (“Scan, CT PET”) OR (“Scan, PET CT”) OR (“Scans, CT PET”) OR (“Scans, PET CT”)) | 69613 |
| **4** | #1 AND #2 AND #3 | **52** |

## **1.2 Supplementary Figures**

**
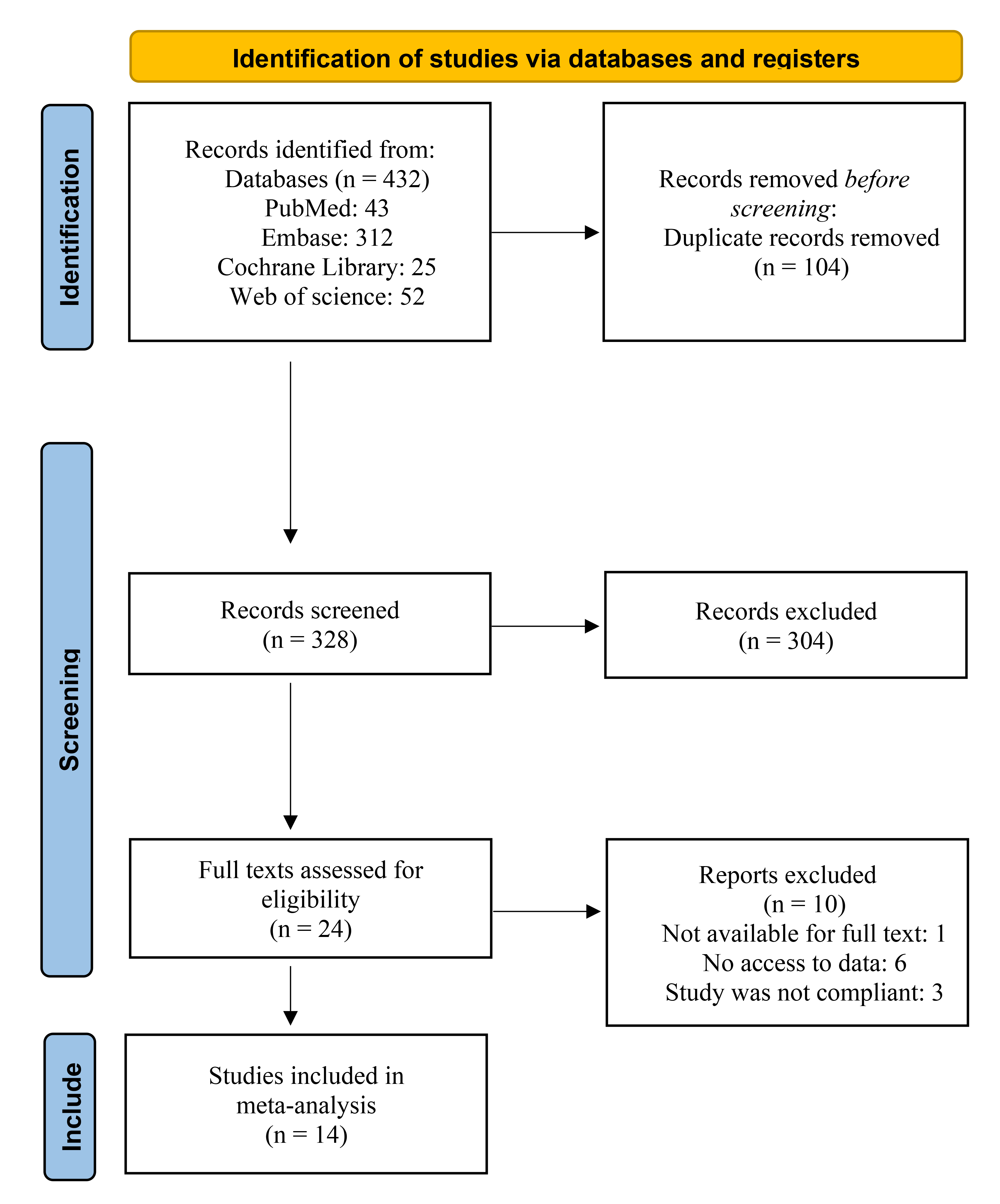
**

**Figure S1** PRISMA flow diagram

**
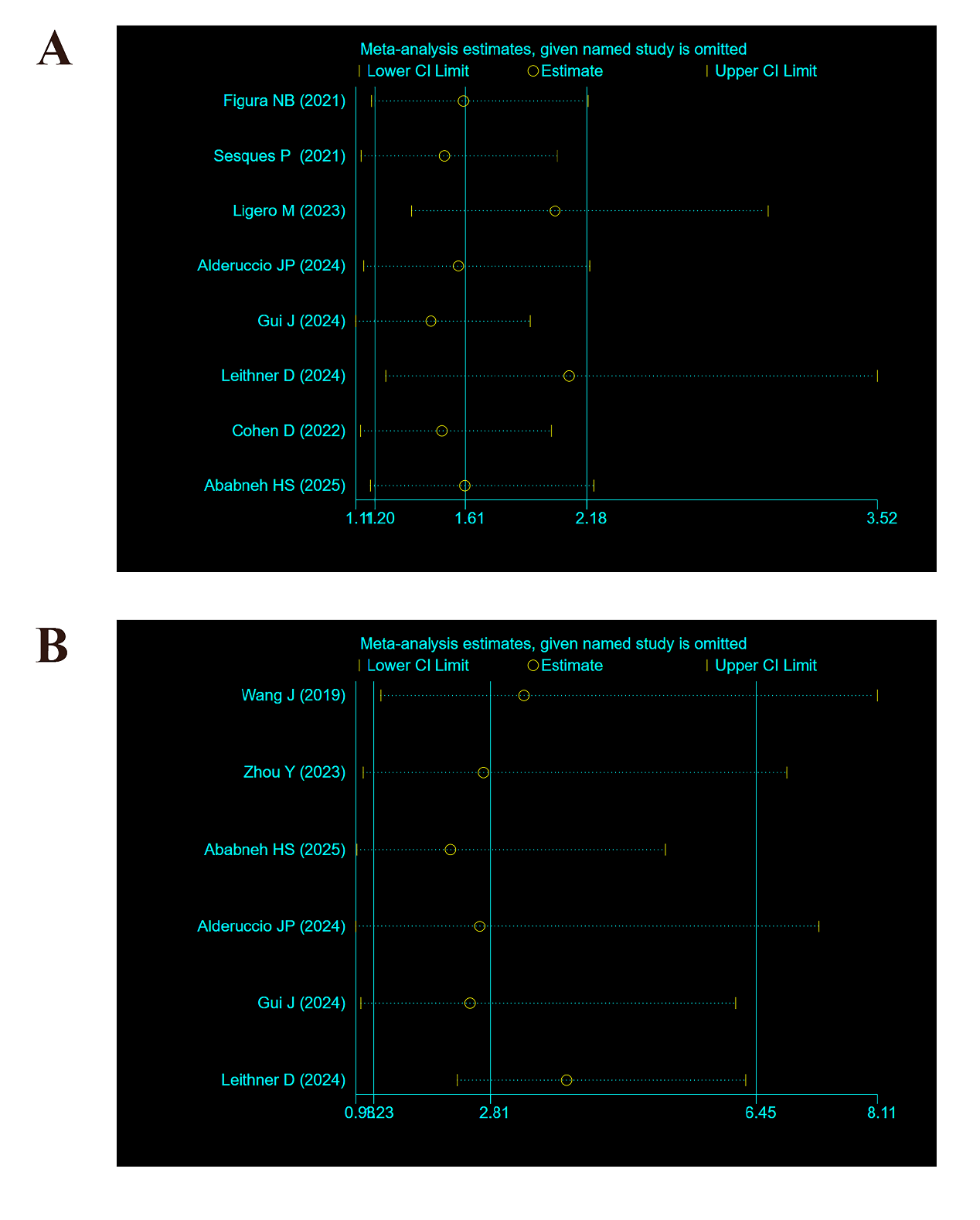
Figure S2** Sensitivity analysis of the prognostic effect of SUVmax (A) and MTV (B) on overall survival.

**
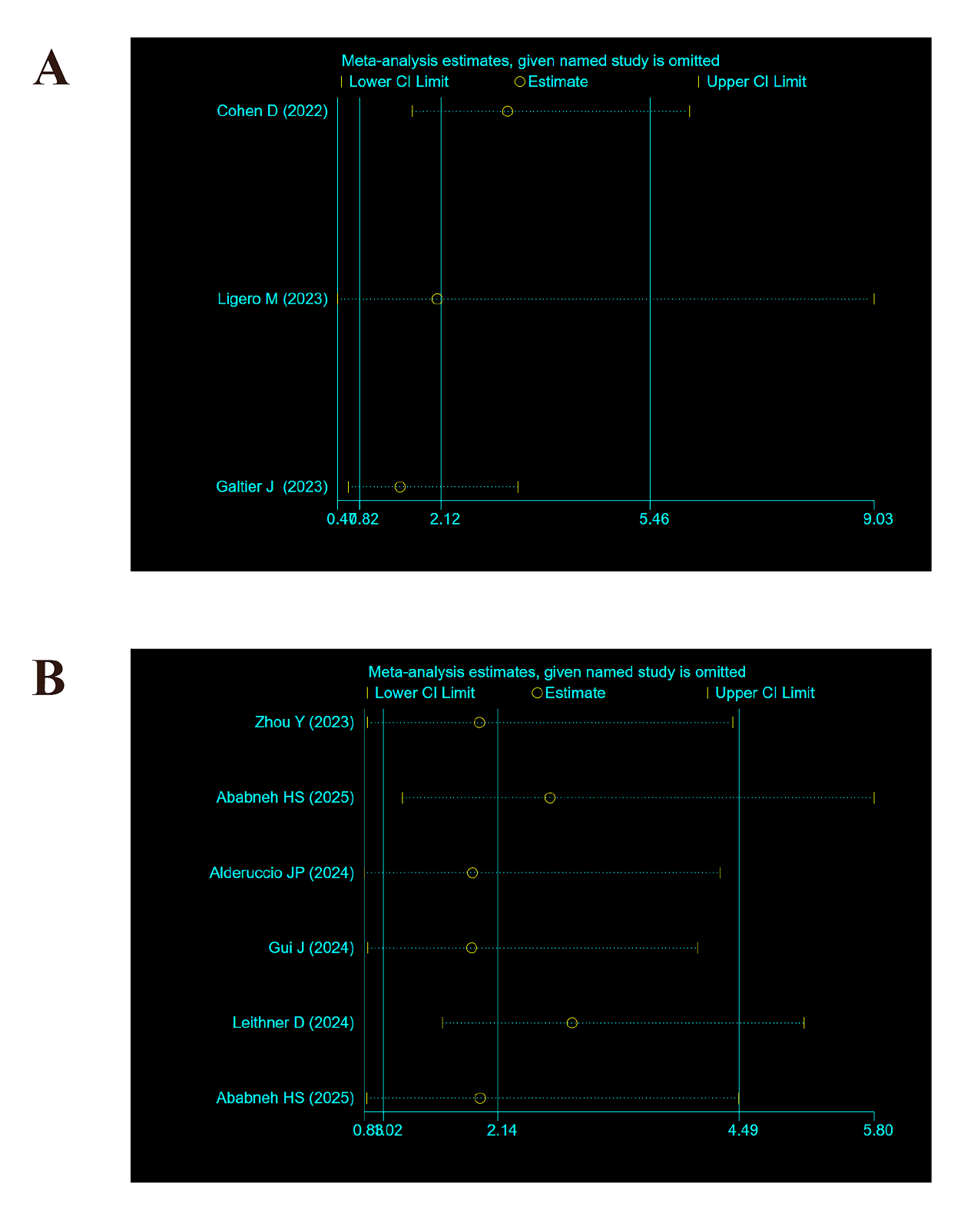
**

**Figure S3** Sensitivity analysis of the prognostic effect of TMTV (A) and TLG (B) on overall survival.

**
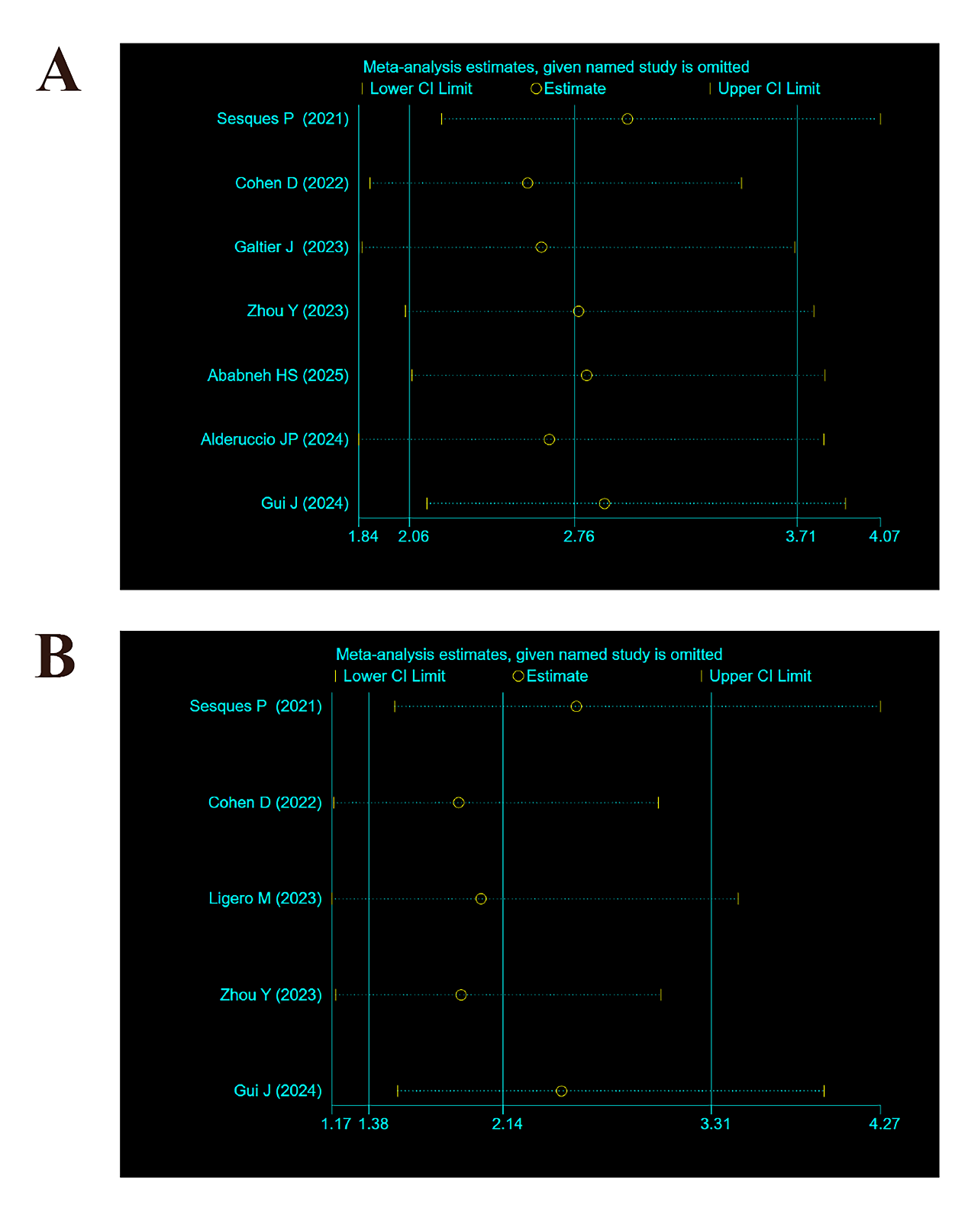
**

**Figure S4** Sensitivity analysis of the prognostic effect of LDH (A) and ECOG (B) on overall survival.

**
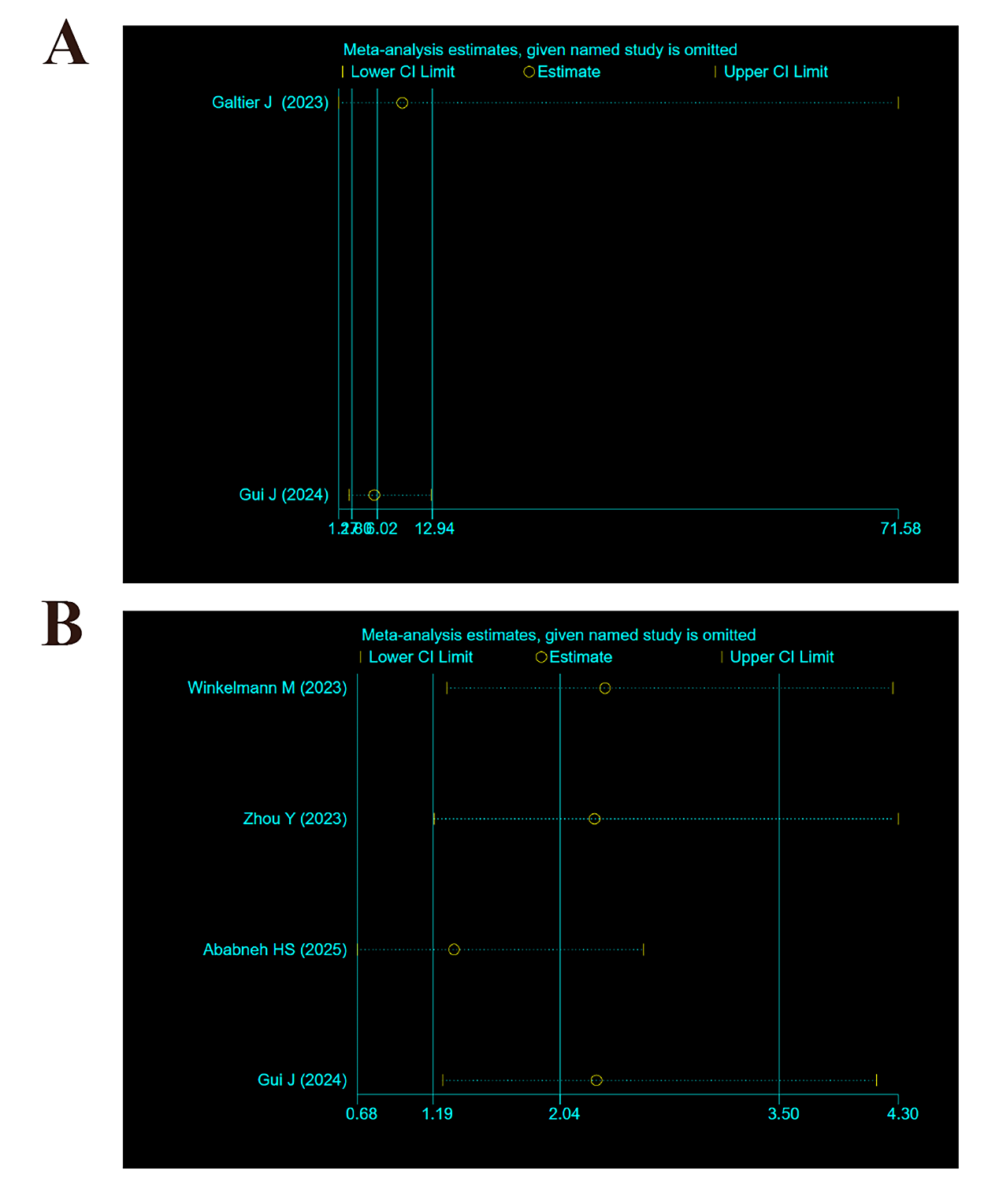
**

**Figure S5** Sensitivity analysis of the prognostic effect of DS (A) and IPI (B) on overall survival.

**
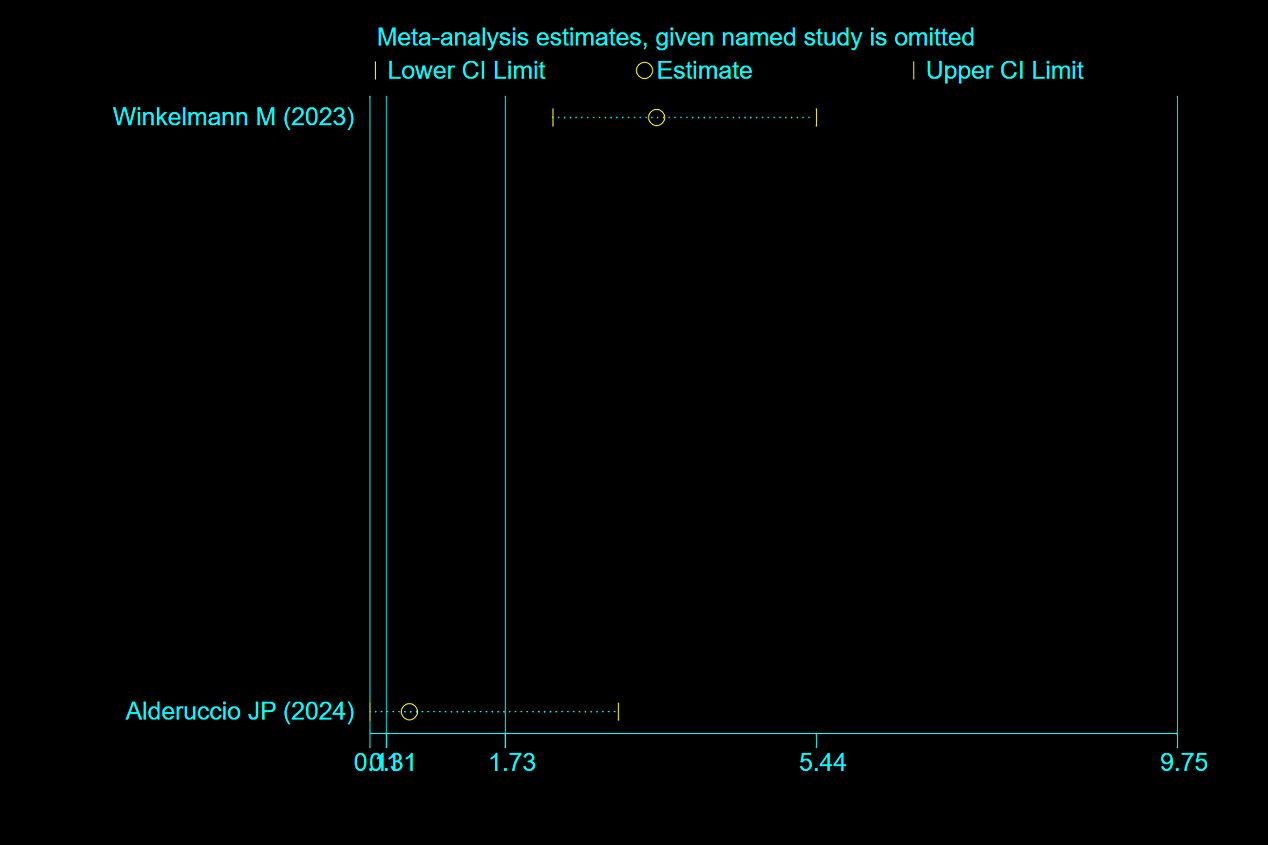
**

**Figure S6** Sensitivity analysis of the prognostic effect of IMPI (A) on overall survival.


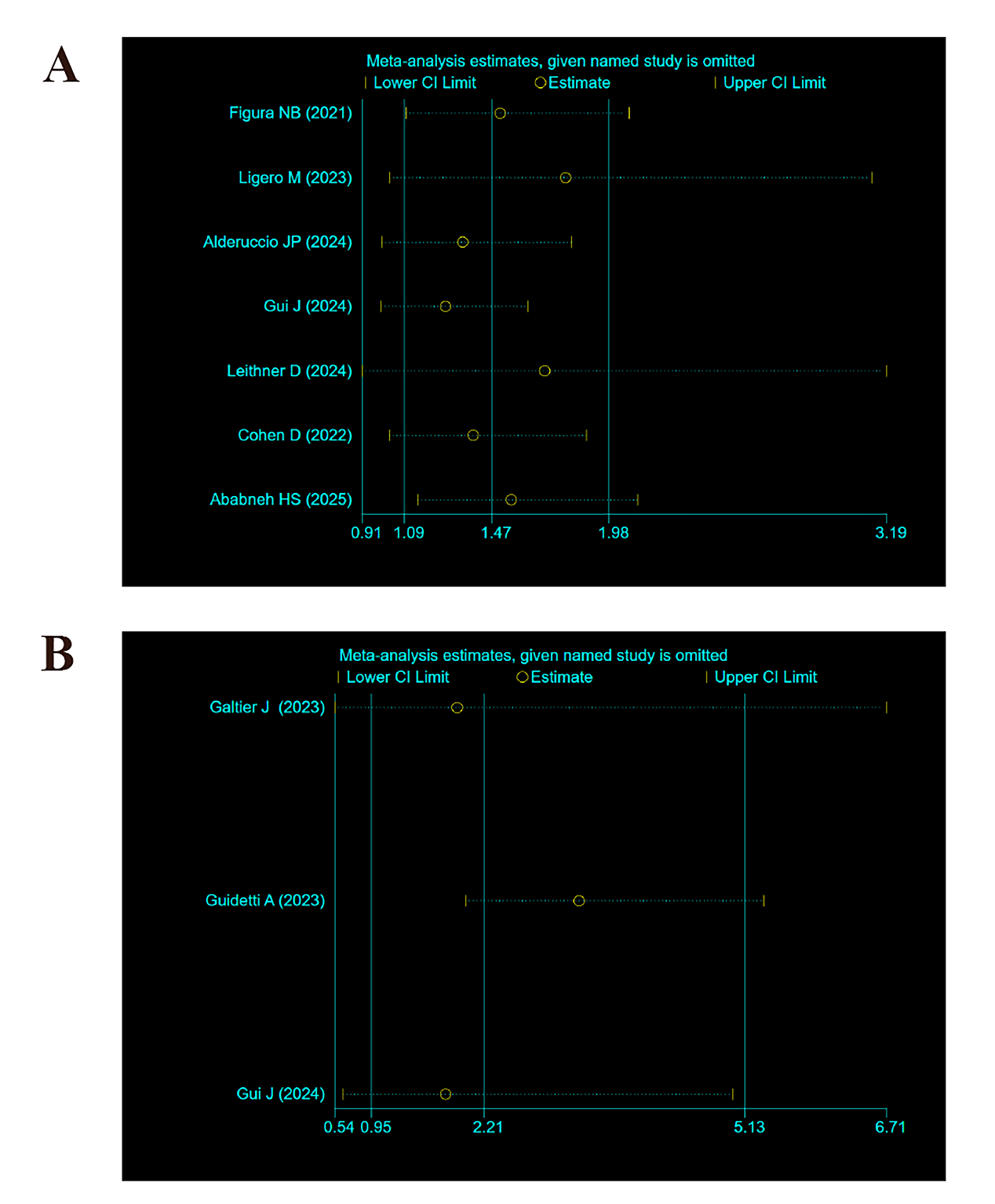


**Figure** **S7** Sensitivity analysis of the prognostic effect of SUVmax (A) and ΔSUVmax (B) on progression-free survival.

**
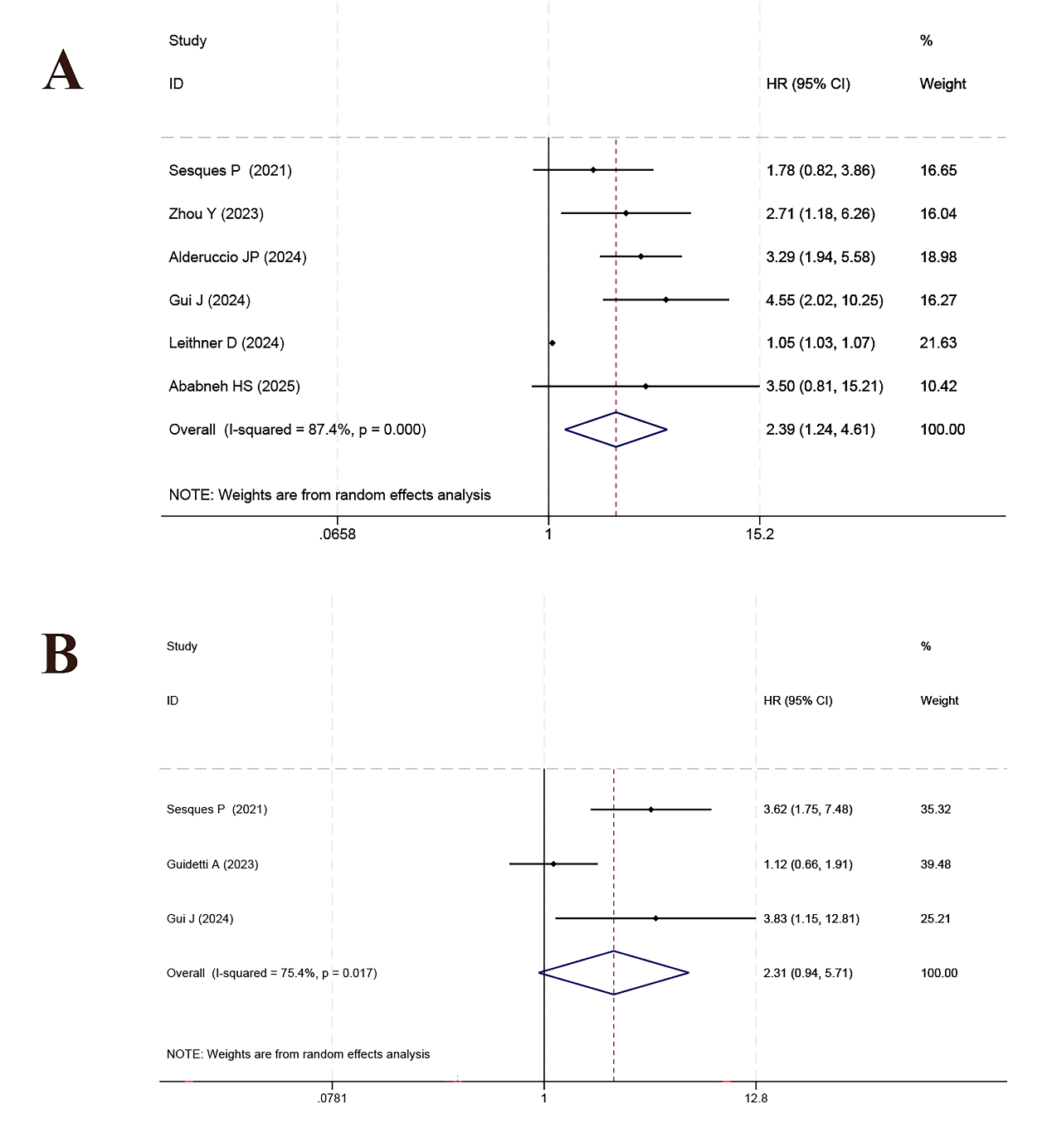
**

**Figure S8** Forest plot of the prognostic effect of MTV(A) and ΔMTV(B) on progression-free survival.


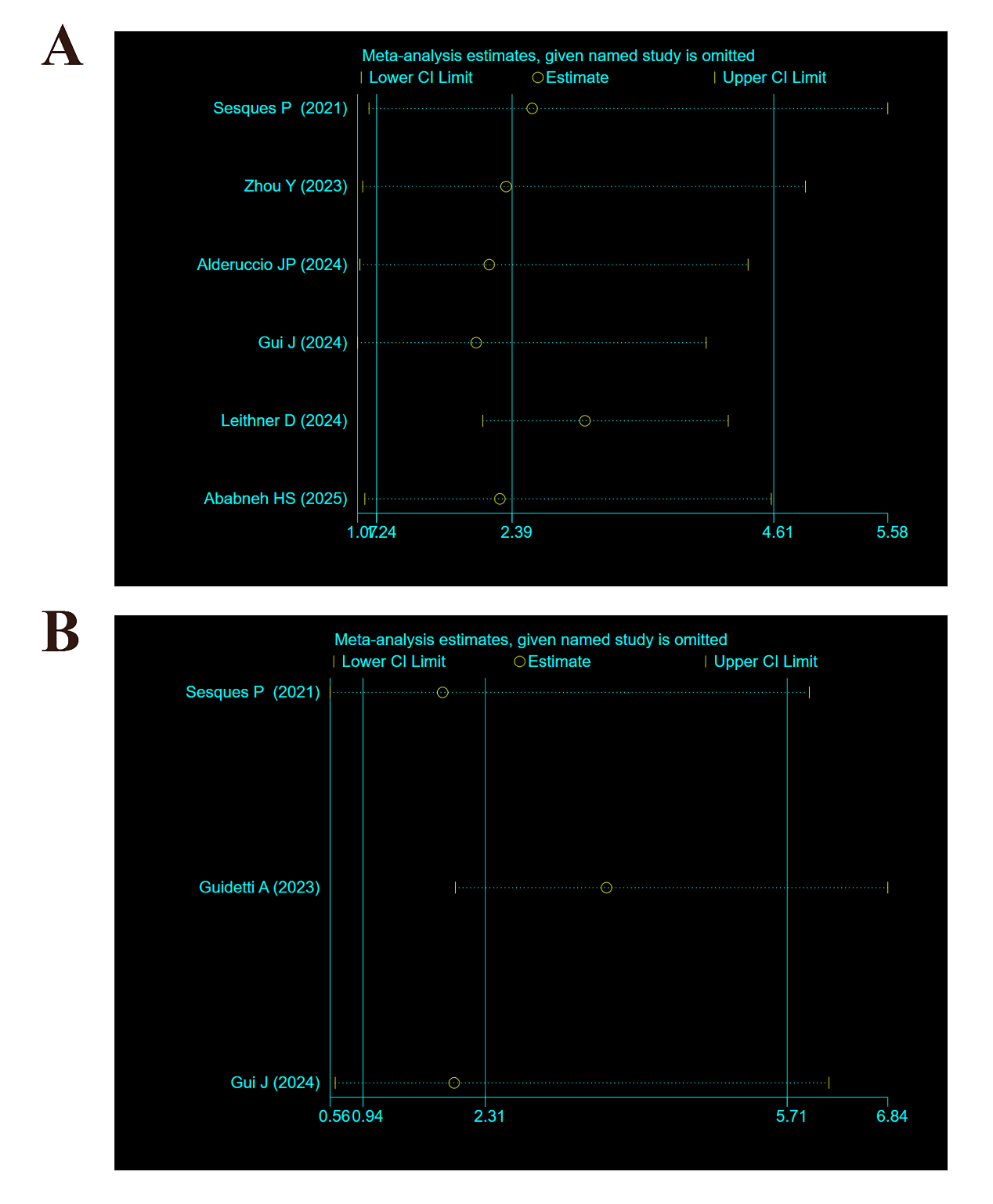


**Figure S9** Sensitivity analysis of the meta-analysis on the prognostic effect of MTV (A) and ΔMTV (B) on progression-free survival.

**
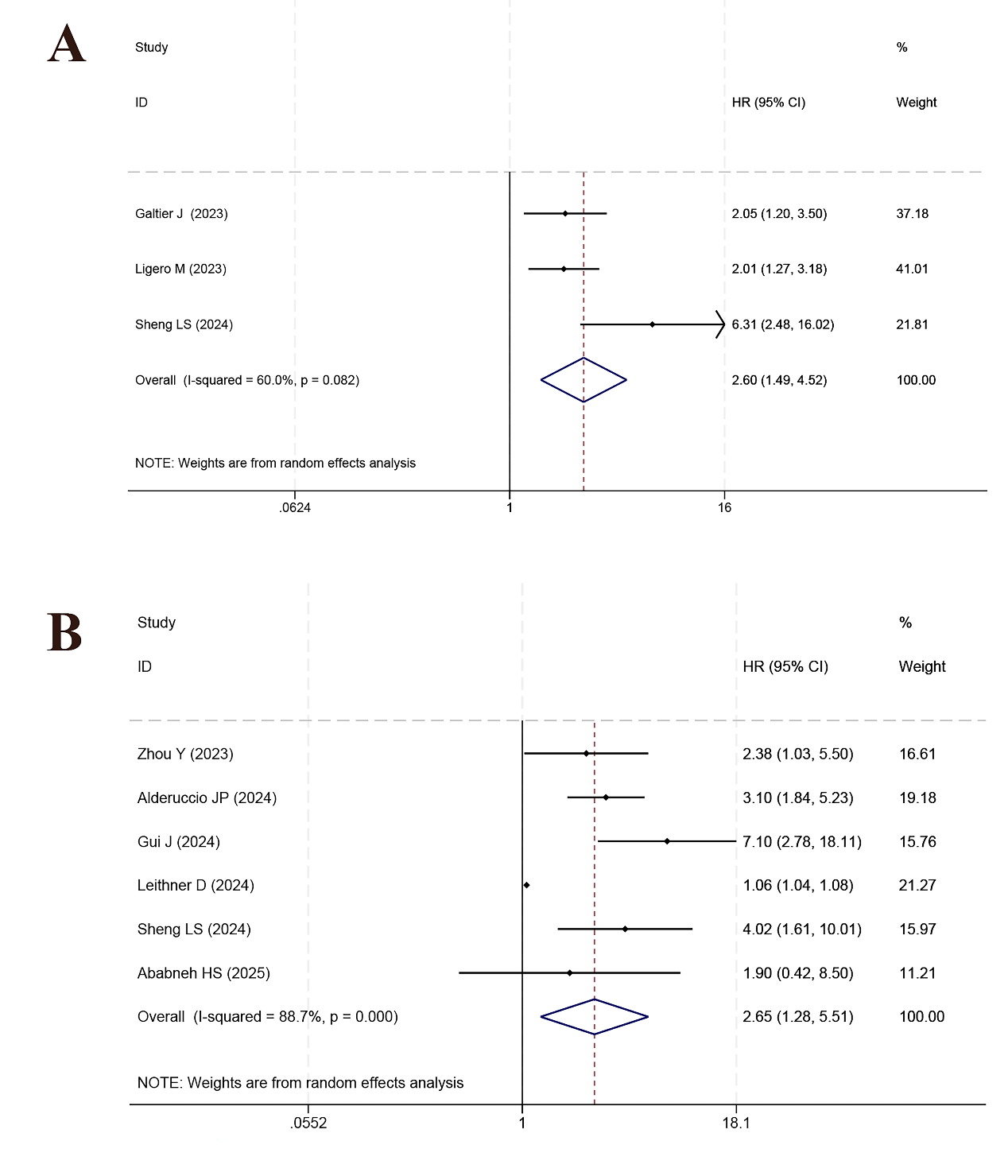
**

**Figure S10** Forest plot of the meta-analysis on the prognostic effect of TMTV(A) and TLG(B) on progression-free survival.


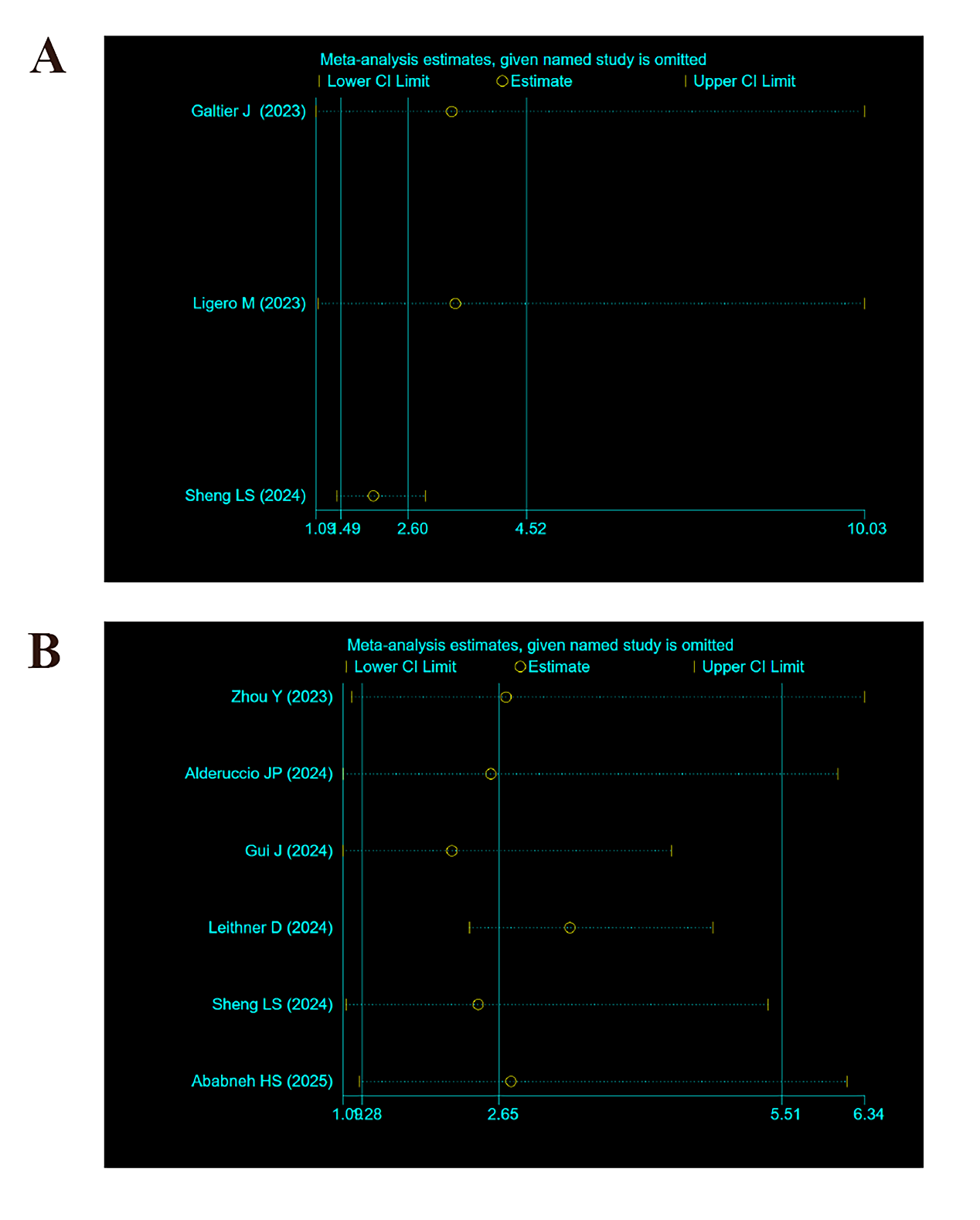


**Figure S11** Sensitivity analysis of the meta-analysis on the prognostic effect of TMTV (A) and TLG (B) on progression-free survival.


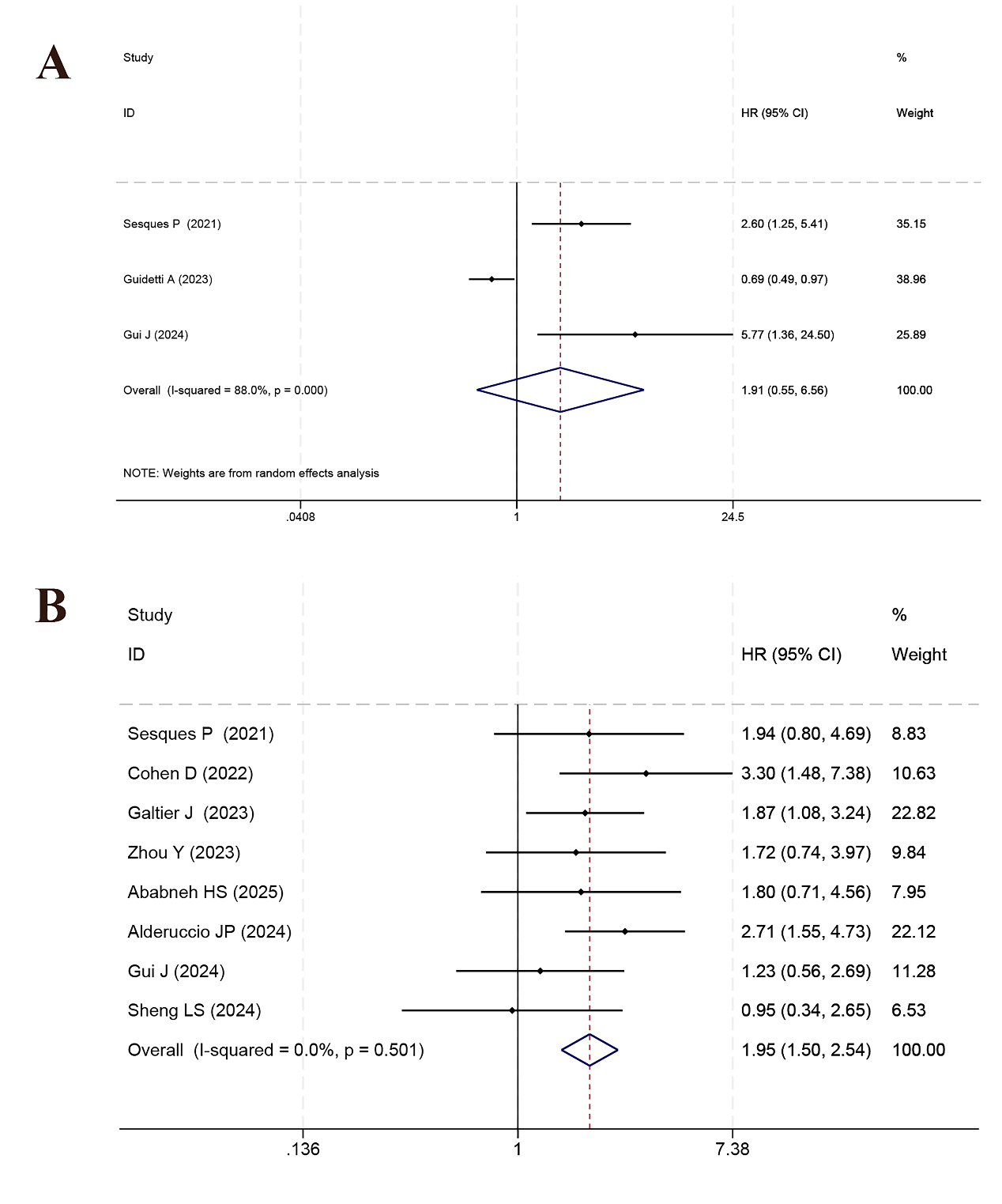


**Figure S12** Forest plot of the prognostic effect of ΔTLG(A) and LDH(B) on progression-free survival.


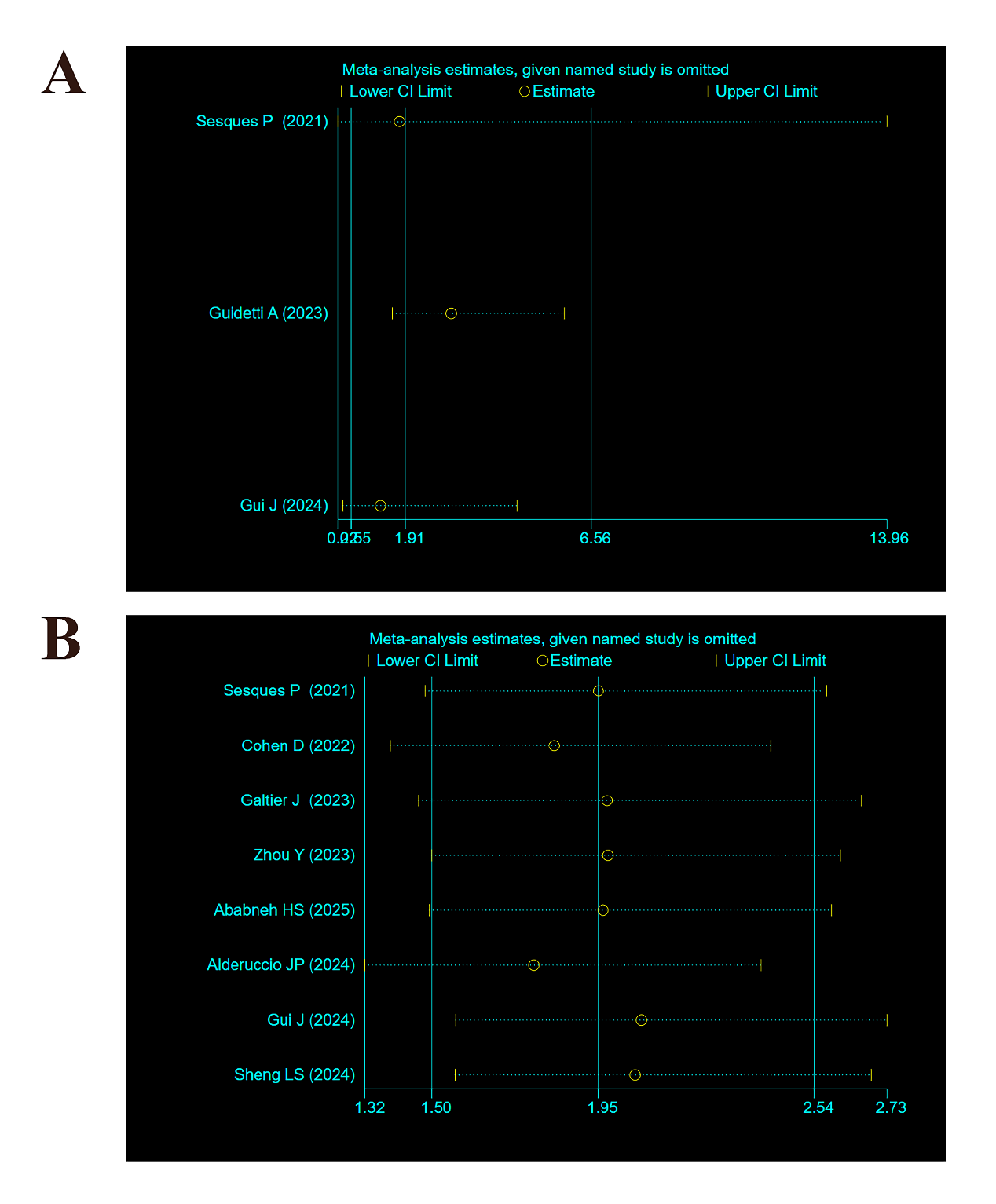


**Figure S13** Sensitivity analysis of the meta-analysis on the prognostic effect of ΔTLG (A) and LDH (B) on progression-free survival.

**
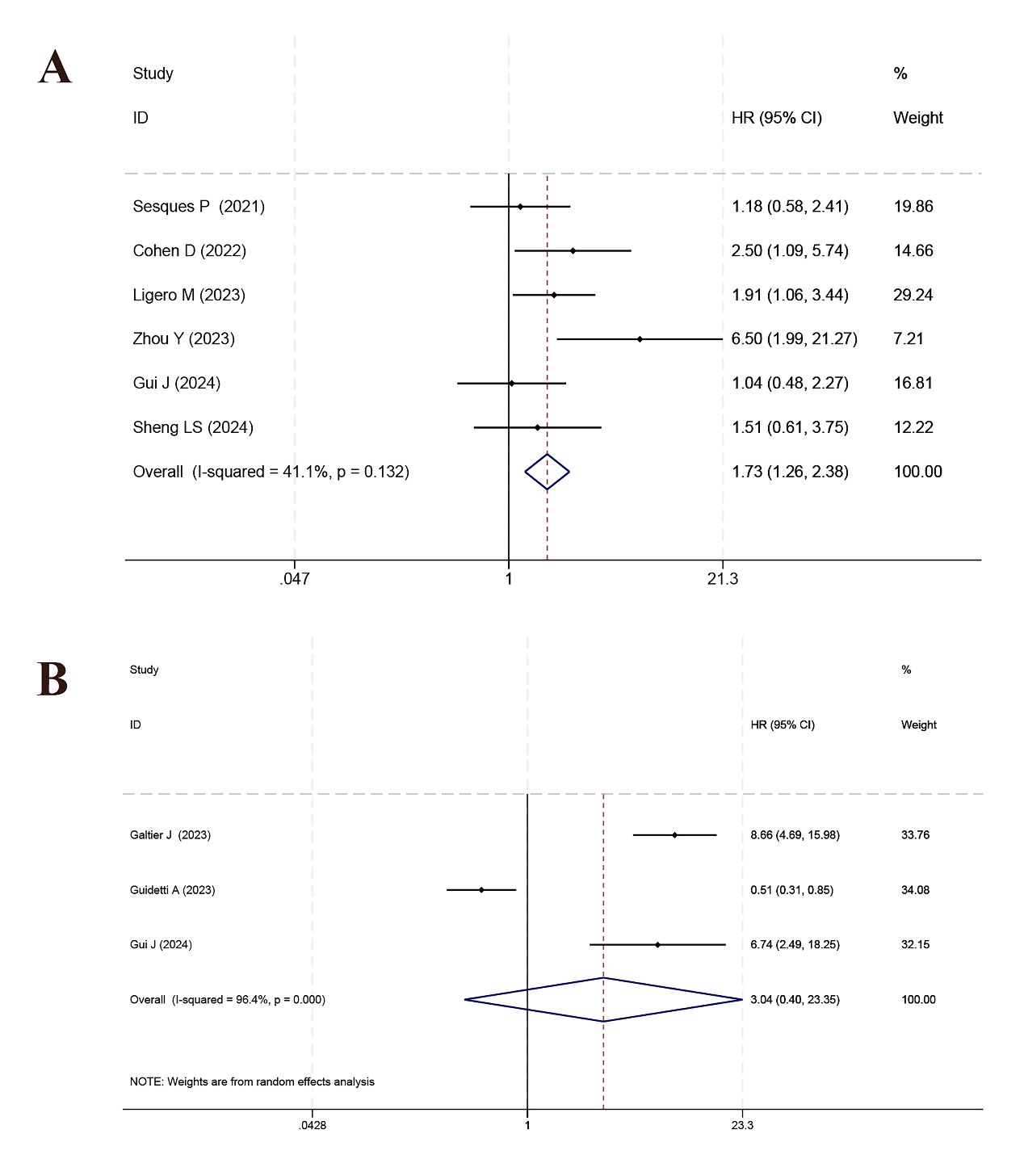
**

**Figure S14** Forest plot of the meta-analysis on the prognostic effect of ECOG(A) and DS(B) on progression-free survival.


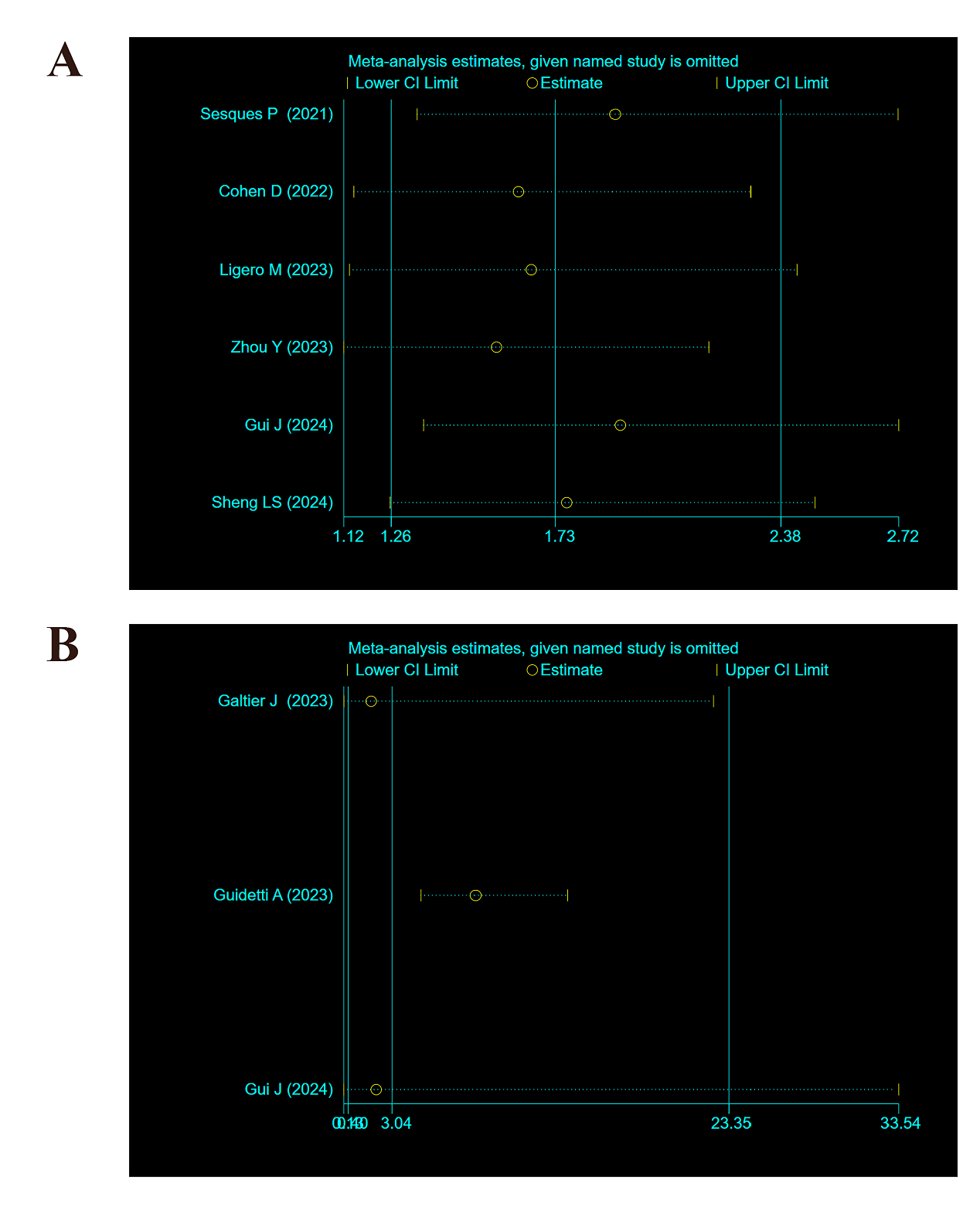


**Figure S15** Sensitivity analysis of the meta-analysis on the prognostic effect of ECOG (A) and DS (B) on progression-free survival.


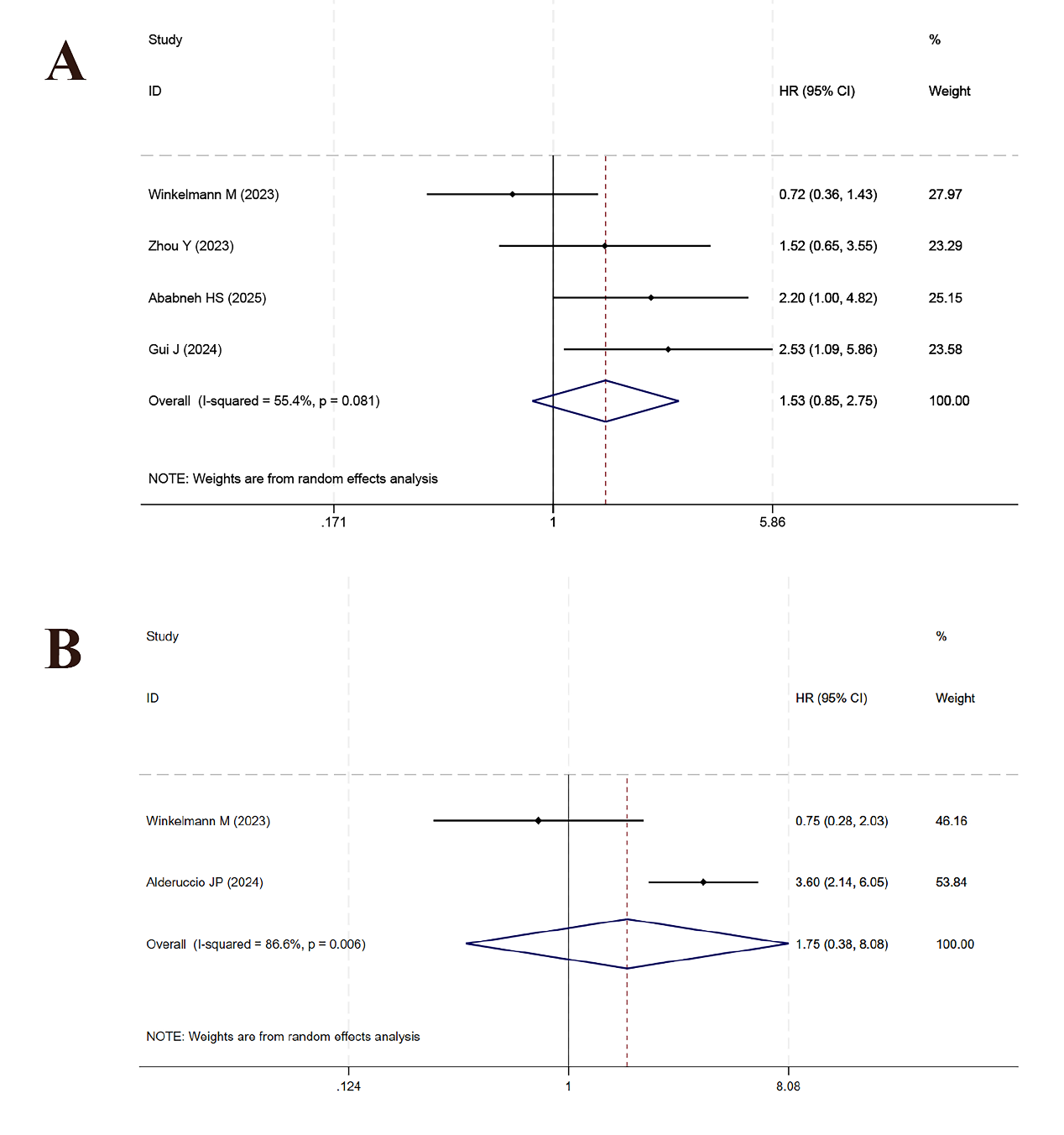


**Figure S16** Forest plot of the prognostic effect of IPI(A) and IMPI(B) on progression-free survival.


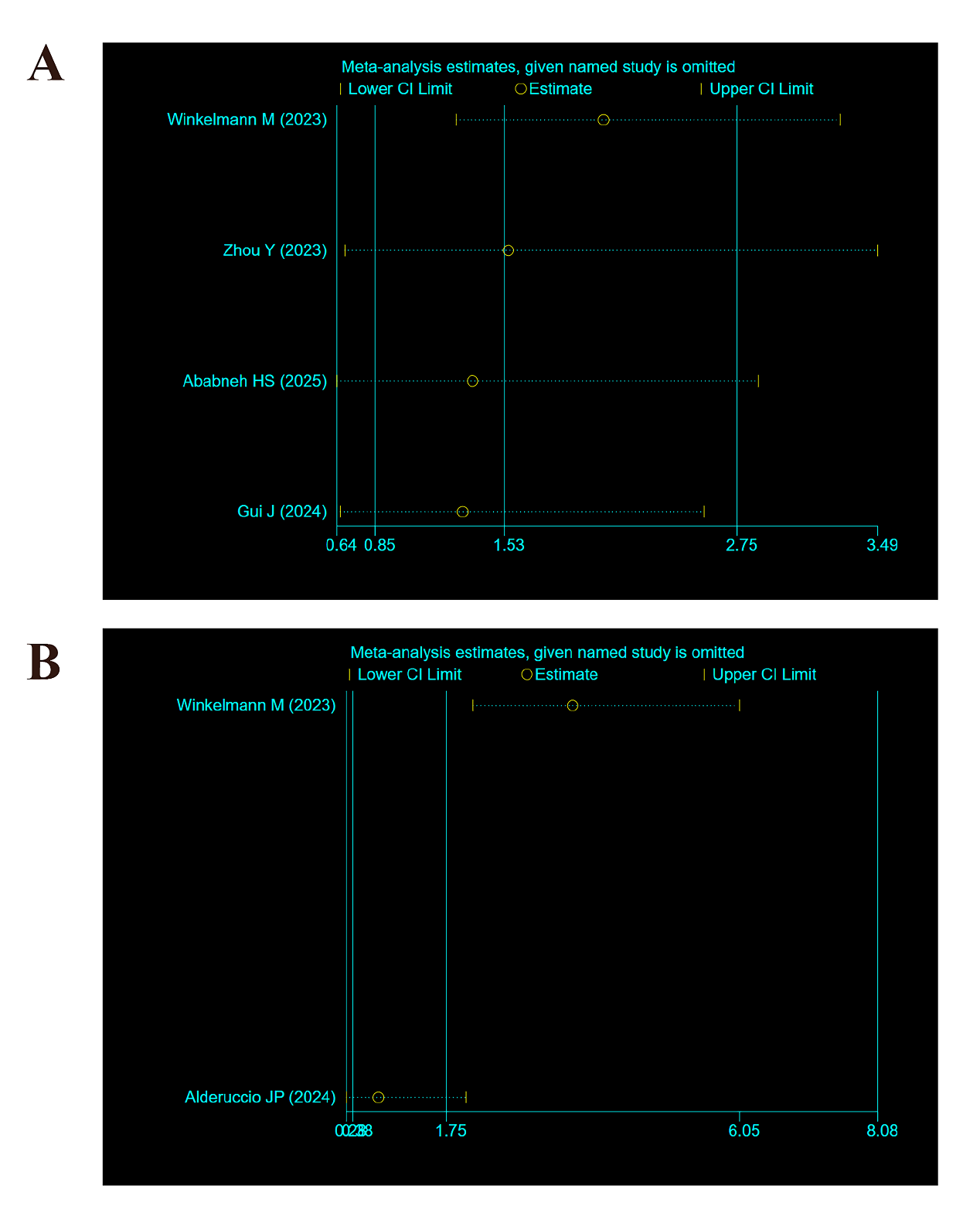


**Figure S17** Sensitivity analysis of the meta-analysis on the prognostic effect of IPI (A) and IMPI (B) on progression-free survival.


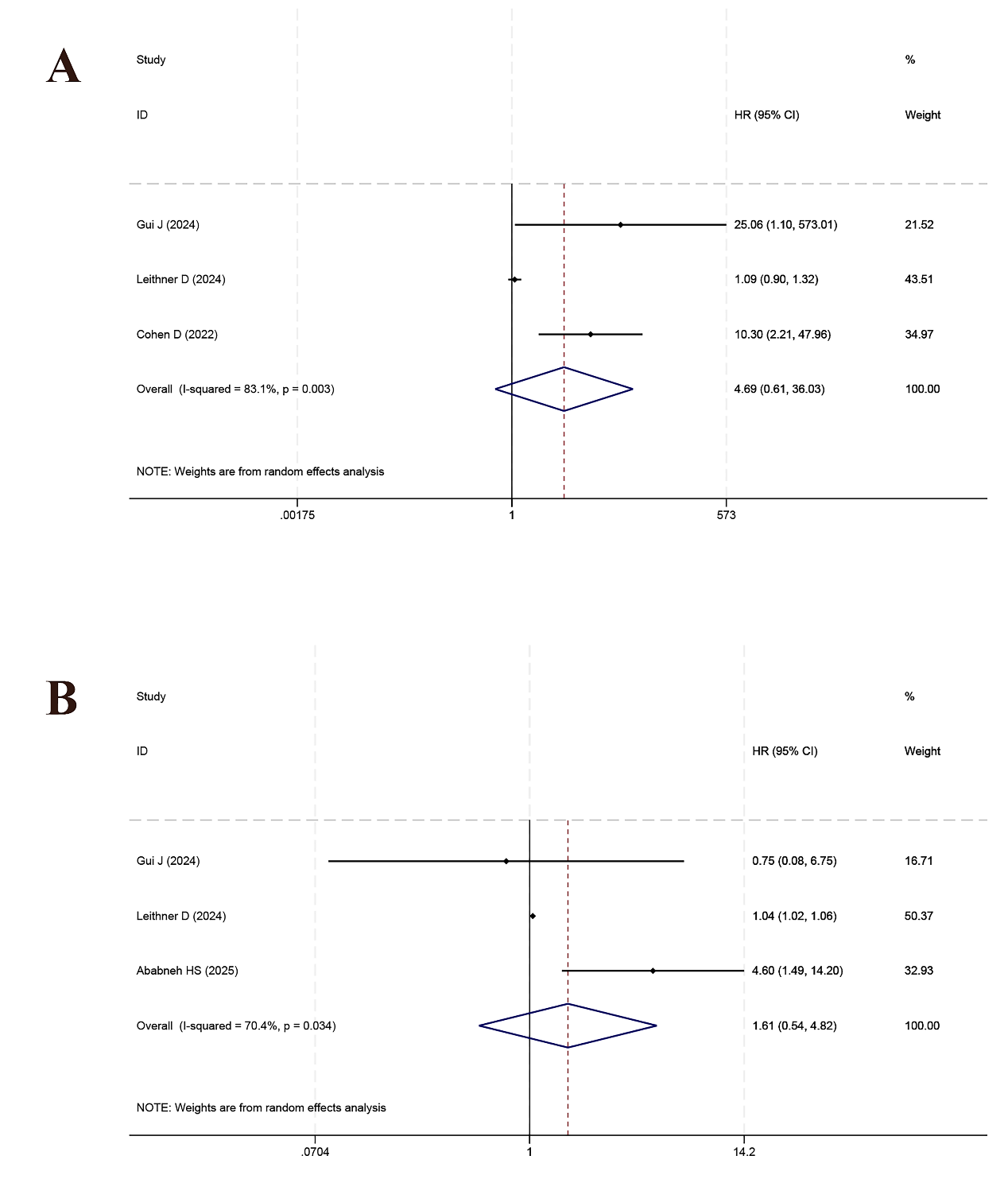


**Figure S18** Forest plot of the prognostic effect of SUVmax(A) and MTV(B) on overall survival.


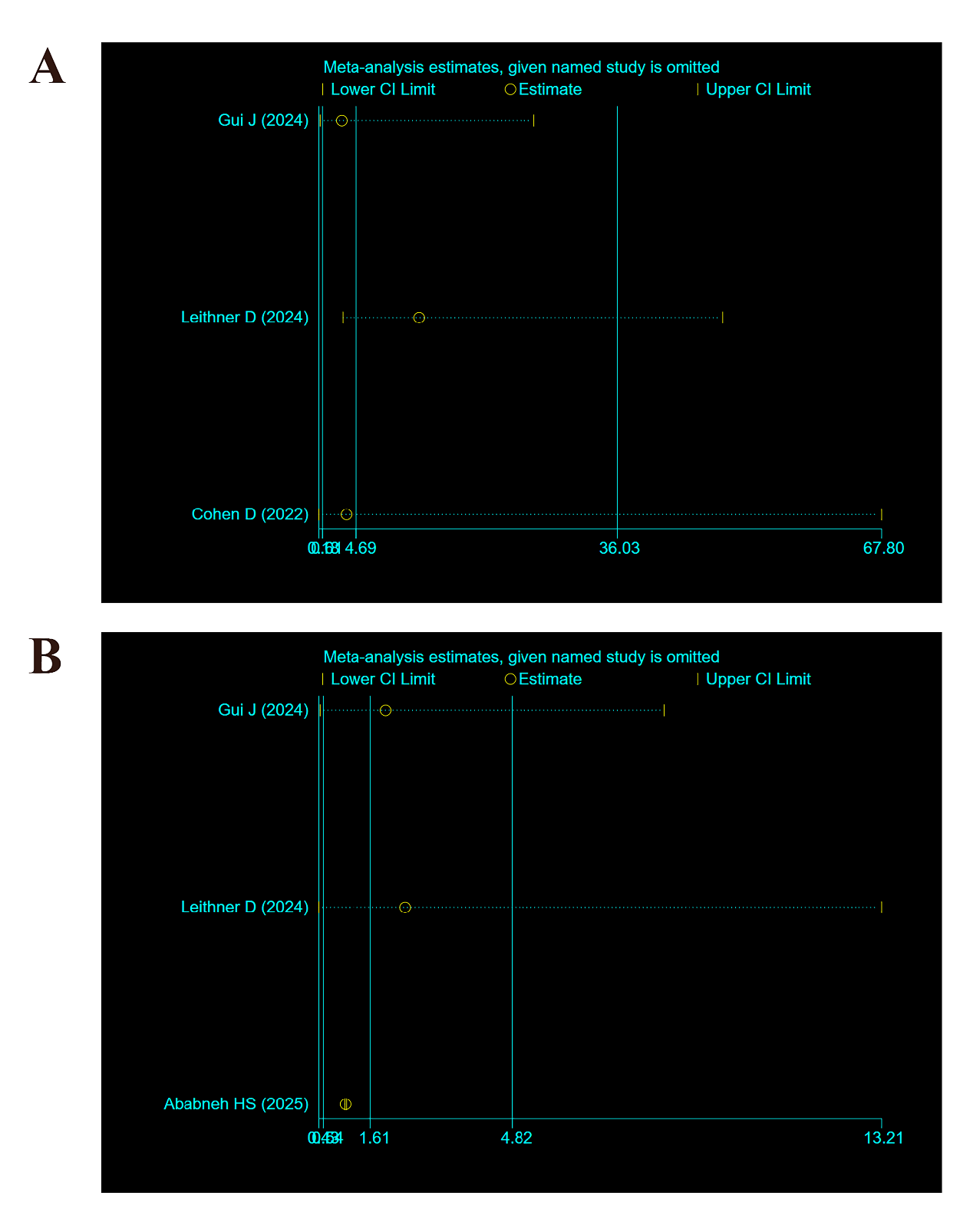


**Figure S19** Sensitivity analysis of the prognostic effect of SUVmax (A) and MTV (B) on overall survival.


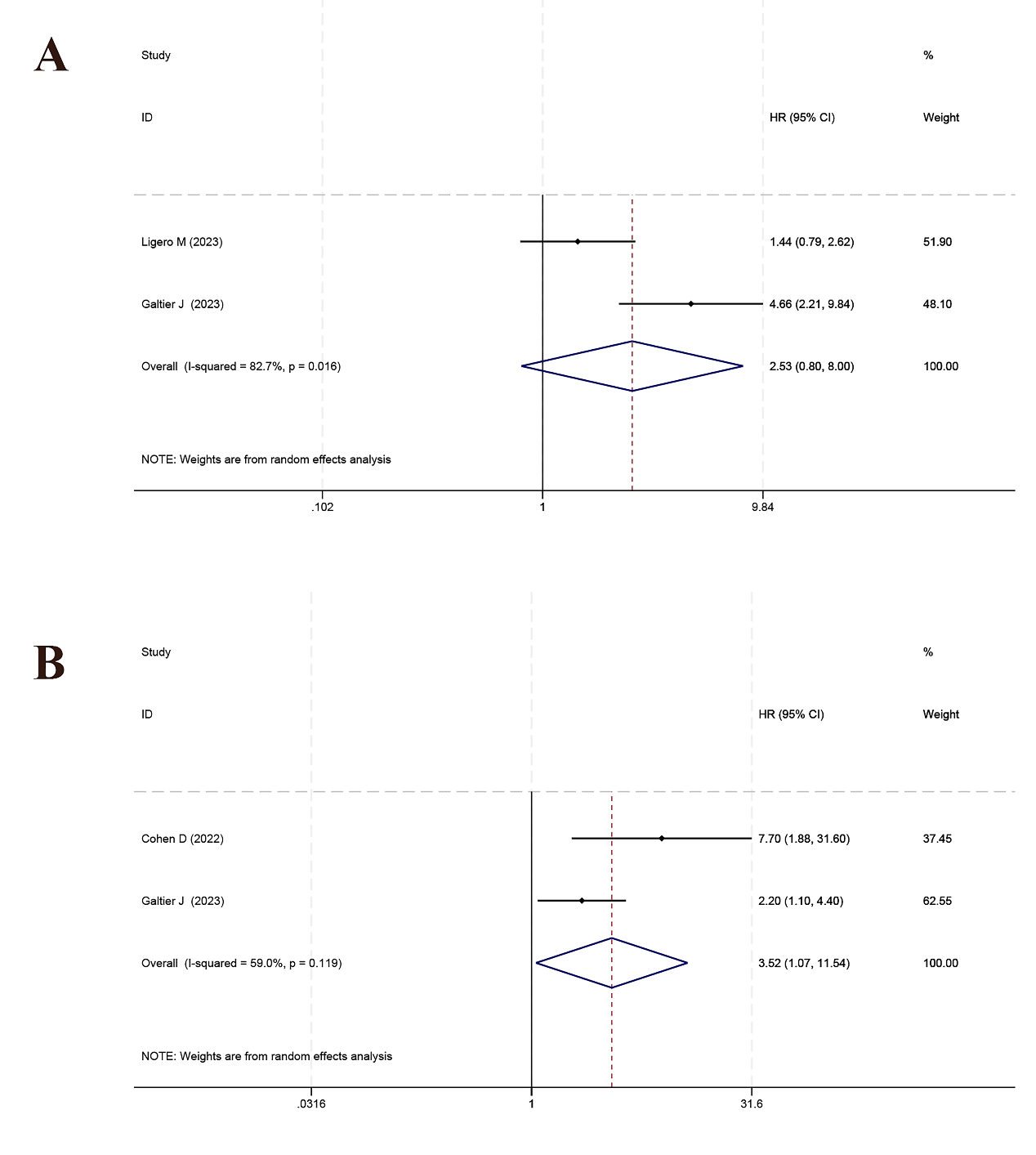


**Figure S20** Forest plot of the meta-analysis on the prognostic effect of TMTV(A) and LDH(B) on overall survival.


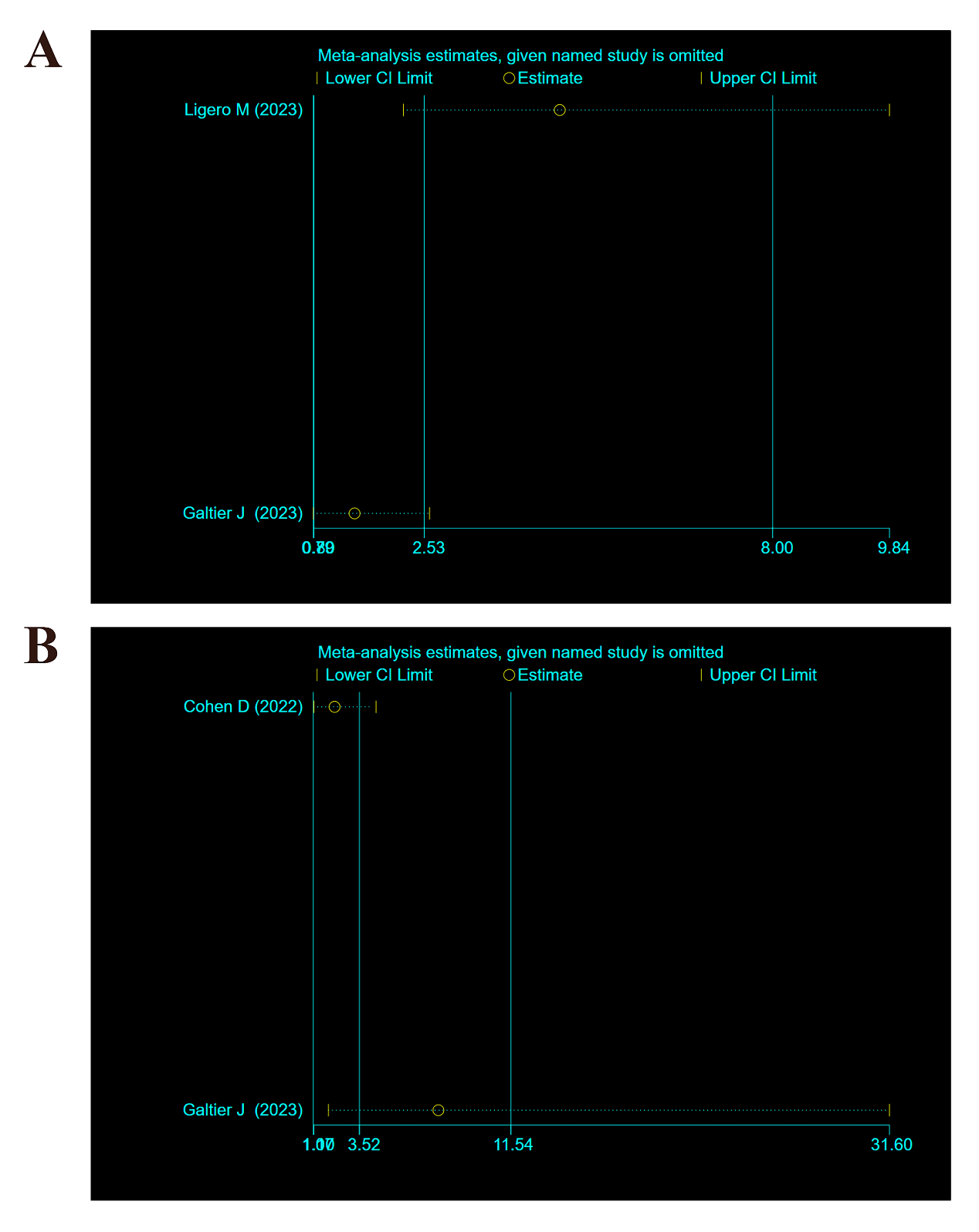


**Figure S21** Sensitivity analysis of the prognostic effect of TMTV (A) and LDH (B) on overall survival.

**
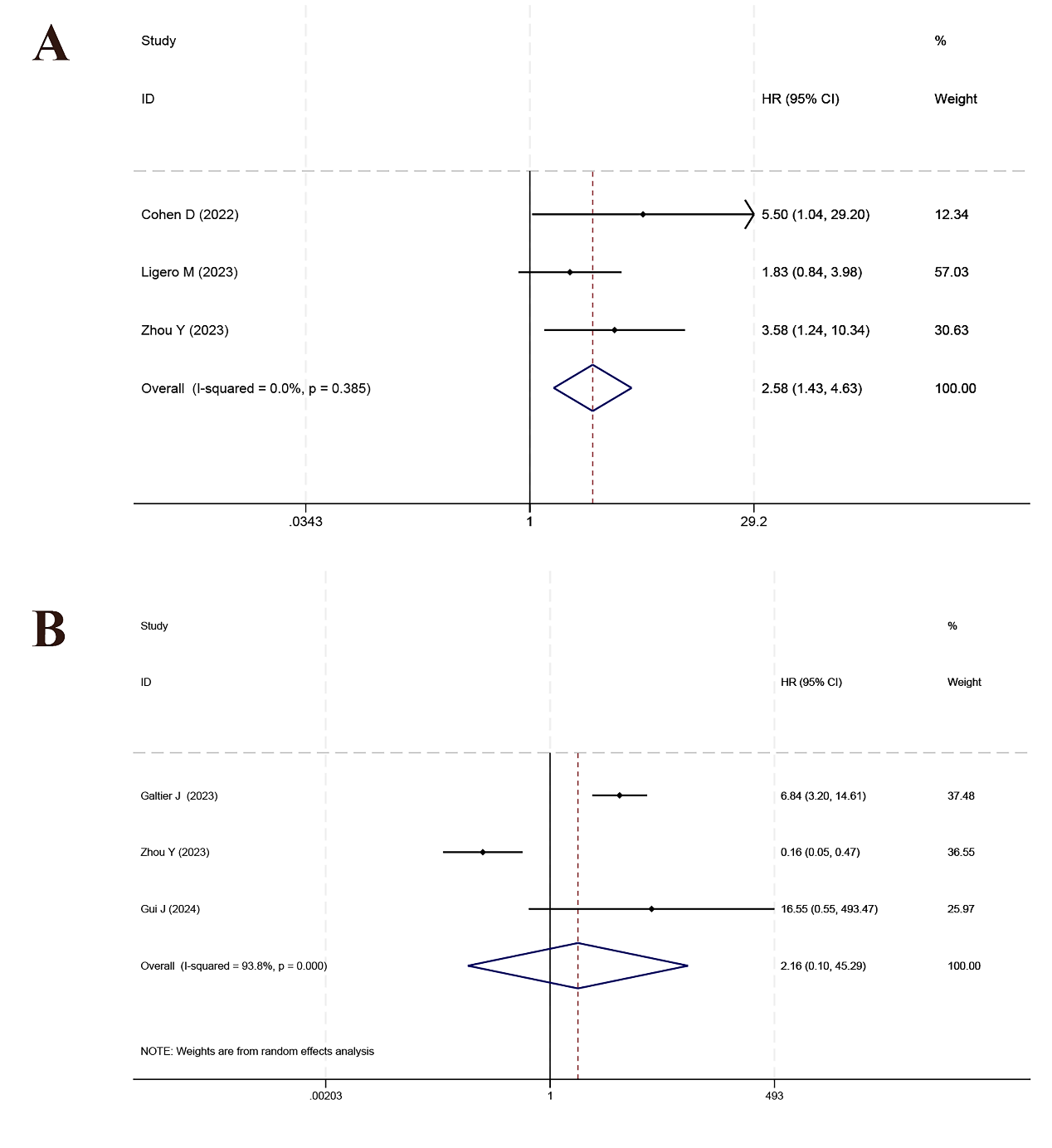
**

**Figure S22** Forest plot of the meta-analysis on the prognostic effect of ECOG(A) and DS(B) on overall survival.


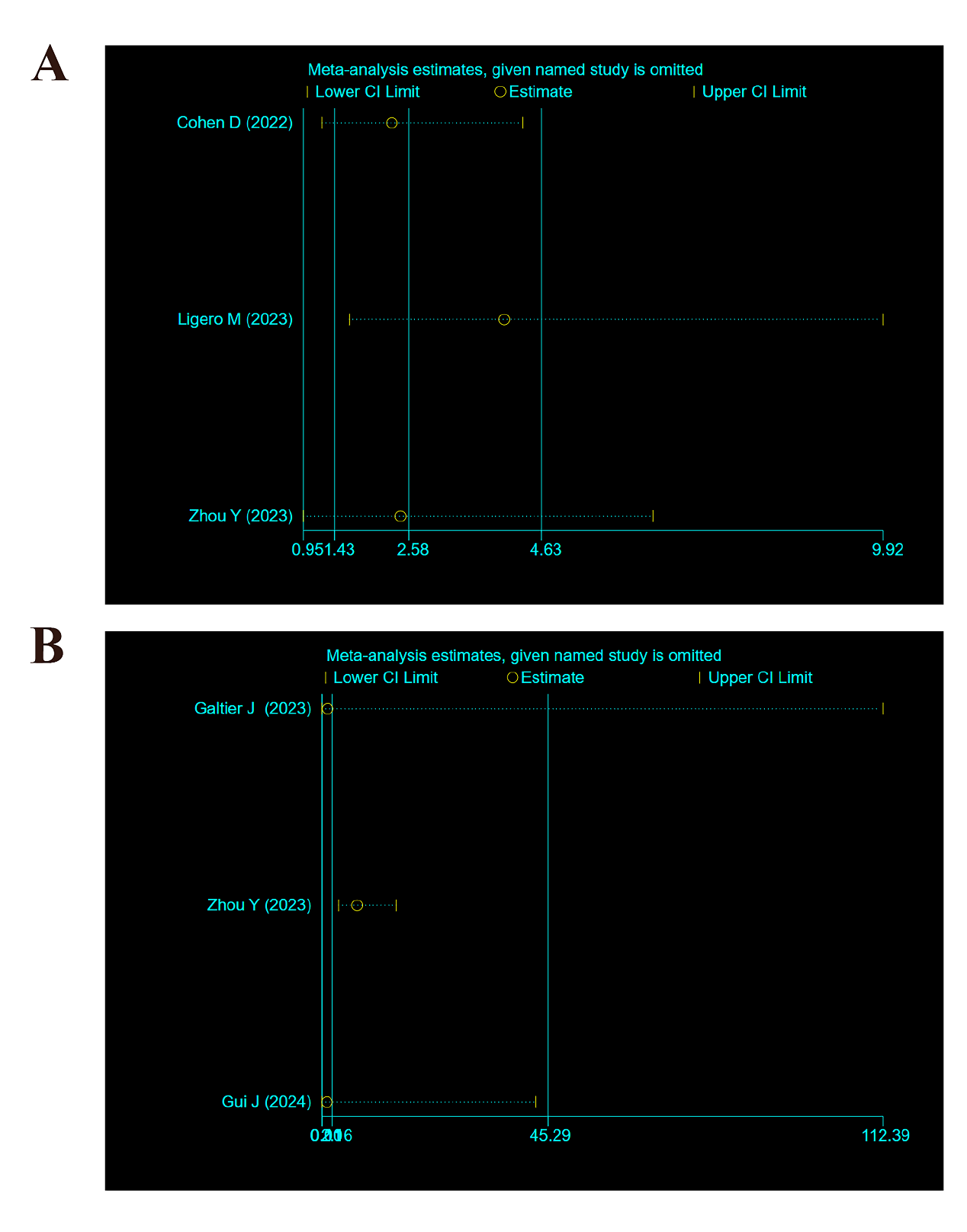


**Figure S23** Sensitivity analysis of the prognostic effect of ECOG (A) and DS (B) on overall survival.

**
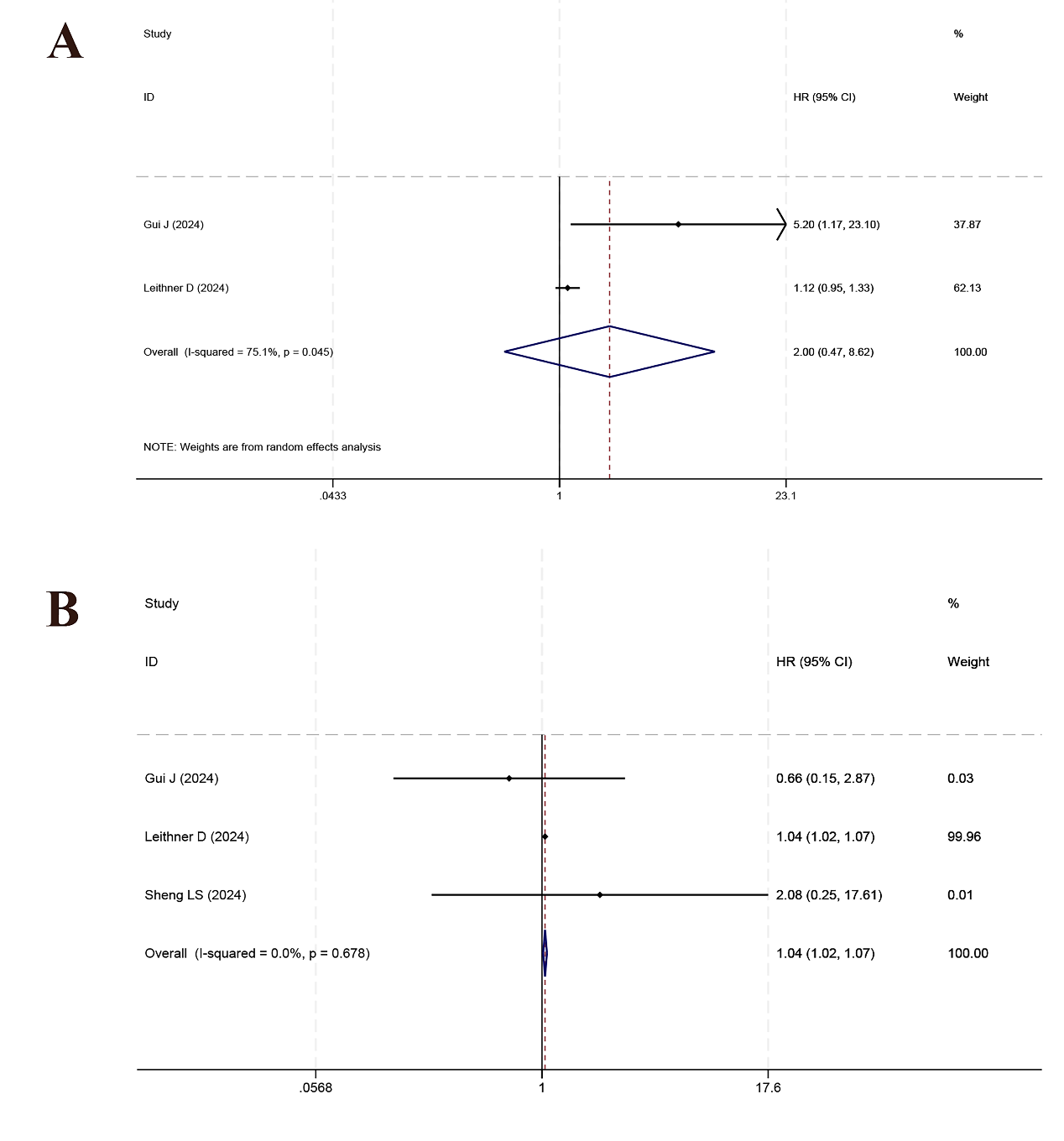
**

**Figure S24** Forest plot of the meta-analysis on the prognostic effect of SUVmax(A) and MTV(B) on progression-free survival.


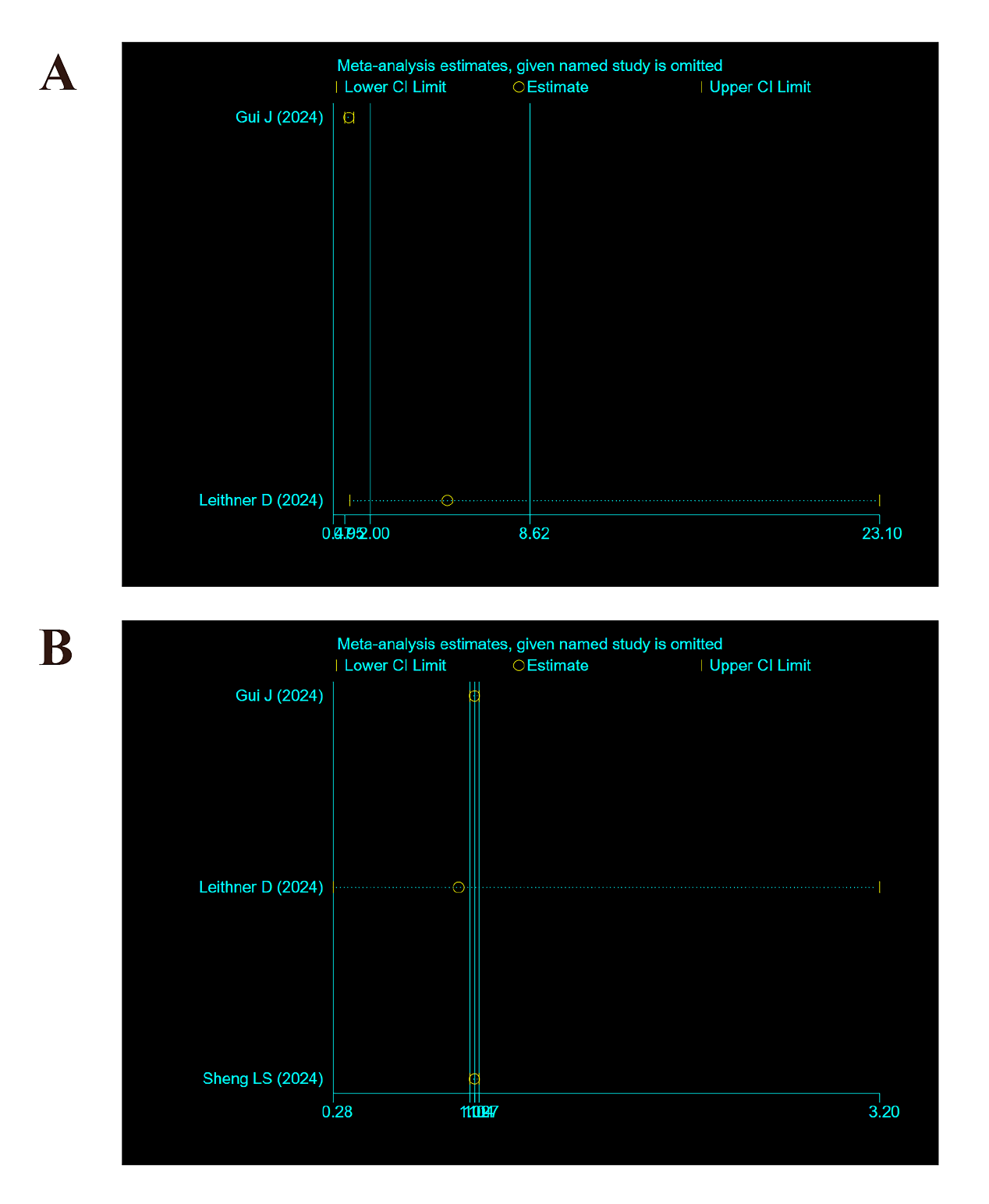


**Figure S25** Sensitivity analysis of the prognostic effect of SUVmax (A) and MTV (B) on progression-free survival.


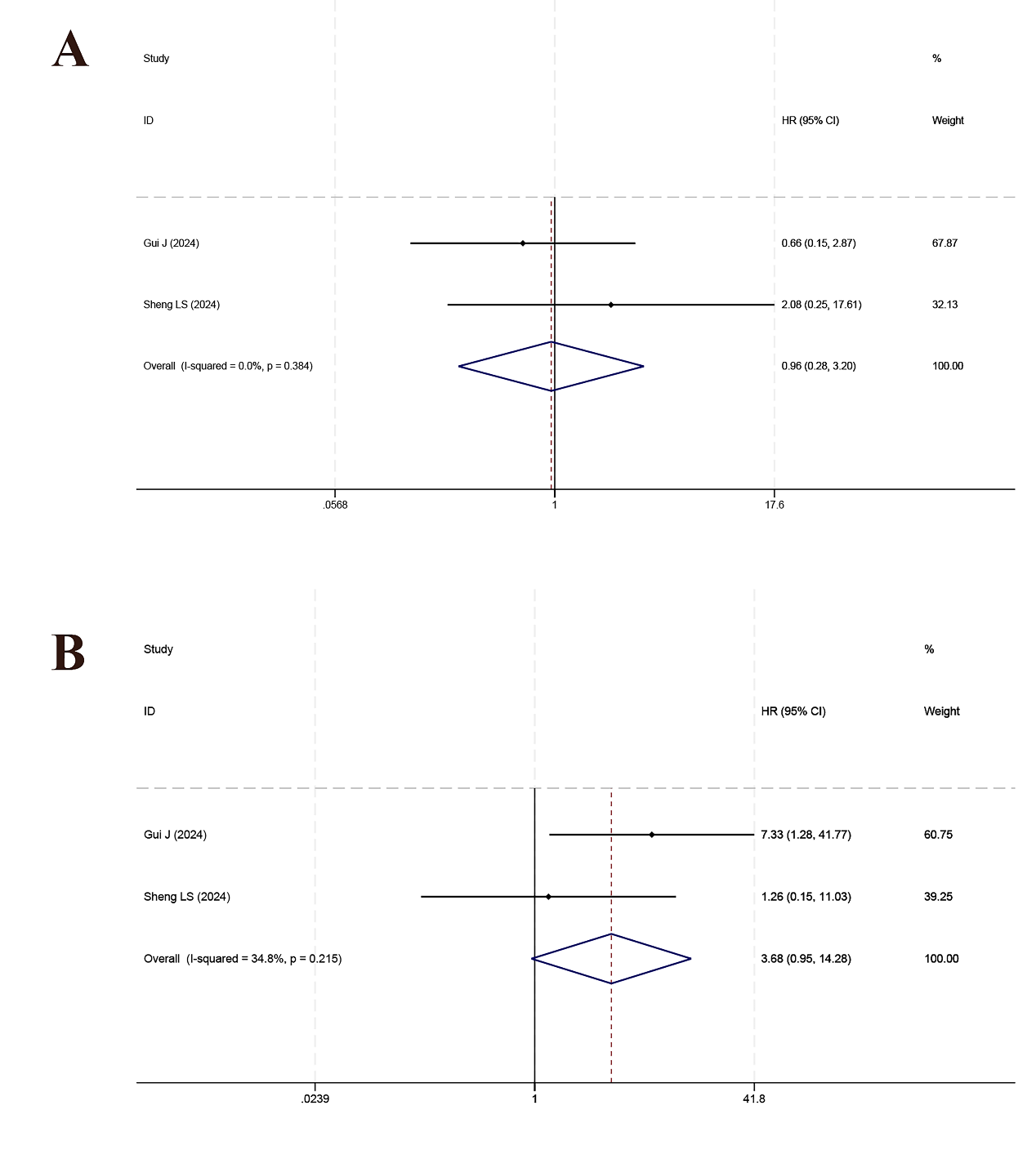


**Figure S26** Forest plot of the meta-analysis on the prognostic effect of MTV(A) and TLG(B) on progression-free survival.


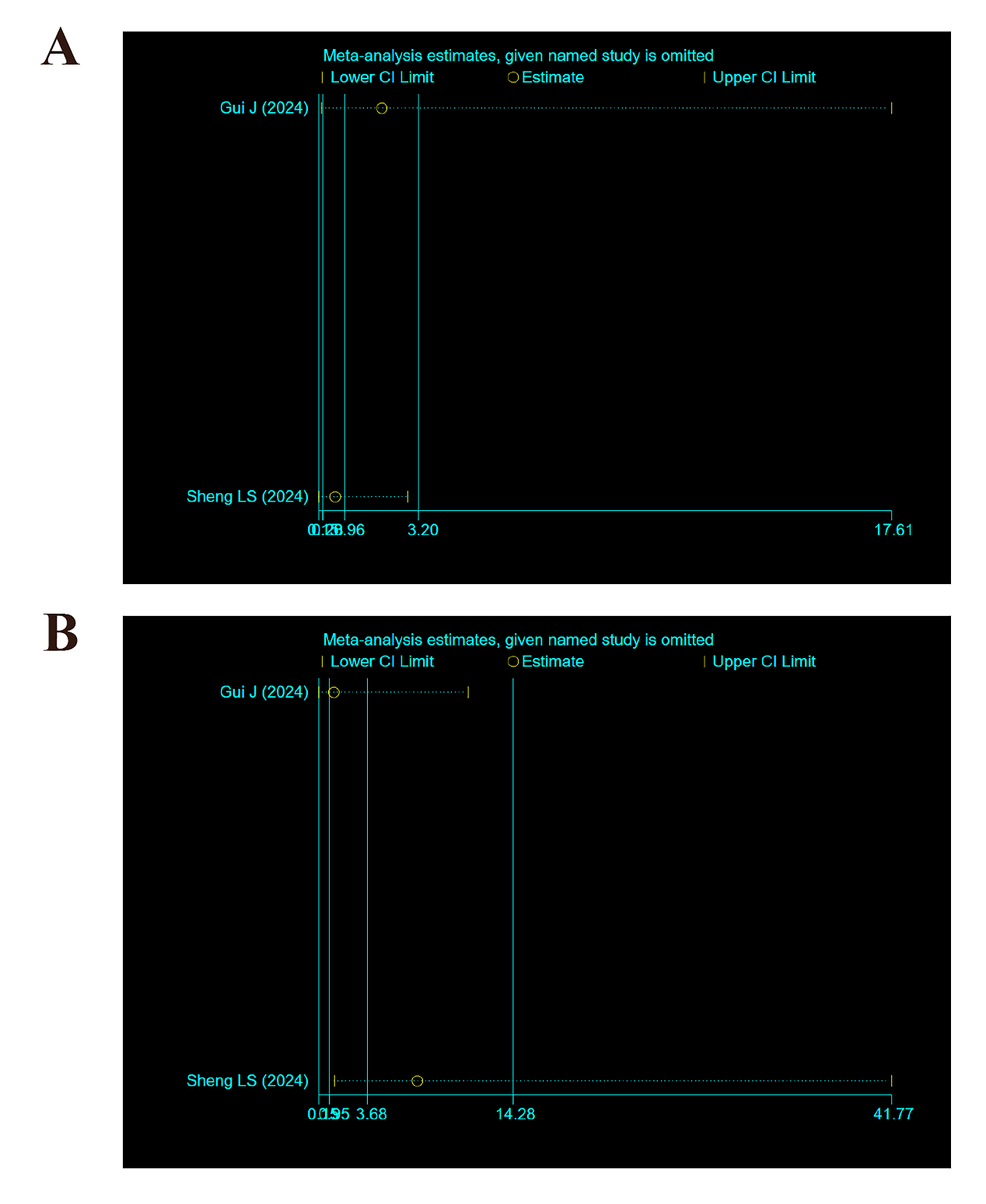


**Figure S27** Sensitivity analysis of the prognostic effect of MTV (A) and TLG (B) on progression-free survival.

**
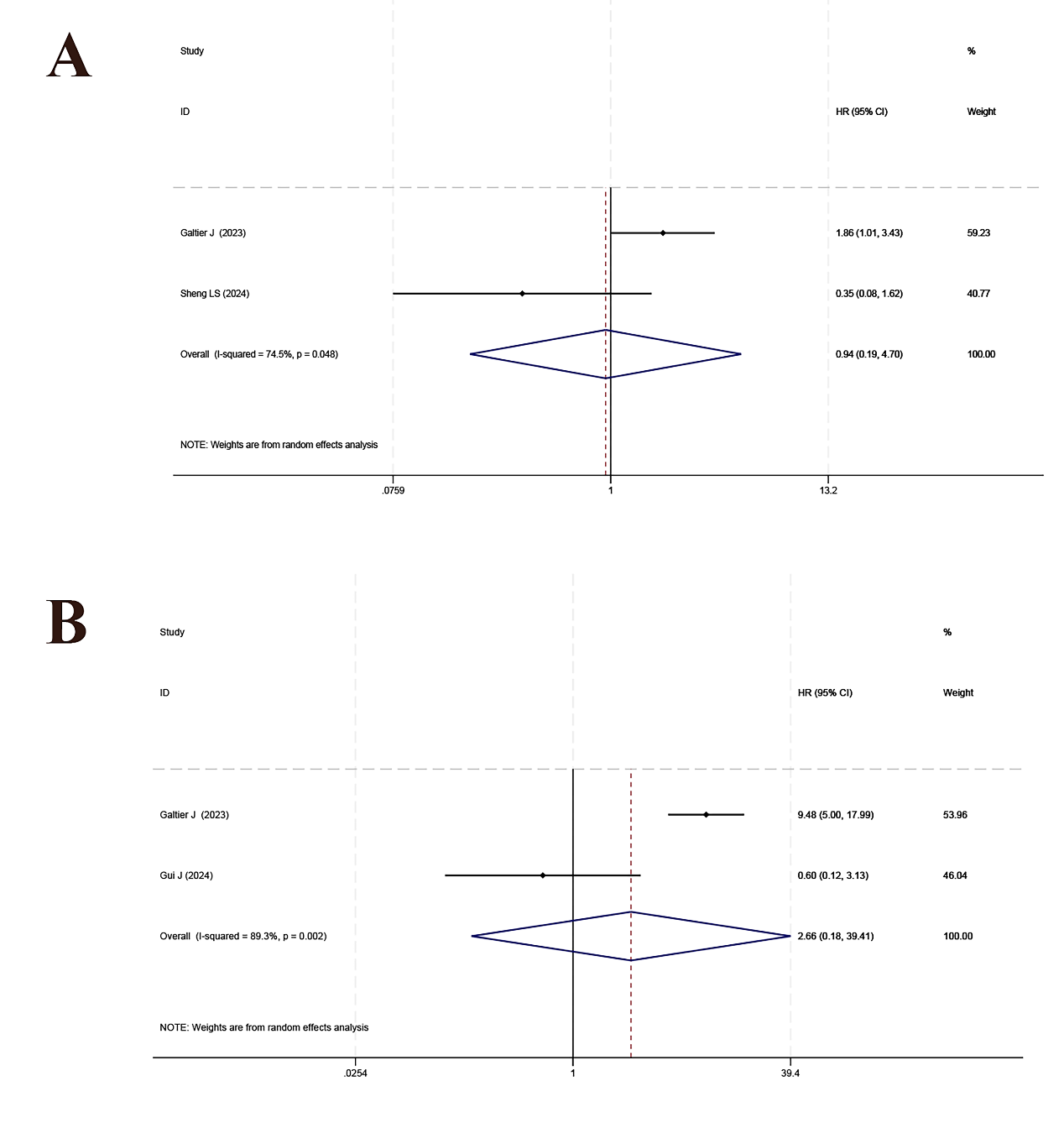
**

**Figure S28** Forest plot of the meta-analysis on the prognostic effect of LDH(A) and DS(B) on progression-free survival.


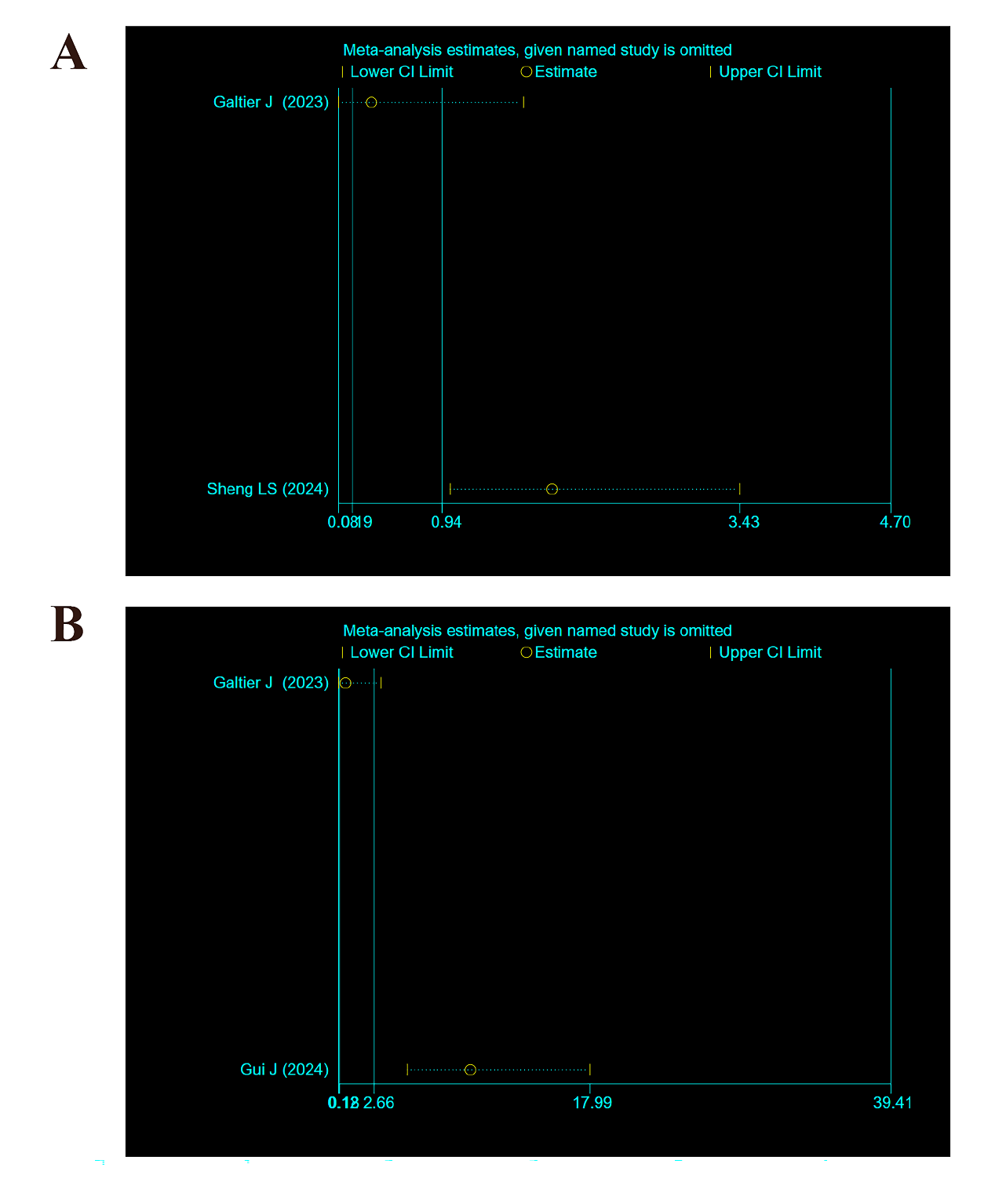


**Figure S29** Sensitivity analysis of the prognostic effect of LDH (A) and DS (B) on progression-free survival.


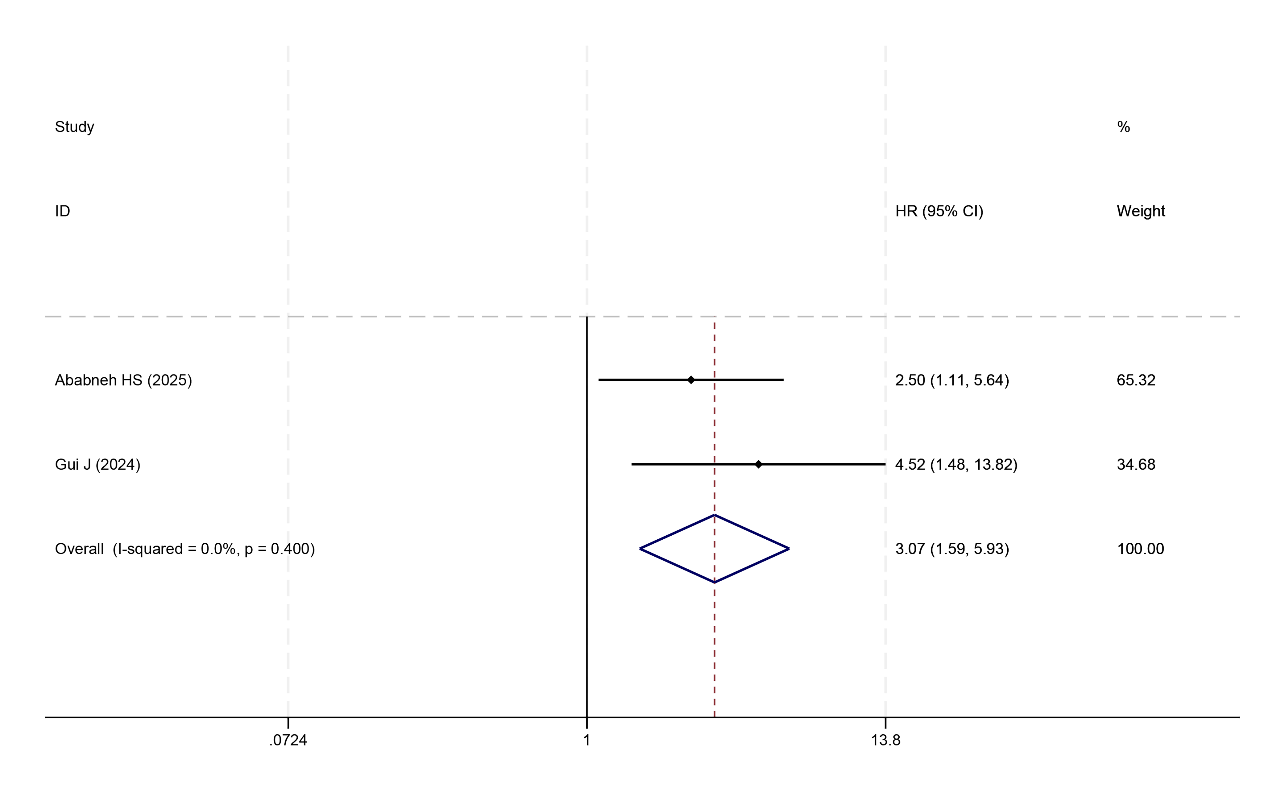


**Figure S30** Forest plot of the meta-analysis on the prognostic effect of IPI on progression-free survival.


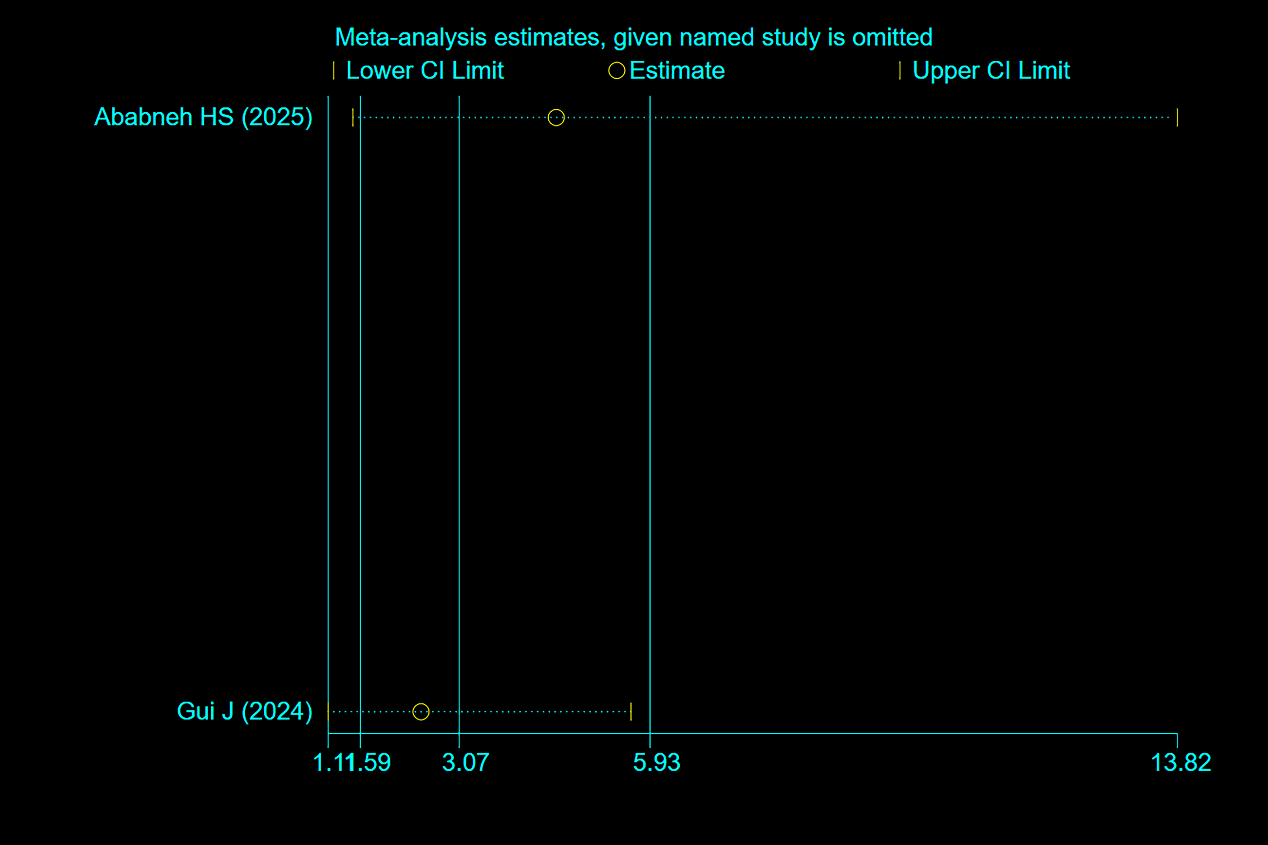


**Figure S31** Sensitivity analysis of the prognostic effect of IPI (A) on progression-free survival.
